# Expanding the Chemical Space of Electrophilic β-Glycosyl β-Lactams through Photoinduced Diastereoselective Functionalization

**DOI:** 10.1021/acs.orglett.4c01844

**Published:** 2024-06-20

**Authors:** Éverton A. Tordato, Renan O. Gonçalves, Lucas L. Baldassari, Claudio A. Jiménez, Diogo S. Lüdtke, Márcio W. Paixão

**Affiliations:** † Laboratory for Sustainable Organic Synthesis and Catalysis - Chemistry Department − Federal University of São Carlos − UFSCar, São Carlos, São Paulo 13565-905, Brazil; ‡ Institute of Chemistry, Federal University of Rio Grande do Sul - UFRGS, Porto Alegre, Rio Grande do Sul 91501-970, Brazil; § Department of Organic Chemistry, Faculty of Chemical Sciences, Universidad de Concepción, Concepción 4130000, Chile

## Abstract

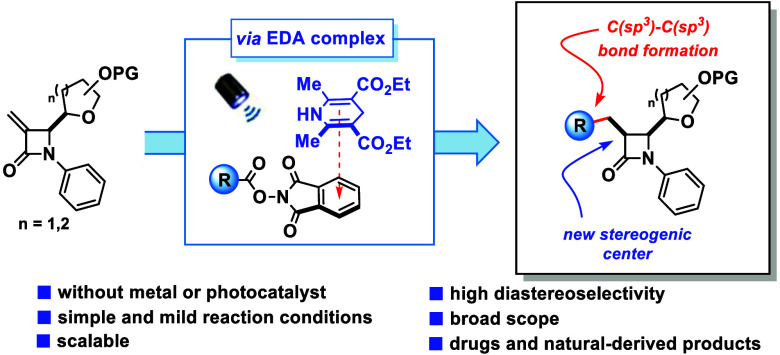

Herein, we present a photoinduced diastereoselective C-3 functionalization of electrophilic β-glycosyl β-lactams. The developed protocol is simple, mild, and scalable and explores the use of 3-exomethylene β-lactams as reaction partners in a Giese type reaction. The key nucleophilic alkyl radical is generated by a photoinduced electron transfer process in the EDA complex formed by NHPI and Hantzsch esters. The diastereoselective hydrogen atom transfer to the β-lactam radical intermediate enables the synthesis of various *N*-phenyl β-glycosyl β-lactams.

The β-lactam motif holds a significant role in drug discovery, garnering increased attention as a biological target for therapeutic development.^1^ Among the various types of 2-azetidinones, the *N*-phenyl β-lactams stand out for their activity in many different fields of biology (Scheme 1A).^2^ While the biological activity of β-lactams is well recognized, their decoration with carbohydrate units through selective glycosylation reactions can further enhance their performance. Glycosylation brings significant changes to the biological and physicochemical properties of small molecules, potentially having a profound impact on their solubility, activity, and bioavailability.^3^ For instance, adding a disaccharide to Ezetimibe’s structure, an inhibitor of cholesterol absorption, increases its potency 4-fold (Scheme 1B).^4^

**Scheme 1:**
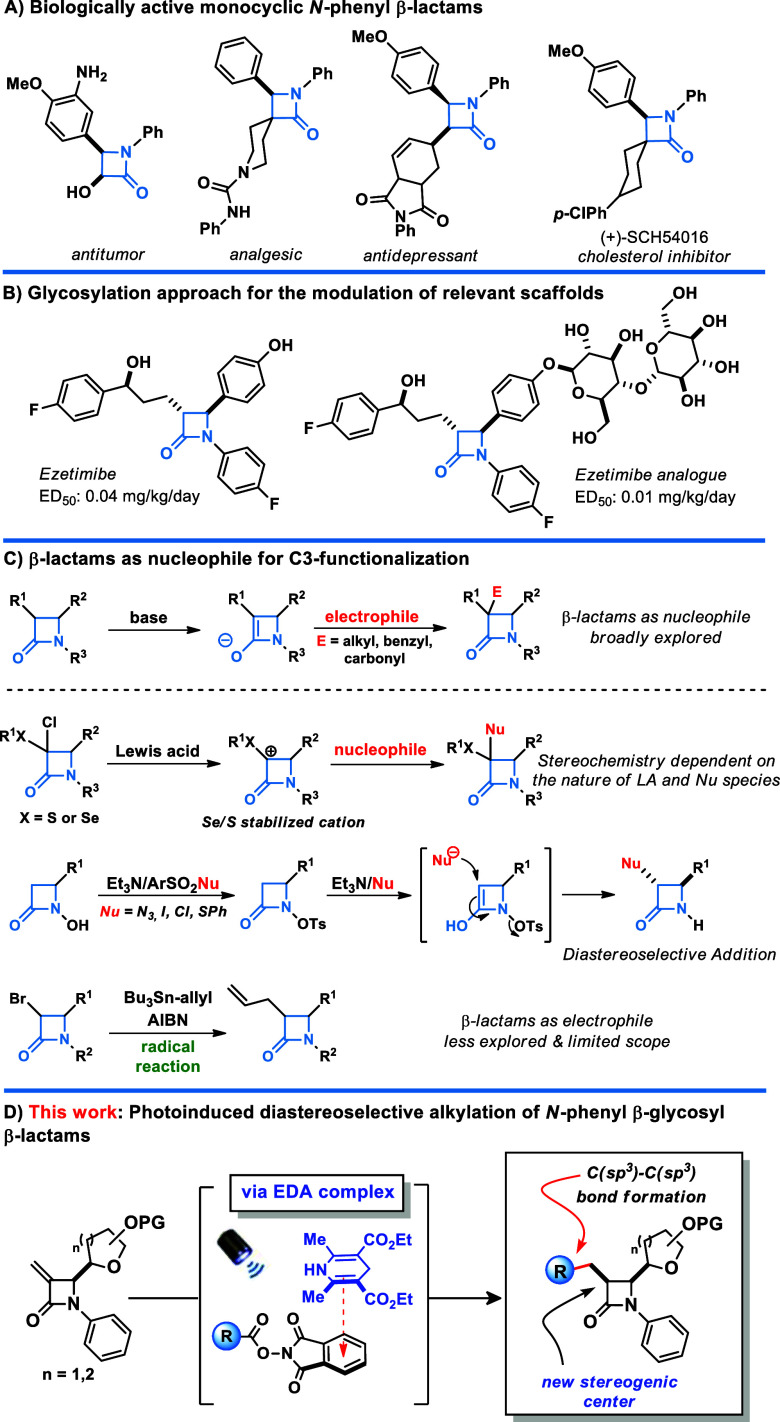
Literature Precedents and Outline

In light of its prevalence as the foundational structure in a myriad of biologically relevant molecules, substantial efforts have been directed toward developing sustainable synthetic methodologies for the β-lactam assembly.^5^ Moreover, this moiety assumes a pivotal position as an advanced intermediate for the synthesis of amino acids and peptides, as well as in preparing heterocycles, taxoids, and alkaloids.^2b^ Its versatile presence underscores its significance in organic synthesis, serving as a linchpin for constructing complex molecular architectures.^1c^

The synthesis of the β-lactam ring commonly employs cycloaddition protocols such as those involving ketene–imine, alkyne-nitrone, ester-enolate, and alkene-isocyanate, as well as reactions promoted by visible light.^6^ These approaches allow the preparation of β-lactams with functionalization possibilities across all positions. However, achieving this level of chemical diversity requires appropriately substituted substrates, which are often challenging to assemble. Consequently, direct modification of the β-lactam core, particularly at position 3, is increasingly recognized as crucial, given its direct correlation with the biological efficacy of these compounds.^7^ Among the available methods for C-3 functionalization in 2-azetidinones, the base mediated functionalization of β-lactams via the carbanion intermediate is broadly studied; however, limited by the requirement of stringent reaction conditions.^8^ On the other hand, the C-3 carbocation equivalent method, in which β-lactams act as electrophiles, is less explored and presents a limited substrate scope. Moreover, the single-electron-driven functionalization of this scaffold is still less explored, although it has great potential for development due to its ability to selectively promote the formation of structurally complex compounds (Scheme 1C).^9^ Recently we,^10^ and others,^11^ have been involved in developing photoinduced processes enabled by EDA complexation. Inspired by these previous studies and with the aim of filling this methodological gap, herein we present an unprecedented diastereoselective C-3 functionalization of electrophilic β-glycosyl β-lactams. This metal/photocatalyst free procedure is simple, mild, biocompatible, scalable, and explores using 3-exomethylene β-lactams, which are still unexplored under photochemical conditions, combined with an NHPI ester as the radical donor.^12^ To this end, the designed photochemical protocol enables the synthesis of *N*-phenyl β-glycosyl β-lactams bearing drugs and natural products in a high diastereoselective fashion (Scheme 1D).

At the outset, we have selected β-glycosyl β-lactam **2e** and NHPI ester **1e** as model substrates to optimize the reaction conditions. These experiments were conducted in the presence of Hantzsch Ester (HE) and under blue LED irradiation (Table 1). In initial experiments employing standard conditions - **1e** (1.5 equiv), **2e** (1.0 equiv), HE (1.5 equiv), blue LED 40 W, 2 h, 25 °C in DMSO (0.15 M) - the desired product **3e** was isolated in 50% yield with an excellent diastereoselectivity of >20:1 (entry 1) in favor of the *cis* product. Furthermore, a solvent screening was carried out, showing that DMSO outperformed all other solvents investigated (entries 2 and 3). Attempts using different stoichiometric quantities of the HE and NHPI ester **1e** reagents also did not improve chemical yield, even in longer reaction times (entries 4–7). Degassing the reaction solvent was also examined, resulting in a lower yield for the formation of compound **3e** (entry 8). Under a different light source (390 nm/40 W), a slight decrease in yield was observed, along with a significant drop in selectivity (entries 9–10). We then performed the reaction in the dark or the absence of HE as control experiments, and in both conditions, the formation of the desired product was not observed (entry 11). Therefore, the standard condition outlined in entry 1 was chosen for further studies and screening of the substrate scope.

**Table 1:**
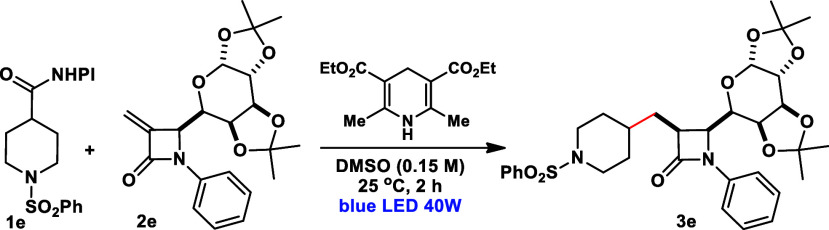
Optimization of the Reaction Conditions^a^,^g^

Entry	Deviation from the standard condition	Yield (%)^b^	d.r.^b^
1	none	50^c^	>20:1^c^
2^d^	DMAc	42	6:1
3^d^	MeCN	trace	-
4^d^	HE = 2 equiv.	48	>20:1
5^d^	NHPI ester = 2 equiv.	46	>20:1
6^d^	HE and NHPI ester = 1.1 equiv. each	43	>20:1
7^d^	HE and NHPI ester = 2.2 equiv. each	47	19:1
8^d^	degassed	43	>20:1
9	390 nm/40 W	43	19:1
10^e^	390 nm/40 W	46	14:1
11^f^	control	n.r.	-

a *Standard conditions*: **1e** (1.5 equiv), **2e** (1.0 equiv), **HE** (1.5 equiv), blue LED 40 W, 2 h, 25 °C, DMSO (0.15 M).

b Determined by ^1^H NMR of the crude product using 1,3,5-trimethoxybenzene as internal standard.

c Isolated yields determined by the average of the triplicate.

d Reaction time, *t* = 24 h, DMSO [0.10 M].

e HE and NHPI ester (1.1 equiv. each), DMSO (0.20 M), *t* = 4 h.

f Control experiment, without HE or light source.

g *Legend:* HE = Hantzsch ester and NHPI = *N*-hydroxyl phthalimide, n.r. = no reaction.

With suitable reaction conditions in hand, the scope study was further performed to examine the potential of the newly developed photoinduced reaction. In this regard, several NHPI esters and β-glycosyl β-lactams were evaluated to confirm the methodology’s efficiency and generality (Scheme 2). A range of cyclic and acyclic NHPI esters, resulting in primary and secondary radicals, were employed in the reaction, forming products with good yields and excellent diastereoselectivity (**3a**-**3f**). Particularly noteworthy is compound **3c**, derived from the natural compound lithocholic acid, whose product was formed tolerating the presence of an unprotected hydroxyl group in 45% yield and *d.r.* > 20:1. NHPI esters derived from pharmaceutical active ingredients were also utilized, leading to the efficient synthesis of diclofenac and indomethacin β-lactam derivatives **3j** (51% yield), and **3k** (46% yield, 14% of starting material recovery), respectively. The reaction also showed remarkable tolerance to protected alcohols (**3n**) and amines (**3l**-**3m**) as well as aromatic (**3i**) and steric hindered substrates (**3g**-**3h**). An intriguing example could be obtained by employing NHPI derived from D-galactose bis-acetonide, yielding compound **3n** adorned with two carbohydrate units at C-3 and C-4 within the product’s structure.

**Scheme 2:**
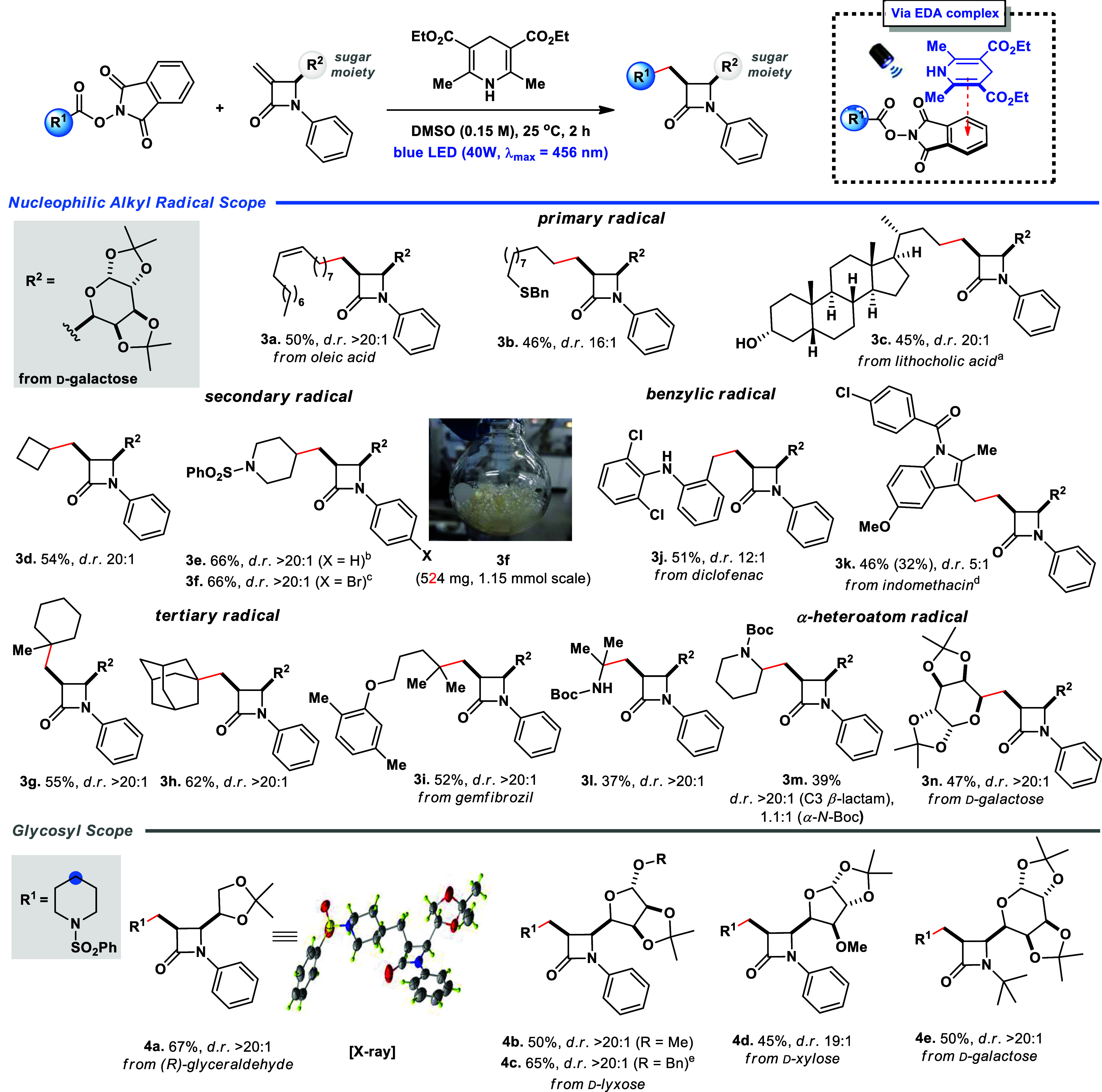
Scope of the Nucleophilic Alkyl Radical and Glycosyl Moiety 0.5 mL of THF was used to solubilize **1c**. Reaction performed on a 0.82 mmol scale. Reaction performed on a 1.15 mmol scale. Reaction did not finish even after 7 h. Starting material (14%) was recovery together with the alkylation product. Reaction performed on a 0.49 mmol scale. Unless otherwise specified, all reactions were performed on a 0.2 mmol scale.

The glycosidic moiety was also investigated, and variations in the carbohydrate units present in the β-lactam core were evaluated. The reaction tolerated the presence of (*R*)-glyceraldehyde (**4a**), D-xylose (**4d**), and its epimer D-lyxose (**4b**-**4c**), maintaining excellent diastereoselectivity and achieving good yields, especially for compound **4c** (65%). Finally, it is noteworthy that using the *N*-*tert*-butyl group at the 3-exomethylene β-lactam was also efficient for forming the compound derived from D-galactose (**4e**). ^1^H NMR assigned the *cis* stereochemistry of all products **3a-n** and **4a-e** and further unequivocally confirmed by X-ray diffraction analysis of the crystal structure of compound **4a**.

We then decided to evaluate the scalability of this new diastereoselective protocol for C-3 functionalization of electrophilic *N*-phenyl β-glycosyl β-lactams. Applying similar conditions to those previously utilized, a 1.15 mmol scale experiment employing the *N*-SO_2_Ph-NHPI ester successfully afforded alkylated product **3f** in 66% yield and excellent diastereoselectivity.

To highlight the utility of the synthesized compounds, we have subjected compound **3e** to a reaction with sodium methoxide (Scheme 3), leading to the formation of corresponding β-glycoamino ester product **5** in 66% yield and maintaining the stereochemical purity obtained in the photoinduced radical addition.

**Scheme 3:**
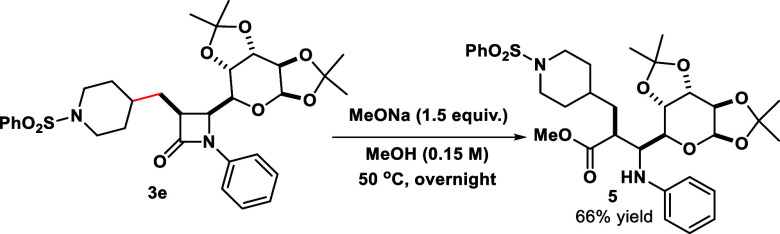
Synthetic Application of Product **3e**

Based on our previous studies^10a,10b^ and literature reports^13^ a plausible reaction mechanism for this transformation is proposed in Scheme 4. When the EDA complex formed between, HE and NHPI ester **1** absorbs light, a photoinduced electron transfer process takes place, forming the reduced NHPI ester and the radical cation of the oxidized Hantzsch ester. Next, the reduced NHPI ester undergoes fragmentation to release carbon dioxide and generates R^1^ radical. The following step involves the radical conjugated addition of R^1^ radical species to the 3-exomethylene β-lactam **2**, affording a tertiary radical intermediate. Finally, this intermediate is quenched through a hydrogen atom transfer from the Hantzsch ester radical cation, forming the desired alkylated C-3 *N*-phenyl β-glycosyl β-lactam. The stereochemistry-determining step occurs via a HAT from the less hindered face of the β-lactam, *trans* to the sterically more demanding R^2^ group, therefore leading to the observed *cis* stereochemistry for the final product **3**. This step could experimentally be demonstrated through the reaction in the presence of the radical scavenger (TEMPO), where the intermediate highlighted in the box was effectively trapped and detected by mass spectrometry (see Supporting Information for details).

**Scheme 4:**
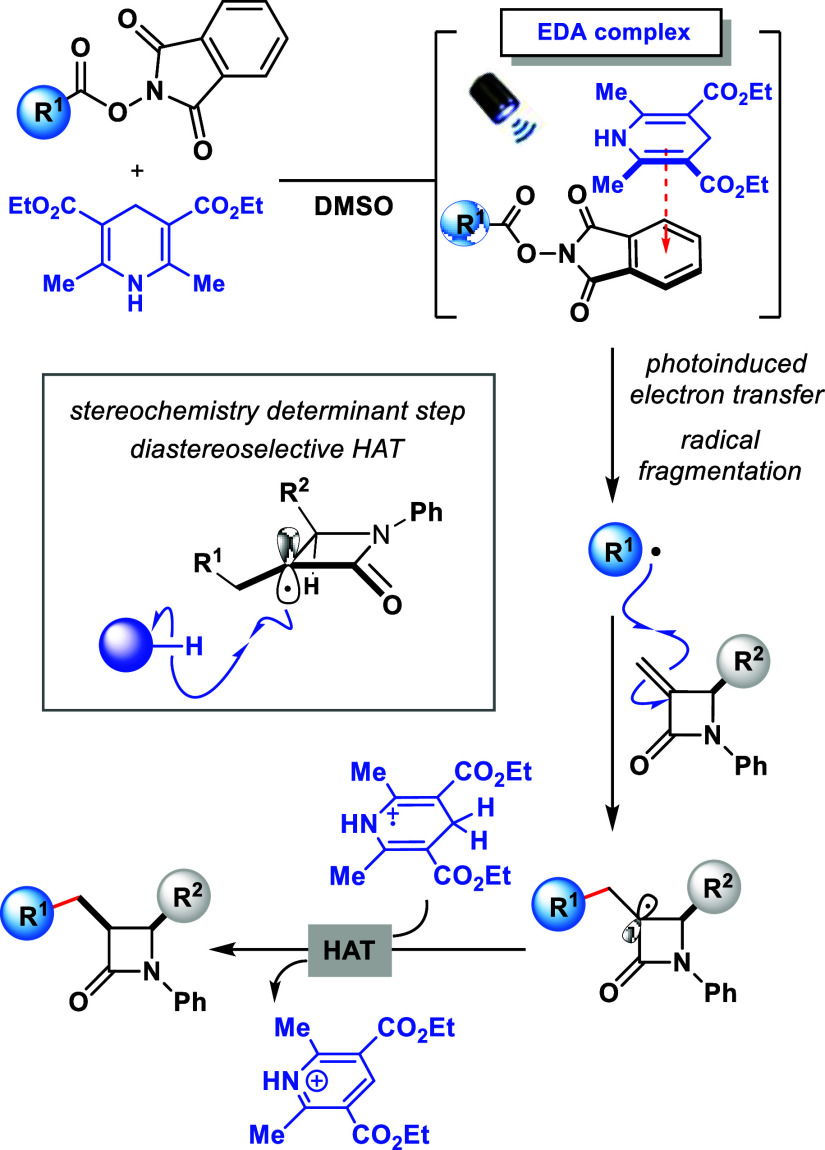
Mechanistic Rationale and Stereochemistry Determinant Step

In summary, we have reported a mild and efficient approach for synthesizing *N*-phenyl β-glycosyl β-lactams bearing drugs and natural products. This protocol is based on metal/photocatalyst free diastereoselective functionalization of electrophilic 3-exomethylene β-lactams with NHPI esters, through EDA complex formation. This strategy features excellent functional group tolerance, scalability, and high diastereoselectivity and offers an alternative way to functionalize position C-3 in the β-lactams core.

## Data Availability

The data underlying this study are available in the published article and its Supporting Information.

## Supporting Information Available

Supplementary data containing full analytical data and copies of NMR spectra (PDF)
