# Supplementary material for: Expanding the Chemical Space of Electrophilic β-Glycosyl β-Lactams through Photoinduced Diastereoselective Functionalization
Source: Org Lett. 2024 Jun 20;26(26):5500–5. doi: 10.1021/acs.orglett.4c01844 (PMC11232025; doi:10.1021/acs.orglett.4c01844)
Supplement: Supplementary file 1 — ol4c01844_si_001.pdf [file ol4c01844_si_001.pdf]

Supporting Information

Expanding the Chemical Space of Electrophilic β-Glycosyl β-lactams through Photoinduced Diastereoselective Functionalization

Éverton A. Tordato,[a] Renan. O. Gonçalves,[a] Lucas L. Baldassari,[a,b] Claudio A. Jiménez,[c] Diogo S. Lüdtke,[b,*] and Márcio W. Paixão,[a,*]

[a] Laboratory for Sustainable Organic Synthesis and Catalysis - Chemistry Department – Federal University of São Carlos – UFSCar, São Carlos, São Paulo, 13565-905, Brazil. E-mail: mwpaixao@ufscar.br

[b] Institute of Chemistry, Federal University of Rio Grande do Sul - UFRGS, Porto Alegre, Rio Grande do Sul, 91501-970, Brazil. E-mail: dsludtke@iq.ufrgs.br

[c] Department of Organic Chemistry, Faculty of Chemical Sciences, Universidad de Concepción, Concepción, 4130000, Chile

Summary

1. General Informations 3
2. General procedures for substrates syntheses 4
   2.1 Hantzsch ester 4
   2.2 *N*-(acyloxy)phthalimides (redox-active esters) 4
      2.2.1 Spectroscopic data of the compounds 7
   2.3 *N*-phenylhydroxylamine and *N*-(4-bromophenyl)hydroxylamine 8
   2.4 *N*-*tert*-butylhydroxylamine 9
   2.5 Protected monosaccharides aldehydes 10
   2.6 Nitrones (Nit.1 – Nit. 7) 16
      2.6.1 Spectroscopic data of the compounds 17
   2.7 3-exomethylene β-glycosyl β-lactam (2a – 2g) 20
      2.7.1 Spectroscopic data of the compounds 21
   2.8 Photoinduced reaction via EDA complex (3a – 3n; 4a – 4e) 25
      2.8.1 Spectroscopic data of compounds 25
   2.9 Synthetic application 36
      2.9.1 Spectroscopic data of the compound 36
3. Mechanistic studies 37
   3.1 UV/Vis absorption spectroscopy 37
   3.2 NMR titration 39
   3.3 Radical trapping experiment 40
4. Scale-up of the photoinduced EDA reaction 41
5. X-ray analysis parameters 42
6. NMR spectra 45
7. References 78

1. General Informations

All solvents were dried and distilled before use by standard procedures[1]. The reagents were purchased from Êxodo Científica, Sigma-Aldrich, TCI Chemicals, AmBeed and used as received. All air- or moisture-sensitive reactions were carried out in flame-dried glassware cooled under nitrogen atmosphere. The photochemical experiments were carried out using a 40 W Kessil H150 blue LED (λ_{max} = 456 nm) as a visible light source in Schlenk flasks. The reactions were monitored using thin-layer chromatography (TLC) silica gel on aluminum foils with fluorescent indicator 254 nm (Supelco) with a thickness of 0.20 mm. The plates were revealed under UV light (254 nm) and, when necessary, treated with potassium permanganate, thymol or vanillin. All compounds were purified on column chromatography with silica gel (60 Å pore size, 40–63 µM particle size) using appropriate mobile phases as described for each compound. The diastereoselectivities ratios (*d.r.*) refers to the isolated products. ^{1}H and ^{13}C NMR spectra were recorded on Brucker NMR spectrometer (400 MHz for ^{1}H and 100 MHz for ^{13}C. The chemical shifts (δ) for the ^{1}H and ^{13}C NMR spectra are in ppm and the residual solvent signals were used as reference for the ^{1}H and ^{13}C NMR spectra, non-deuterated chloroform ($CDCl_3$): δ H = 7.26 ppm, δ C = 77.16 ppm). The values of the coupling constant(s) *J* are given in Hertz. Multiplicities are described as: s = singlet, d = doublet, t = triplet, q = quartet, dd = doublet of doublets, dt = doublet of triplets, dq = doublet of quartets, m = multiplet. High resolution mass spectra (HRMS) were recorded at Waters Technologies from Brazil using a Xevo G2 XS QTOF (ESI-QTOF) spectrometer.

2. General procedures for substrates syntheses

2.1 Hantzsch ester[2]

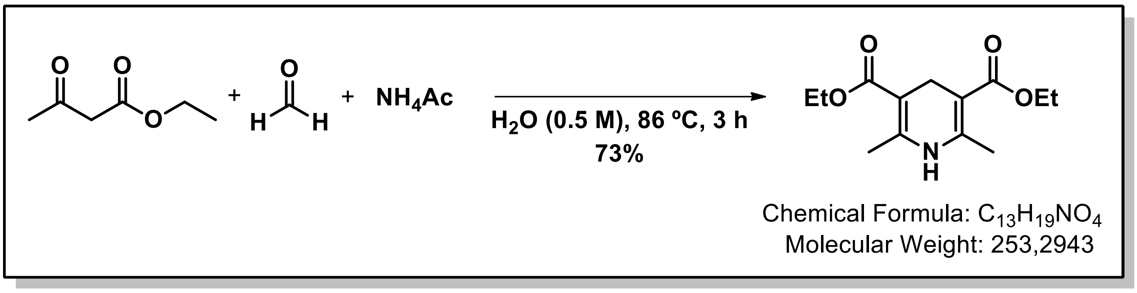

Scheme S1: Preparation of Hantzsch ester

A reaction mixture consisting of 37% formaldehyde in water (35 mmol, 1 equiv.; 2.63 mL), ethyl acetoacetate (140 mmol, 4 equiv.; 18 mL) and NH_4OAc (70 mmol, 2 equiv.; 5. 4 g) in H_2O (70 mL; 0.5 M) at 86 ºC in an oil bath was stirred for 3 hours (product precipitates during the reaction). After completion, the solution was cooled to room temperature, filtered under vacuum in a sintered plate funnel or Büchner funnel. The solid formed was washed with ice-cold acetone and allowed to dry at room temperature in the fume hood overnight and under vacuum for a few hours, providing a fine yellow solid in 73% yield (25.6 mmol; 6.5 g).

2.2 *N*-(acyloxy)phthalimides (redox-active esters)[3]

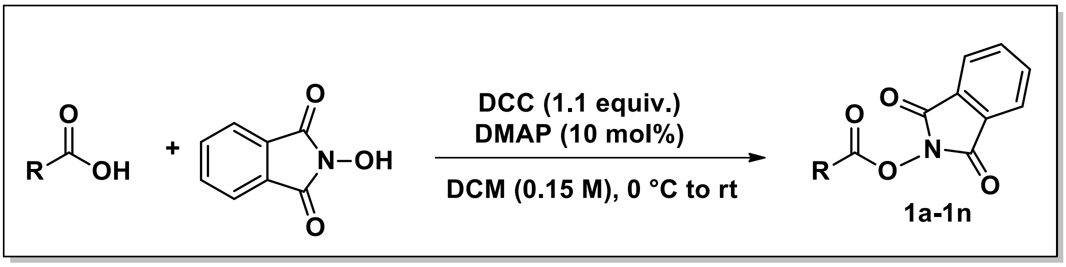

Scheme S2: Preparation of *N*-(acyloxy)phthalimides

N-(acyloxy)phthalimides were synthesized using an adapted Steglich esterification protocol. To a solution of carboxylic acid (3 mmol) in DCM (0.15 M) in an ice-bath were added DCC (3.3 mmol, 1.1 equiv.; 687 mg), DMAP (10 mol%, 0.3 mmol, 37 mg) and *N*-hydroxyphthalimide (3.3 mmol, 1.1 equiv.; 538 mg). After the reaction solution was stirred overnight, it was filtered to remove the precipitate formed, which was washed with DCM (80 mL). The combined organic phases were washed with aqueous saturated solution of $NaHCO_3$

(2 x 30 mL), 10% HCl solution (2 x 30 mL) and brine (1 x 50 mL). The organic phase was dried over anhydrous Na_2SO_4, filtered and the solvent evaporated under reduced pressure. The crude product was purified on flash column chromatography, providing *N*-(acyloxy)phthalimides in yields from 50 to 80%.

The *N*-(acyloxy)phthalimides **1a**, **1c**, **1d**, **1g** and **1h** were obtained in a single synthetic step, starting from the corresponding commercial carboxylic acids. Substrate **1b** was obtained in two steps: benzylation[4] and esterification. Substrate **1e**, **1l** and **1m** was obtained in two steps: protection of the amino group[5] and esterification. Substrates **1i**, **1j**, and **1k** were obtained from maceration and extraction with EtOAc of their active pharmaceutical ingredients (API) from commercial drugs when in their neutral form or previously treated by adjusting the pH to recover their API and subsequent extraction with EtOAc. The tablets (3 grams) were macerated and the powder obtained was kept under stirring with EtOAc (100 mL) for 30 minutes. After that, the solution was filtered and the solvent evaporated, affording the API in yields around 93%. They were then subjected to the esterification reaction. Substrate **1n** was obtained from the protection reaction of its hydroxyls[6] (adapted procedure), oxidation[7] of the primary hydroxyl to carboxylic acid and esterification.

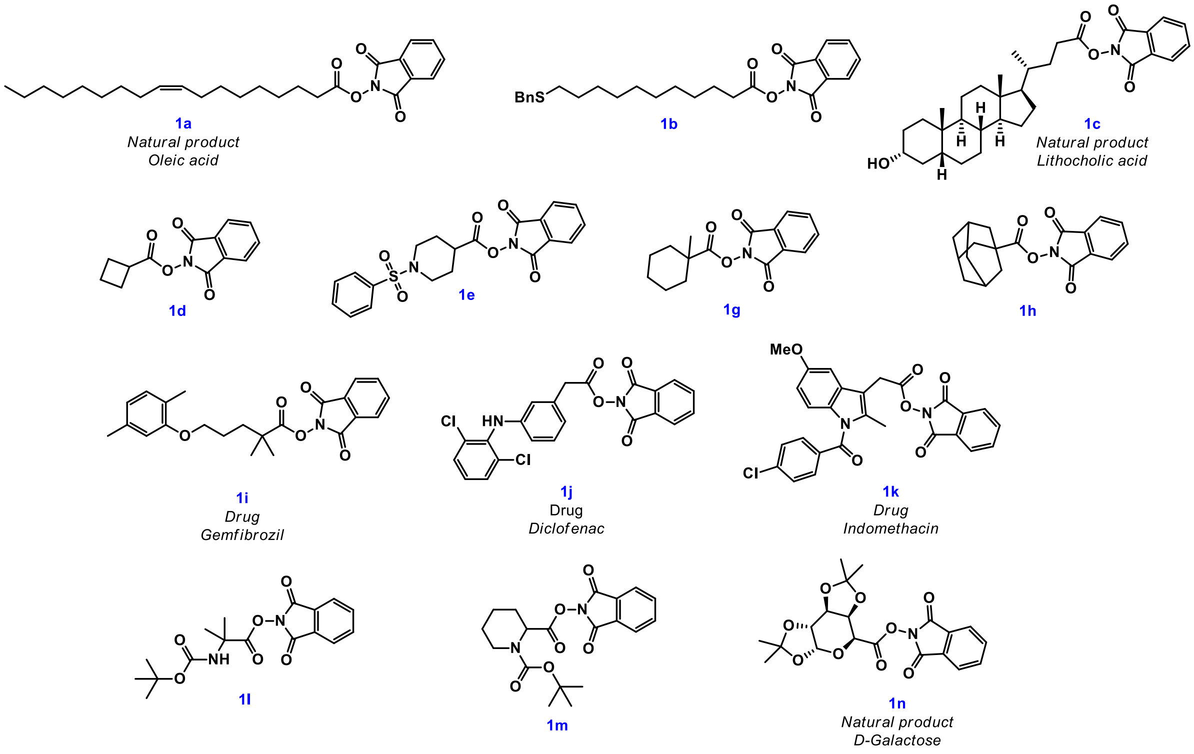

Scheme S3: Redox-active esters used in this work as radical sources

Synthesis of N-(acyloxy)phthalimide (1a)

Obtained from the esterification protocol described above as a colorless oil with 80% yield (2.4 mmol; 1.03 g).

Synthesis of *N*-(acyloxy) phthalimide (1b)

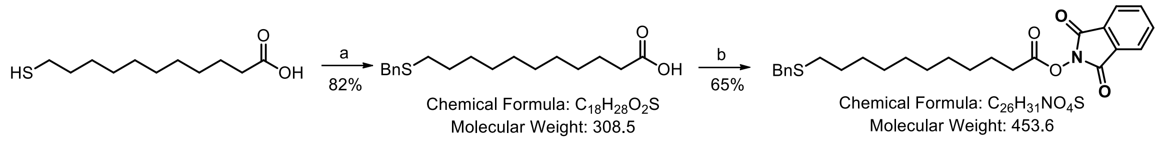

Reagents and Conditions: a) NaOH 2.0 M (0.5 M), BnBr (1 equiv.), EtOH (0.2 M), rt, 3 h; b) *N*-OH-Phth (1.1 equiv.), DMAP (10 mol %), DCC (1.1 equiv.), DCM (0.15 M), 0 ºC - rt, overnight.

Scheme S4: Preparation of **1b**

First step (benzylation)[4]: To an aqueous solution of NaOH (2 M, 6 mL) and EtOH (15 mL), 11-mercaptoundecanoic acid (3 mmol, 1 equiv.; 655 mg) and BnBr (3 mmol, 1 equiv.; 0.360 mL) were added. The reaction solution remained under vigorous stirring for 3 hours. Soon after, it was acidified with HCl until pH 2. The ethanol was evaporated and the aqueous phase extracted with EtOAc (3 x 50 mL). The organic phases were combined and washed with brine (1 x 50 mL), dried over anhydrous Na_2SO_4, filtered and evaporated under reduced pressure, providing a waxy white solid in 82% yield (2.46 mmol, 760 mg). This product was used in the esterification step without further purification. The esterification step was carried out as described above. The product was isolated by flash column chromatography as a yellow viscous oil which solidified in the freezer as a waxy yellow solid in 65% yield (1.6 mmol, 725 mg).

Synthesis of *N*-(acyloxy) phthalimide (1e)

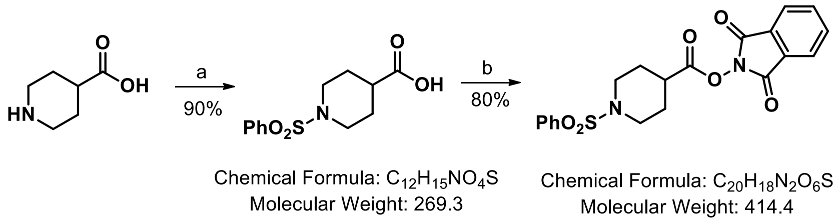

Reagents and Conditions: a) $NaHCO_3$ (1.5 equiv.), Na_2CO_3 (1.5 equiv.), $PhSO_2Cl$ (1.0 equiv.), MeCN:H_2O (2:3; 0.12 M), 0 ºC - rt, 18h. b) N-OH-Phth (1.1 equiv.), DMAP (10 mol %), DCC (1.1 equiv.), DCM (0.15 M), 0 ºC - rt, overnight.

Scheme S5: Preparation of **1e**

First step (protection)[5]: To a solution of isonipecotic acid (7.74 mmol, 1 equiv.; 1 g) in MeCN:H_2O (2:3; 0.12 M, 65 mL) in an ice bath, was added $NaHCO_3$ (11.6 mmol, 1.5 equiv.; 974 mg) and Na_2CO_3 (11.6 mmol, 1.5 equiv.; 1.23 g). This reaction solution was stirred for 15 minutes. After that, $PhSO_2Cl$ (7.74 mmol, 1 equiv.; 1 mL) was added dropwise. It was allowed to stir for 18 hours with a gradual return to room temperature. At the end, the reaction solution was placed back in an ice bath and acidified with HCl (1M) until pH 2, precipitating a portion of the product that was recovered by filtration. The acidified reaction solution was then extracted with EtOAc (3 x 70 mL) and the combined organic phases were washed with brine (1 x 50 mL), dried over anhydrous Na_2SO_4, filtered and evaporated, giving a white solid in 90% yield (together with precipitated material). This product was used in the esterification step without further purification (6.98 mmol; 1.88 g). The esterification step was carried out as described above. The product was isolated as a flaky white solid in 80% yield (5.6 mmol; 2.3 g). Mobile phase – Gradient: DCM in Hexanes (70 – 100%). R_f = 0.4 (100% DCM).

2.2.1 Spectroscopic data of the compounds

The spectroscopic data of the new *N*-(acyloxy)phthalimides are reported below:

1,3-dioxoisoindoline-2-yl oleate (1a)

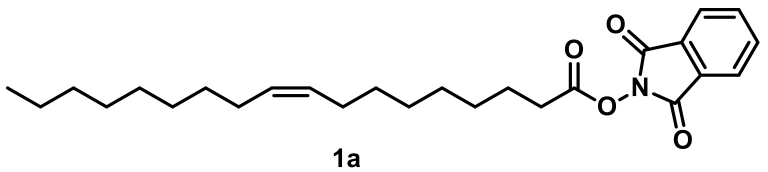

1a

^{1}H NMR (400 MHz, $CDCl_3$) δ 7.65 – 7.56 (m, 2H), 7.54 – 7.49 (m, 2H), 5.15 – 5.05 (m, 2H), 2.40 (t, *J* = 7.4 Hz, 2H), 1.77 (s, 4H), 1.57 – 1.49 (m, 2H), 1.22 – 1.16 (m, 2H), 1.11 – 0.99 (m, 18H), 0.62 (t, *J* = 6.3 Hz, 3H) ppm. **^{13}C NMR (101 MHz, $CDCl_3$)** δ 169.6, 162.0, 134.7, 130.0, 129.7, 129.0, 123.9, 77.5, 77.2, 76.8, 31.9, 31.0, 29.8, 29.7, 29.6, 29.4, 29.1, 29.0, 28.8, 27.25, 27.2, 24.7, 22.7, 14.1 ppm. **HRMS** (ESI) $[M + Na]^+$ m/z: calculated for $C_{26}H_{37}NO_4Na$ 450.2615, found: 450.2613.

1,3-dioxoisoindolin-2-yl 11-(benzylthio) undecanoate (1b)

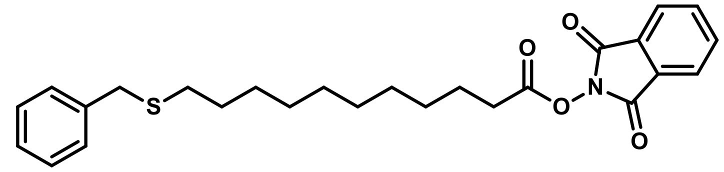

1b

^{1}H NMR (400 MHz, $CDCl_3$) δ 7.95 – 7.86 (m, 2H), 7.86 – 7.77 (m, 2H), 7.33 -7.30 (m, 4H), 7.30 – 7.23 (m, 1H), 3.72 (s, 2H), 2.68 (t, *J* = 7.5 Hz, 2H), 2.43 (t, *J* = 7.4 Hz, 2H), 1.85 – 1.75 (m, 2H), 1.62 – 1.50 (m, 2H), 1.44 – 1.36 (m, 2H), 1.36 – 1.23 (m, 9H), 1.17 (d, *J* = 6.5 Hz, 1H) ppm. **^{13}C NMR (101 MHz, $CDCl_3$)** δ 169.8, 162.1, 138.8, 134.8, 129.1, 128.9, 128.6, 127.0, 124.1, 36.4, 31.5, 31.1, 29.5, 29.4, 29.33, 29.27, 29.2, 29.0, 28.9, 24.8 ppm. **HRMS** (ESI) $[M + Na]^+$ m/z: calculated for $C_{26}H_{31}NO_4SNa$ 476.1866, found: 476.1869

1,3-dioxoisoindolin-2-yl 1-(phenylsulfonyl) piperidine-4-carboxylate (1e):

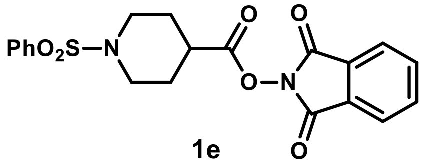

1e

^{1}H NMR (400 MHz, $CDCl_3$) δ 7.89 – 7.83 (m, 2H), 7.81 – 7.76 (m, 4H), 7.64 – 7.59 (m, 1H), 7.55 (t, *J* = 7.6 Hz, 2H), 3.65 (dt, *J* = 8.9, 4.2 Hz, 2H), 2.80 – 2.61 (m, 3H), 2.20 – 2.11 (m, 2H), 2.03 (dtd, *J* = 13.8, 10.0, 3.8 Hz, 2H) ppm. **^{13}C NMR (101 MHz, $CDCl_3$)** *δ* 170.2, 161.9, 136.2, 134.9, 133.0, 129.2, 128.9, 127.7, 124.1, 44.9, 37.5, 27.3 ppm. **HRMS** (ESI) $[M + Na]^+$ m/z: calculated for $C_{20}H_{18}N_2O_6SNa$ 437.0778, found: 437.0777

The spectroscopic data of the other *N*-(acyloxy)phthalimides have already been published in previous works and can be found in the following literature: **1c**[8], **1d** , **1g** and **1h**[9, 10], **1i**[11], **1j**[12], **1k** and **1l**[13], **1m**[14], **1n**[15, 16] and **1o**[17].

2.3 *N*-phenylhydroxylamine and *N*-(4-bromophenyl)hydroxylamine[18, 19]

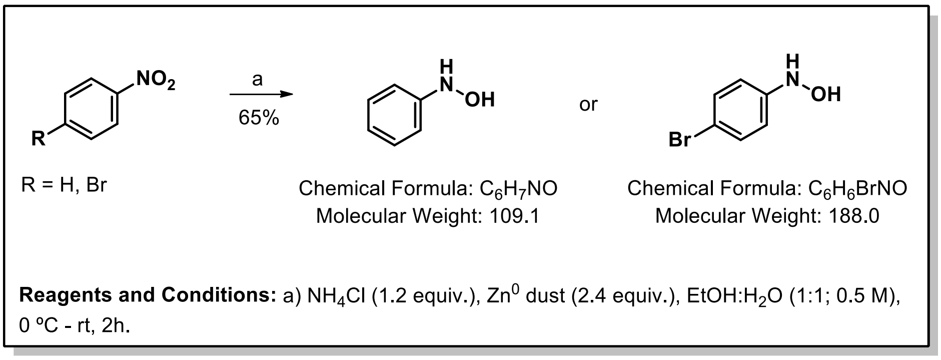

Scheme S6: Preparation of *N*-phenylhydroxylamines

To a solution of nitrobenzene (20 mmol; 1 equiv.; 2.06 mL), NH_4Cl (24 mmol; 1.2 equiv.; 1.3 g) EtOH:H_2O (1:1; 0.5 M, 40 mL) in an ice bath, powdered Zn^0 (48 mmol; 2.4 equiv.; 3.14 g) was added. This reaction solution was kept under vigorous stirring for 2 hours with a gradual return to room temperature. After that, it was filtered through filter paper or celite and the zinc salt washed with EtOAc (100 mL). The phases were separated in a separatory funnel and the aqueous phase was extracted with AcOEt (60 mL). The organic phases were combined, washed with brine (2 x 50 mL), dried over anhydrous Na_2SO_4, filtered and the solvent evaporated under reduced pressure until obtaining a brownish syrup. Hexane was added to this syrup for precipitation and subsequent filtration to obtain the product *N*-phenylhydroxylamine as a beige solid in 65% yield (13 mmol, 1.42 g). Purer products can be obtained by solubilizing this precipitate in a minimum volume of EtOAc and adding hexane and subsequently storing it in the freezer for a few hours. A needle-shaped crystalline solid is formed. This solid turn brownish in a short time at room temperature and even when stored in the freezer at -18 ºC, indicating its degradation. These compounds were prepared and used in the same day.

The spectroscopic data of the these *N*-phenylhydroxylamines have already been published in previous works and can be found in the following literature: *N*-phenylhydroxylamine - Melting point[20] and NMR[21], *N*-(4-bromophenyl)hydroxylamine[21].

Caution: *N*-phenylhydroxylamine causes respiratory and skin allergies and a risk of explosion[22].

2.4 *N-tert*-butylhydroxylamine

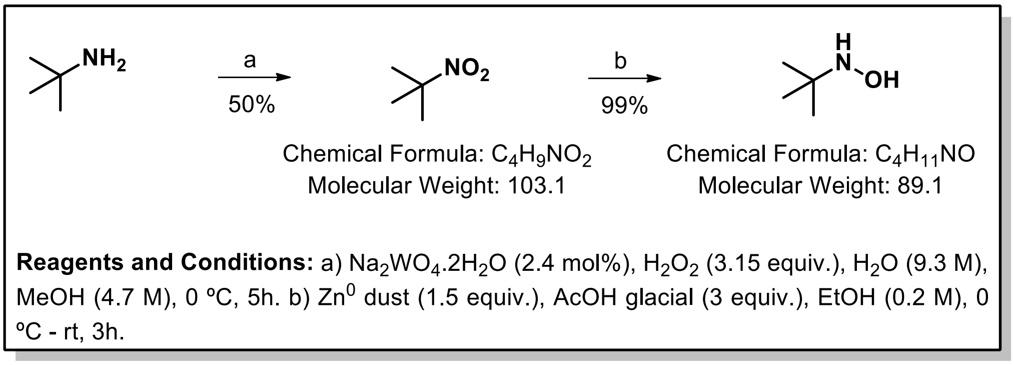

Scheme S7: Preparation of *N-tert*-butylhydroxylamine

<u>First step (oxidation)[23]:</u> In a solution of *N-tert*-butylamine (280 mmol, 1 equiv.; 30 mL), $Na_2WO_4.2H_2O$ (2.4 mol%, 6.72 mmol, 2.2 g) and H_2O (30 mL) at 0 ºC in an ice bath, H_2O_2 (30 %) (881 mmol, 3.15 equiv.; 90 mL) was slowly added (3 hours of addition). After 5 minutes of

starting the addition of H_2O_2, MeOH (60 mL) was added. The reaction solution acquires a bluish color. After finishing the addition of H_2O_2, the reaction solution remained under stirring for another 2 hours in an ice bath with a gradual return to room temperature. After that, H_2O (200 mL) was added and the reaction solution was stored in the fume hood for two days (disappearance of the bluish color). Then, this solution was extracted with DCM (4 x 150 mL), dried over anhydrous Na_2SO_4, filtered and the solvent evaporated under reduced pressure, yielding a colorless liquid with 50% (140 mmol, 14.4 g). This product was used in the next step without further purification.

Second step (reduction)[24, 25]: To a solution of 2-methyl-2 nitropropane (70 mmol, 1 equiv.; 7.22 g), Zn^0 dust (105 mmol, 1.5 equiv.; 6.86 grams) in EtOH (0.2 M, 350 mL) in an ice bath, AcOH glacial (210 mmol, 3 equiv.; 12 mL) was added dropwise using an addition funnel. After the addition of glacial AcOH was completed, the ice bath was removed and the reaction solution was kept under vigorous stirring for another 3 hours at room temperature. Then, the solution was filtered and the zinc salt formed was washed with DCM. The filtrate was evaporated under reduced pressure. The crude mass contained in the flask was resuspended in DCM, filtered and evaporated twice, affording a colorless viscous oil with quantitative yield (6.2 g).

The spectroscopic data have already been published in previous works and can be found in the following literature[26]

2.5 Protected monosaccharides aldehydes

(*R*)-2,2-dimethyl-1,3-dioxolane-4-carbaldehyde

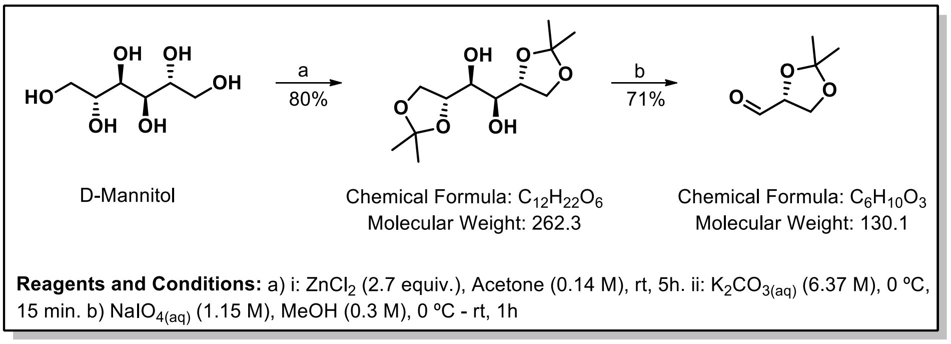

Scheme S8: Preparation of (*R*)-Gliceraldehyde

First step (protection)[27]: In a flask containing anhydrous $ZnCl_2$, acetone (400 mL) was added and the reaction mixture was stirred vigorously until complete homogenization. After the

solution was cooled to room temperature, *D*-mannitol (10 g; 55 mmol) was added and the solution remained under vigorous stirring for 5 h until the solution became clear. The reaction solution was placed in an ice bath and then a solution of K_2CO_3 (22 g) in H_2O (25 mL) was added. This reaction mixture was vigorously stirred for 15 min. The zinc carbonate was filtered and washed with acetone. The filtrate was concentrated and the white solid formed was dissolved in Et_2O (50 mL) and transferred to a separatory funnel. The organic phase was separated and the aqueous phase extracted with Et_2O (3 x 50 mL). The organic phases were combined, dried over anhydrous Na_2SO_4, filtered and the solvent evaporated under reduced pressure affording a white solid in 80% yield (44 mmol, 11.5 g).

Second step (1,2-diol oxidative cleavage)[28]: To a solution of 1,2 diol (20.9 mmol, 1 equiv.; 5.48 g) in MeOH (70 mL, 0.3 M) cooled in an ice bath, a solution of $NaIO_4$ (23 mmol 1.1 equiv.; 4.93 g) solubilized in distilled H_2O (20 mL) was added dropwise, using an addition funnel. After the addition, the reaction solution was kept under vigorous stirring for 1 hour with gradual return to room temperature. It was then filtered and the white solid formed was washed with DCM. The solvent was evaporated under reduced pressure and the aqueous phase extracted with EtOAc (3 x 80 mL). The combined organic phases were dried over anhydrous Na_2SO_4, filtered and the solvent evaporated under vacuum, providing a colorless oil in 71% yield (14.8 mmol, 1.93 g). This product was used in the next step without further purification.

(3a*S*,4*S*,6*S*,6a*S*)-6-methoxy-2,2-dimethyltetrahydrofuro[3,4-*d*][1,3]dioxole-4-carbaldehyde

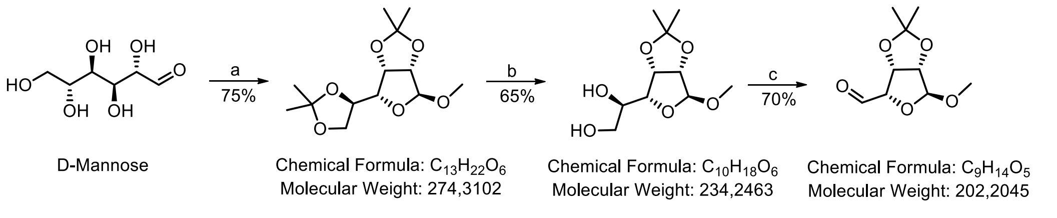

Reagents and Conditions: a) Acetone:MeOH (3:2 / 0.6 M), HCl 37% (0.24 M), 60 ºC, overnight. b) AcOH glacial: H_2O (3:2 / 0.15M), overnight. c) $NaIO_{4(aq)}$ (1.15 M), MeOH (0.3 M), 0 ºC - rt, 1h.

Scheme S9: Preparation of D-Lyxose-OMe aldehyde

First step (protection): To a solution of D-Mannose (30 mmol; 5.4 g) in acetone (30 mL) and MeOH (20 mL), 37% HCl (1 mL) was added. This reaction solution was stirred overnight

at 60 °C in an oil bath. After this time, it was left to cool to room temperature and then neutralized with $NaHCO_3$(s) and filtered. The white solid was washed with acetone. The organic phase was dried over anhydrous Na_2SO_4, filtered and evaporated, affording a yellow oil in 75% yield (22.5 mmol, 6.17 g). This crude product was used in the next step without further purification.

Second step (deprotection)[29]: The product obtained in the previous step (22.5 mmol; 6.17 g) was kept under vigorous stirring at room temperature for 16 hours in a solution of glacial AcOH/H_2O (3:2). After, the solution was evaporated under reduced pressure and washed with toluene (3 x 20 mL). The crude product was solubilized in EtOAc (120 mL) and the organic phase washed with saturated solution $NaHCO_{3(aq)}$ (2 x 50 mL) and brine (1 x 50 mL). The organic phase was dried over anhydrous Na_2SO_4, filtered and evaporated, affording a colorless oil in 65% yield (14.6 mmol, 3.43 g). This crude product was used in the next step without further purification.

Third step (1,2-diol oxidative cleavage)[28]: Next, the diol obtained in the previous step (14.6 mmol; 3.43 g) was subjected to the same reaction conditions, as described above. The aldehyde was obtained in 70% yield (10.2 mmol; 2.1 g) as a colorless viscous oil. This crude product was used in the next step without further purification.

(3a*S*,4*S*,6*S*,6a*S*)-6-(benzyloxy)-2,2-dimethyltetrahydrofuro[3,4-*d*][1,3]dioxole-4 carbaldehyde

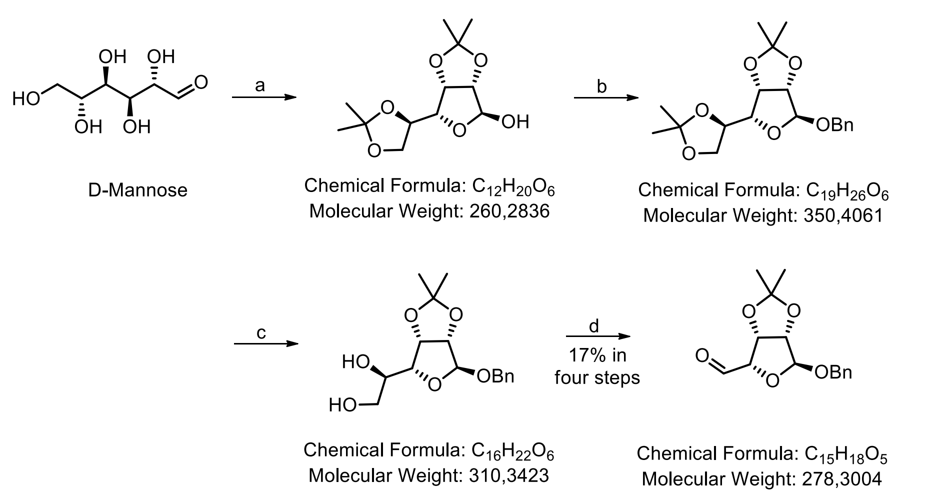

Reagents and Conditions: a) Acetone (0.5 M), HCl 37% (0.20 M), rt, 24 h. b) i: NaH (2 equiv.), DMF (0.3 M), ice-bath, N_2, 15 min. ii: BnCl (1.5 equiv.), ice-bath, 16h. c) AcOH glacial: H_2O (3:2; 0.15 M), overnight. d) $NaIO_{4(aq)}$ (1.15 M), MeOH (0.3 M), 0 ºC - rt, 1h.

Scheme S10: Preparation of D-Lyxose-OBn aldehyde

First step (protection): To a solution of D-Mannose (30 mmol; 5.4 g) in acetone (60 mL, 0.5 M), HCl 37% (1 mL) was added. This reaction solution was stirred at room temperature for 24 hours. After this time, it was neutralized with $NaHCO_3$(s) and filtered. The white solid was washed with acetone. The organic phase was dried over anhydrous Na_2SO_4, filtered and evaporated, providing a white solid in 30% yield (2.34 g). This crude product was used in the next step without further purification.

Second step (benzylation): The bis-acetonide (9 mmol, 1 equiv.; 2.34 g) obtained in the previous step was solubilized in anhydrous DMF, under N_2 atmosphere, and cooled in an ice bath. NaH (60% in mineral oil) was added (18 mmol, 2 equiv.; 720 mg) and the reaction solution remained under vigorous stirring for 15 minutes. After, BnCl (13.5 mmol, 1.5 equiv., 1.55 mL) was added dropwise and the reaction remained under vigorous stirring for another 16 hours with a gradual return to room temperature. At the end, the reaction solution was filtered and the solid formed was washed with DCM (150 mL). The organic phase was washed with brine (3 x 60 mL), dried over anhydrous Na_2SO_4, filtered and evaporated under reduced pressure, yielding a liquid (probably containing DMF).

Third step (deprotection)[29]: This step was carried out as described above, affording a colorless viscous oil in 75% yield. This crude product was used in the next step without further purification.

Fourth step (1,2-diol oxidative cleavage)[28]: Next, the diol obtained was subjected to the same reaction conditions, as described above. The aldehyde was obtained as a colorless viscous oil in 75% yield. This crude product was used in the next step without further purification.

(3a*R*,5*S*,6*S*,6a*R*)-6-methoxy-2,2-dimethyltetrahydrofuro[2,3-*d*][1,3]dioxole-5-carbaldehyde

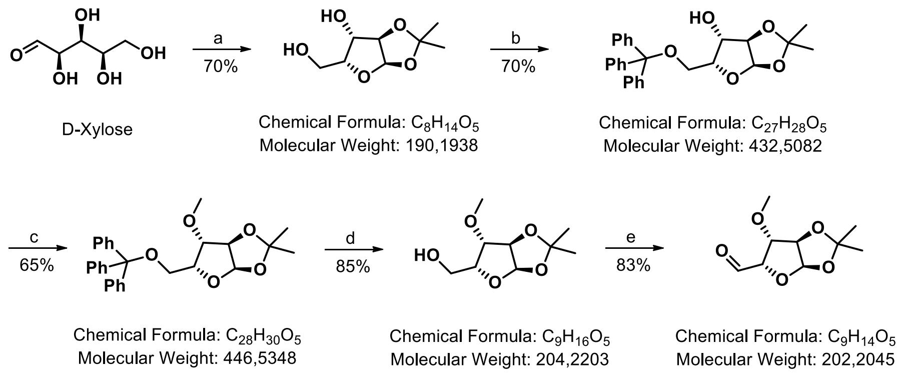

Reagents and Conditions: a) Acetone (0.2 M), H_2SO_4 98% (0.38 M), rt, 30 min. b) TrCl (1.5 equiv.), Et_3N (1.5 equiv.), DMAP (10 mol %), DMF (0.42 M), rt, N_2, 18 h. c) i: NaH (1.5 equiv.), DMF (0.3 M), ice-bath, N_2, 15 min. ii: MeI (1.5 equiv.), ice-bath, 14h. d) TFA (3 equiv.), DCM (0.24 M), ice-bath, 1h. e) TCCA (1.05 equiv.), TEMPO (1 mol %), DCM (0.5 M), ice-bath, 50 min.

Scheme S11: Preparation of D-Xylose aldehyde

First step (protection)[30]: To a solution of D-Xylose (30 mmol; 4.5 g) in acetone (150 mL, 0.2 M), H_2SO_4 98% (3 mL) was added. This reaction solution was stirred for 30 minutes at room temperature. After this time, the reaction solution was cooled in an ice bath and neutralized with the addition of $NaOH_{(aq)}$ (6 M). After that, the solution was filtered and the salt formed was washed with acetone (80 mL). The solvent was evaporated under reduced pressure and the aqueous phase was extracted with EtOAc (3 x 80 mL). The combined organic phases were washed with brine (1 x 50 mL), dried over anhydrous Na_2SO_4, filtered and concentrated in vacuo, yielding a yellowish oil in 70% (21 mmol, 4 g). This product was used in the next step without further purification.

Second step (tritylation)[31]: The product obtained in the previous step (21 mmol, 4 g) was solubilized in anhydrous DMF (50 mL; 0.42 M) and then Et_3N (31.5 mmol, 1.5 equiv.; 4.4 mL), TrCl (31.5 mmol, 1.5 equiv.; 8.78 g) and DMAP (2.1 mmol, 10 mol%, 257 mg) was added under nitrogen atmosphere. This reaction solution was kept under vigorous stirring at room temperature for 18 hours. At the end, cold water was added and the reaction solution was extracted with DCM (3 x 100 mL). The combined organic phases were washed with saturated solution of NH_4Cl (2 x 60 mL), brine (1 x 60 mL), dried over anhydrous Na_2SO_4, filtered and evaporated under reduced pressure, affording a white solid in 70% yield (17.7 mmol, 6.36 g). This product was used in the next step without further purification.

Third step (methylation): The product obtained in the previous step (14.7 mmol, 1 equiv.; 6.36 g) was solubilized in anhydrous DMF (50 mL; 0.3 M), under nitrogen atmosphere, and cooled in an ice bath. To this solution, 60% NaH in mineral oil (22 mmol, 1.5 equiv.; 882 mg) was added. The reaction solution was kept under stirring for 15 minutes and then MeI (22 mmol, 1.5 equiv.; 1.4 mL) was added dropwise. After that, the ice bath was removed and the reaction was kept at room temperature under stirring for 14 hours. At the end, cold water (50 mL) was added and the reaction solution was extracted with EtOAc (3 x 100 mL) and the combined organic phases were washed with brine (2 x 80 mL), dried over anhydrous Na_2SO_4, filtered and evaporated. under reduced pressure, providing a white solid in 65% yield (9.6 mmol, 4.27 g). This product was used in the next step without further purification.

Fourth step (detritylation)[30b]: The product obtained in the previous step (9.6 mmol, 1 equiv.; 4.27 g) was solubilized in DCM (40 mL; 0.24 M) and cooled in an ice bath. To this solution was added TFA (28.8 mmol, 3 equiv.; 2.2 mL). This reaction solution was kept under stirring for 60 minutes. At the end, it was neutralized with saturated $Na_2CO_{3(aq)}$, filtered and the solid washed with DCM (60 mL). The phases were separated in a separatory funnel and the aqueous phase was extracted with DCM (1 x 60 mL). The combined organic phases were washed with brine (1 x 60 mL), dried over anhydrous Na_2SO_4, filtered and evaporated under vacuum, affording a viscous oil in 85% yield (8.12 mmol, 1.66 g). This product was used in the next step without further purification.

Fifth step (oxidation)[32]: The product obtained in the previous step (8.12 mmol, 1 equiv.; 1.66 g) was solubilized in DCM (16 mL; 0.5 M) and this solution was cooled in an ice bath. TCCA (8.53 mmol, 1.05 equiv.; 2 g) was added to the solution followed by TEMPO (0.08 mmol, 1 mol%, 13 mg). After TEMPO addition, the ice bath was removed and the reaction solution was kept under vigorous stirring for 50 minutes. After, it was filtered under vacuum in a sintered plate funnel containing Celite and the solid washed with DCM (60 mL). The organic phase was washed with saturated $Na_2CO_{3(aq)}$ solution (2 x 30 mL), $HCl_{(aq)}$ 10% solution (2 x 30 mL) and brine (1 x 50 mL). The organic phase was dried over anhydrous Na_2SO_4, filtered and evaporated under reduced pressure, yielding a viscous oil in 83% (6.74 mmol; 1.36 g). This crude product was used in the next step without further purification. This product was used in the next step without further purification.

(3a*R*,5*S*,5a*R*,8a*S*,8b*R*)-2,2,7,7-tetramethyltetrahydro-3a*H*-bis([1,3]dioxolo)[4,5-*b*:4',5'-*d*]pyran-5-carbaldehyde

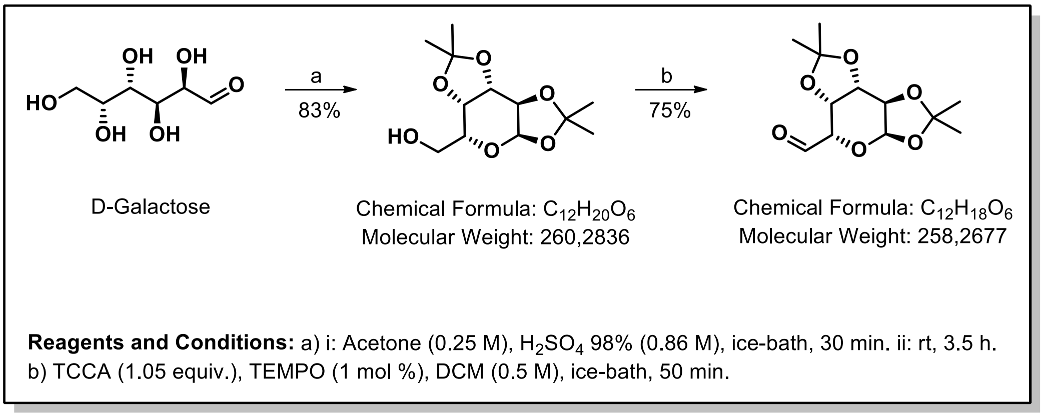

Scheme S12: Preparation of D-Galactose bis-acetonideo aldehyde

First step (protection)[33]: To a solution of D-Galactose (30 mmol; 5.4 g) in acetone (120 mL) cooled in an ice bath, H_2SO_4 (5.5 mL) was added. After 30 minutes, the ice bath was removed, and the reaction was maintained under vigorous stirring for 3.5 hours. After that, it was neutralized with $NaHCO_{3(s)}$, filtered and the solid washed with acetone (50 mL). The organic phase was dried over anhydrous Na_2SO_4, filtered and evaporated, providing a yellowish viscous oil in 83% yield (24.9 mmol, 6.48 g). This crude product was used in the next step without further purification.

Second step (oxidation)[32]: The crude product obtained in the previous step (24.9 mmol; 6.48 g) was treated as described above, affording the aldehyde as colorless viscous oil in 75% yield (18.7 mmol; 4.83 g). This crude product was used in the next step without further purification.

2.6 Nitrones (Nit.1 – Nit. 7)[34]

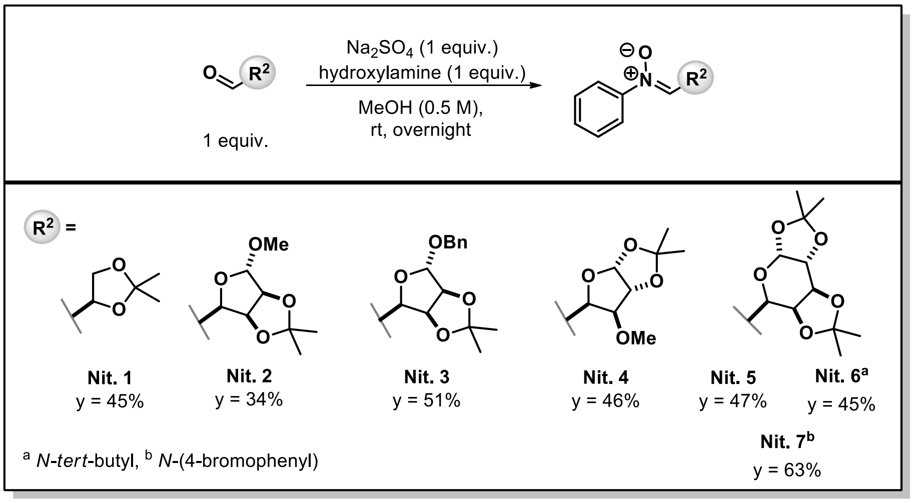

Scheme S13: Preparation of monosaccharides-derived nitrones

The aldehydes obtained from protected monosaccharides were subjected to condensation with *N*-phenylhydroxylamine (prepared and used on the same day). The condensation protocol is described below:

To a solution of aldehyde (7 mmol; 1 equiv.) in MeOH (15 mL; 0.5 M), anhydrous Na_2SO_4 (7 mmol; 1 equiv., ~1g) and hydroxylamine (7 mmol; 1 equiv.) was added. The reaction solution was kept under vigorous stirring for 16 hours. Afterwards, it was filtered and the solid washed with EtOAc. The solvent was evaporated under reduced pressure and the crude product was purified by flash column chromatography with yields from 34 to 63%.

Advertisement: For optimal results, it is advisable to prepare and use the N-phenyl glycosyl nitrones immediately. Small impurities have been observed when the nitrones are kept in solvent, making it difficult to obtain clean NMR spectra (e.g., compounds 3, 5, and 7)

2.6.1 Spectroscopic data of the compounds

(*S*,*Z*)-*N*-((2,2-dimethyl-1,3-dioxolan-4-yl)methylene)aniline oxide

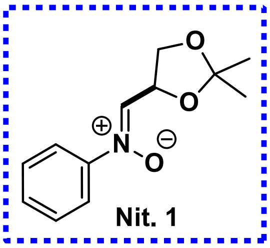

The product **Nit. 1** was obtained as a viscous yellow oil in 45% yield (0.7 g). The crude material was purified by flash column chromatography (DCM 100%; EtOAc 5% in DCM). R_f = 0.11 (100% DCM). The spectroscopic data can be found in literature[35]. HRMS (ESI) $[M + H]^+$ m/z: calculated for $C_{12}H_{15}NO_3H$ 222.1130, found: 222.1125.

(*Z*)-*N*-(((3a*S*,4*S*,6*S*,6a*S*)-6-methoxy-2,2-dimethyltetrahydrofuro[3,4-*d*][1,3]dioxol-4-yl)methylene)aniline oxide

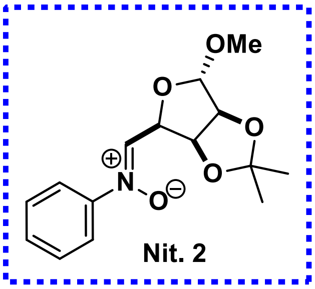

The product **Nit. 2** was obtained as a yellowish foam in 34% yield (0.7 g). The crude material was purified by flash column chromatography (EtOAc in Hexanes 20-40%). R_f = 0.44 (50% EtOAc in Hexanes). **^{1}H NMR (400 MHz, $CDCl_3$)** δ 7.76 – 7.68 (m, 2H), 7.47 – 7.40 (m, 3H), 5.29 – 5.20 (ddd, *J* = 17.0, 5.9, 2.8 Hz, 2H), 5.00 (s, 1H), 4.64 (d, *J* = 5.7 Hz, 1H), 3.37 (s, 3H), 1.43 (s, 3H), 1.30 (s, 3H) ppm. **^{13}C NMR (101 MHz, $CDCl_3$)** δ 135.6, 130.5, 129.2, 121.7, 112.9, 107.4, 84.7, 80.1, 54.9, 26.3, 24.8 ppm. **HRMS** (ESI) $[M + H]^+$ m/z: calculated for $C_{15}H_{19}NO_5H$ 294.1336 found: 294.1341.

(*Z*)-*N*-(((3a*S*,4*S*,6*S*,6a*S*)-6-(benzyloxy)-2,2-dimethyltetrahydrofuro[3,4-*d*][1,3]dioxol-4-yl)methylene)aniline oxide

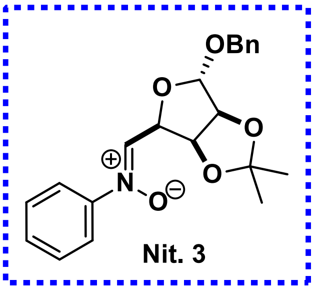

The product **Nit. 3** was obtained as a yellowish foam in 51% yield (1.32 g). The crude material was purified by flash column chromatography (EtOAc in Hexanes 20-40%). R_f = 0.36 (30% EtOAc in Hexanes). **HRMS** (ESI) $[M + H]^+$ m/z: calculated for $C_{21}H_{23}NO_5H$ 370.1649, found: 370.1643.

(*Z*)-*N*-(((3a*R*,5*S*,6*S*,6a*R*)-6-methoxy-2,2-dimethyltetrahydrofuro[2,3-*d*][1,3]dioxol-5-yl)methylene)aniline oxide

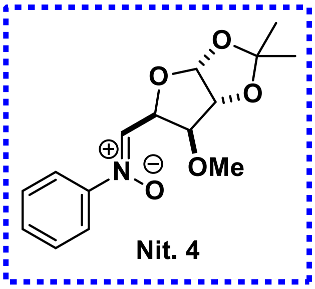

The product **Nit. 4** was obtained as an orange-brown foam in 46% yield (0.94 g). The crude material was purified by flash column chromatography (EtOAc in Hexanes 25-40%). R_f = 0.25 (50% EtOAc in Hexanes). **^{1}H NMR (400 MHz, CDCl$_3$)** δ 7.54 – 7.47 (m, 2H), 7.30 – 7.23 (m, 3H), 7.22 (d, J = 4.7 Hz, 1H), 5.81 (d, J = 3.7 Hz, 1H), 5.23 (dd, J = 4.7, 3.3 Hz, 1H), 4.45 (d, J = 3.7 Hz, 1H), 4.28 (d, J = 3.3 Hz, 1H), 3.20 (s, 3H), 1.34 (s, 3H), 1.16 (s, 3H) ppm. **^{13}C NMR (101 MHz, CDCl$_3$)** δ 146.8, 136.3, 130.6, 129.3, 121.5, 112.4, 105.2, 84.3, 82.3, 78.4, 58.5, 27.1, 26.5 ppm. **HRMS** (ESI) $[M+H]^+$ m/z: calculated for $C_{15}H_{19}NO_5H$ 294.1336; found: 294.1333.

(*Z*)-*N*-(((3a*R*,5*S*,5a*S*,8a*S*,8b*R*)-2,2,7,7-tetramethyltetrahydro-3a*H*-bis([1,3]dioxolo)[4,5-*b*:4',5'-*d*]pyran-5-yl)methylene)aniline oxide

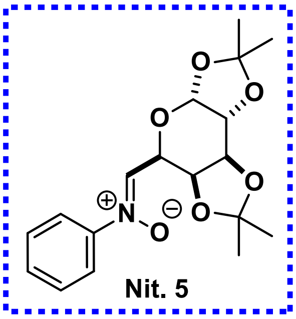

The product **Nit. 5** was obtained as a white foam in 47% yield (1.15 g). The crude material was purified by flash column chromatography (EtOAc in Hexanes 15-30%). R_f = 0.15 (30% EtOAc in Hexanes). **HRMS** (ESI) $[M + Na]^+$ m/z: calculated for $C_{18}H_{23}NO_6Na$ 372.1418, found: 372.1415.

(*Z*)-2-methyl-*N*-(((3a*R*,5*S*,5a*S*,8a*S*,8b*R*)-2,2,7,7-tetramethyltetrahydro-3a*H*-bis([1,3]dioxolo)[4,5-*b*:4',5'-*d*]pyran-5-yl)methylene)propan-2-amine oxide

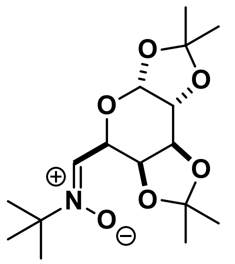

Nit. 6

The product **Nit. 6** was obtained as a white foam in 45% yield (1.04 g). The crude material was purified by flash column chromatography (EtOAc in Hexanes 10-20%). R_f = 0.2 (50% EtOAc in Hexanes). **^{1}H NMR (400 MHz, $CDCl_3$)** δ 6.65 (d, J = 4.9 Hz, 1H), 5.32 (d, J = 5.0 Hz, 1H), 4.80 (dd, J = 4.9, 2.0 Hz, 1H), 4.53 (dd, J = 7.9, 2.1 Hz, 1H), 4.40 (dd, J = 7.9, 2.5 Hz, 1H), 4.13 (dd, J = 5.0, 2.5 Hz, 1H), 1.35 (s, 3H), 1.28 (s, 9H), 1.21 (s, 3H), 1.10 (d, J = 13.7 Hz, 6H) ppm. **^{13}C NMR (101 MHz, $CDCl_3$)** δ 132.2, 108.8, 108.6, 95.8, 70.1, 70.0, 69.5, 69.2, 65.6, 27.5, 25.8, 25.7, 24.6, 24.0 ppm. **HRMS** (ESI) $[M + Na]^+$ m/z: calculated for $C_{16}H_{27}NO_6Na$ 352.1731; found: 352.1727.

(*Z*)-4-bromo-*N*-(((3a*R*,5*S*,5a*S*,8a*S*,8b*R*)-2,2,7,7-tetramethyltetrahydro-3a*H*-bis([1,3]dioxolo)[4,5-*b*:4',5'-*d*]pyran-5-yl)methylene)aniline oxide

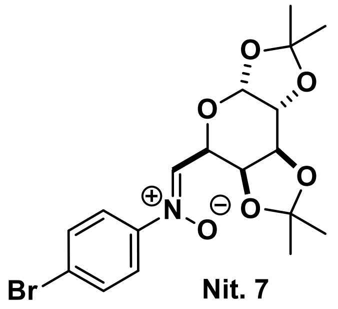

Nit. 7

The product **Nit. 7** was obtained as a yellowish foam in 63% yield (1.89 g). The crude material was purified by flash column chromatography (EtOAc in Hexanes 15-35%). R_f = 0.3 (40% EtOAc in Hexanes). **HRMS** (ESI) $[M + Na]^+$ m/z: calculated for $C_{18}H_{22}NO_6BrNa$ 450.0523, found: 450.0511.

2.7 3-exomethylene β-glycosyl β-lactam (2a – 2g)[36]

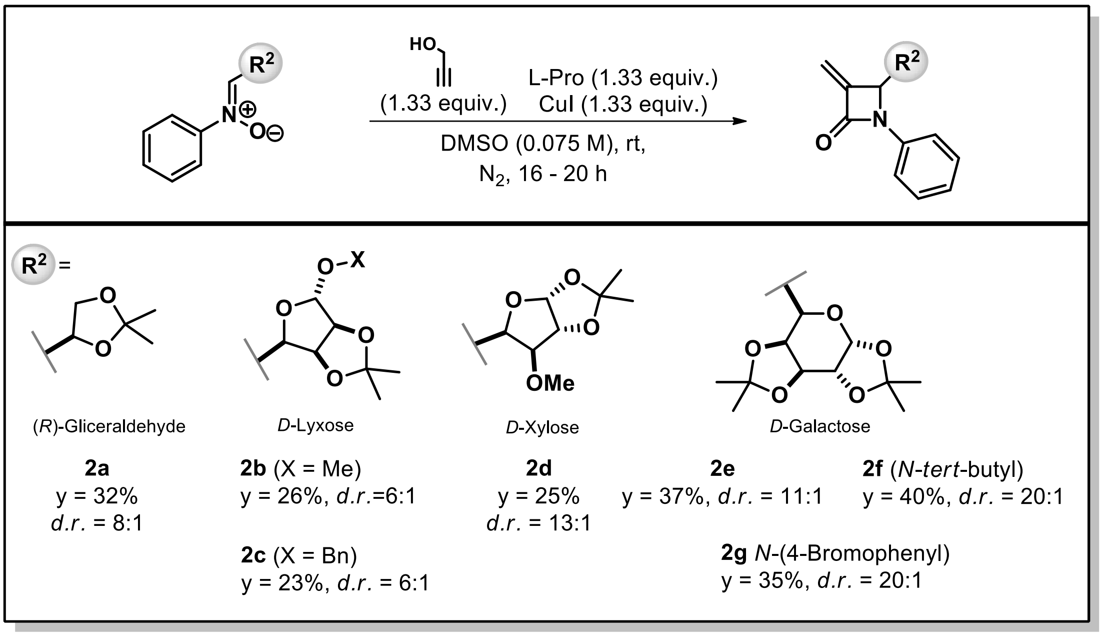

Scheme S14: Preparation and scope of 3-exomethylene β-glycosyl β-lactams exploited in the photoinduced reactions

In a 50 mL double-tubbed round bottom flask, previously flamed and cooled under an N_2 atmosphere, were added dry DMSO (20 mL), L-Proline (4 mmol, 1.33 equiv.; 460 mg) and propargylic alcohol (4 mmol, 1.33 equiv.; 0.233 mL) (Solution A). In another 25 mL round bottom flask, nitrone (3 mmol) and 15 mL of dry DMSO were added (Solution B). Both solutions were degassed by bubbling $N_{2(g)}$ for 20 minutes. (Degassing using freeze-pump-thaw cycles was also tested for the standard substrate and the result was similar). Then, CuI (4 mmol, 1.33 equiv.; 762 mg) was added to solution A where it remained under stirring for another 10 minutes. Next, solution B was transferred via cannula dropwise into solution A for 15 minutes. Then, the flask containing all components of the reaction solution was sealed with parafilm and kept under stirring for 16-20 hours. When finished, brine (50 mL) was added and extracted with AcOEt (3 x 100 mL). The organic phases were combined, dried over anhydrous Na_2SO_4, filtered and the solvent evaporated under reduced pressure. The product was isolated by flash column chromatography with yields between 23 and 40%.

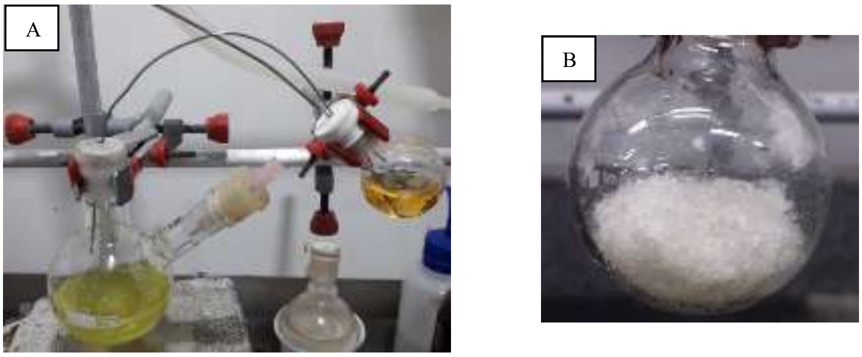

Figure S1: A) Kinugasa reaction during the addition of nitrone solution. B) Isolated product **2e**

2.7.1 Spectroscopic data of the compounds

(*S*)-4-((*S*)-2,2-dimethyl-1,3-dioxolan-4-yl)-3-methylene-1-phenylazetidin-2-one

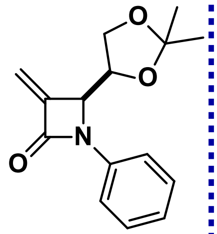

2a

The crude product was purified by flash column chromatography (EtOAc in Hexanes 5-25%). R_f = 0.42 (30% EtOAc in Hexanes), affording the product **2a** as a white solid impregnated with a viscous brownish oil. This product was washed with hexanes and filtrated, yielding a white solid with 32% yield (0.25 g) and *d.r.* 8:1. **^{1}H NMR (400 MHz, $CDCl_3$)** δ 7.57 – 7.51 (m, 2H), 7.36 – 7.29 (m, 2H), 7.14 – 7.03 (m, 1H), 5.87 (d, *J* = 1.8 Hz, 1H), 5.38 (t, *J* = 1.5 Hz, 1H), 4.82 – 4.78 (m, 1H), 4.53 (td, *J* = 6.7, 3.3 Hz, 1H), 4.05 (dd, *J* = 8.5, 6.5 Hz, 1H), 3.86 (dd, *J* = 8.6, 6.8 Hz, 1H), 1.33 (s, 3H), 1.28 (s, 3H) ppm. **^{13}C NMR (101 MHz, $CDCl_3$)** *δ* 160.8, 145.0, 137.7, 129.0, 124.6, 118.0, 111.9, 110.0, 75.5, 65.1, 60.7, 26.0, 25.1 ppm. **HRMS** (ESI) $[M + H]^+$ m/z: calculated for $C_{15}H_{17}NO_3H$ 260.1281, found: 260.1281.

(*S*)-4-((3a*S*,4*R*,6*S*,6a*S*)-6-methoxy-2,2-dimethyltetrahydrofuro[3,4-*d*][1,3]dioxol-4-yl)-3-methylene-1-phenylazetidin-2-one

The product **2b** was obtained as a viscous oil in 26% yield (0.26 g) and *d.r.* 6:1. The crude material was purified by flash column chromatography (EtOAc in Hexanes 10-20%). R_f = 0.64

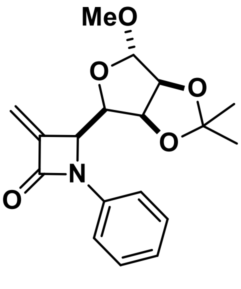

(30% EtOAc in Hexanes). (*) indicates the minor diastereoisomer when it is not superimposed. **^{1}H NMR (400 MHz, $CDCl_3$ diastereoisomeric mixture)** δ 7.80 (d, *J* = 8.2 Hz, 2H)*, 7.62 (d, *J* = 8.1 Hz, 2H), 7.33 (t, *J* = 7.9 Hz, 2H), 7.10 (t, *J* = 7.4 Hz, 1H), 5.94 (s, 1H)*, 5.92 (s, 1H), 5.67 (s, 1H)*, 5.59 (s, 1H), 5.01 (s, 1H)*, 4.94 (d, *J* = 7.2 Hz, 1H), 4.86 (s, 1H), 4.62 (dd, *J* = 5.7, 3.0 Hz, 1H), 4.57 (d, *J* = 6.1 Hz, 1H)*, 4.55 (d, *J* = 5.7 Hz, 1H), 4.03 (dd, *J* = 7.1, 3.1 Hz, 1H)*, 4.00 (dd, *J* = 7.2, 3.0 Hz, 1H), 3.24 (s, 3H), 3.16 (s, 3H)*, 1.54 (s, 3H)*, 1.50 (s, 3H), 1.38 (s, 1H)*, 1.35 (s, 3H) ppm. **^{13}C NMR (101 MHz, $CDCl_3$ diastereoisomeric mixture)** δ 161.0, 145.6, 137.7, 129.1, 128.9*, 124.4, 124.3*, 118.0, 117.9*, 113.1, 112.9*, 106.9, 85.7, 84.6*, 82.2*, 81.2*, 79.9, 61.9*, 57.6, 54.7*, 54.6, 26.6*, 26.2, 25.0*, 24.9 ppm. **HRMS** (ESI) $[M + H]^+$ m/z: calculated for $C_{18}H_{21}NO_5H$ 332.1492 found: 332.1487

(*S*)-4-((3a*S*,4*R*,6*S*,6a*S*)-6-(benzyloxy)-2,2-dimethyltetrahydrofuro[3,4-*d*][1,3]dioxol-4-yl)-3-methylene-1-phenylazetidin-2-one

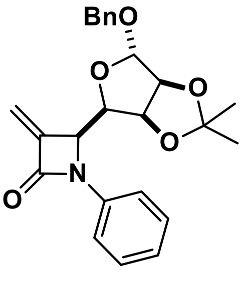

The product **2c** was obtained as light brownish oil in 23% yield (0.28 g) and *d.r.* 6:1. The crude material was purified by flash column chromatography (EtOAc in Hexanes 10-20%). R_f = 0.5 (30% EtOAc in Hexanes). (*) indicates the minor diastereoisomer when it is not superimposed. **^{1}H NMR (400 MHz, $CDCl_3$ diastereoisomeric mixture)** δ 7.74 (d, *J* = 7.7 Hz, 2H)*, 7.55 (d, *J* = 7.7 Hz, 2H), 7.37 – 7.11 (m, 8H), 7.16 – 7.02 (m, 8H)*, 5.59 (s, 1H)*, 5.86 (d, *J* = 0.6 Hz, 1H), 5.52 (s, 1H), 5.20 (s, 1H)*, 5.09 (s, 1H)*, 4.98 (s, 1H), 4.88 (d, *J* = 6.8 Hz, 1H), 4.73 (dd, *J* = 5.8, 3.9 Hz, 1H)*, 4.65 – 4.62 (m, 2H)*, 4.61 – 4.53 (m, 2H), 4.46 (d, *J* = 11.6 Hz, 1H), 4.33 (d, *J* = 11.6 Hz, 1H), 4.24 (d, *J* = 11.6 Hz, 1H)*, 4.04 (dd, *J* = 6.9, 2.2 Hz, 1H), 4.00 (dd, *J* = 8.6, 3.9 Hz, 1H)*, 1.46 (s, 3H)*, 1.43 (s, 3H), 1.27 (s, 3H), 1.22 (s, 3H)* ppm. **^{13}C NMR (101 MHz, $CDCl_3$ diastereoisomeric mixture)** δ 161.0, 145.5, 144.6*, 137.6, 137.1, 129.4*, 129.3*, 129.2, 128.9, 128.68*, 128.6, 128.5*, 128.4, 128.2, 128.0, 127.2*, 124.5, 124.4*, 118.1*, 118.0, 115.4*, 113.4*, 113.2, 112.9, 105.5*, 105.0, 84.6*, 82.5*, 81.2*, 79.9, 79.8, 69.1, 68.9*, 61.7*, 57.7, 26.6*, 26.1, 24.9*, 24.8 ppm. **HRMS** (ESI) $[M + H]^+$ m/z: calculated for $C_{24}H_{25}NO_5H$ 408.1805, found: 408.1811.

(*S*)-4-((3a*R*,5*R*,6*S*,6a*R*)-6-methoxy-2,2-dimethyltetrahydrofuro[2,3-*d*][1,3]dioxol-5-yl)-3-methylene-1-phenylazetidin-2-one

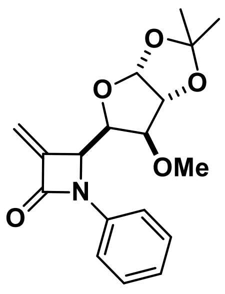

2d

The product **2d** was obtained as viscous brown oil in 25% yield (0.25 g) and *d.r.* 13:1. The crude material was purified by flash column chromatography (DCM in Hexanes 90%). R_f = 0.7 (50% EtOAc in Hexanes). (*) indicates the minor diastereoisomer when it is not superimposed. **^{1}H NMR (400 MHz, $CDCl_3$ diastereoisomeric mixture)** δ 7.72 (dd, *J* = 7.9, 1.1 Hz, 2H)*, 7.38 (dd, *J* = 8.6, 1.1 Hz, 2H), 7.30 – 7.25 (m, 2H), 7.07 – 7.02 (m, 1H), 6.03 (d, *J* = 3.9 Hz, 1H)*, 5.81 (t, *J* = 3.1 Hz, 2H), 5.50 (s, 1H)*, 5.48 (t, *J* = 1.0 Hz, 1H), 4.82 (dt, *J* = 8.4, 1.5 Hz, 1H)*, 4.80 (dt, *J* = 7.1, 1.2 Hz, 1H), 4.57 (d, *J* = 3.8 Hz, 1H), 4.17 (dd, *J* = 7.1, 3.2 Hz, 1H), 3.56 (d, *J* = 3.2 Hz, 1H), 3.46 (s, 3H)*, 3.39 (s, 3H), 1.40 (s, 3H)*, 1.35 (s, 3H), 1.32 (s, 3H)*, 1.22 (s, 3H) ppm. **^{13}C NMR (101 MHz, $CDCl_3$ diastereoisomeric mixture)** δ 161.0, 145.2, 144.7*, 138.2*, 137.2, 129.1, 128.9*, 124.5, 124.2*, 117.8, 113.6, 111.9, 105.7*, 104.4, 85.6*, 83.7, 82.6*, 80.9, 80.4, 80.0*, 57.0, 56.9, 26.7, 26.6*, 26.1, 26.19* ppm. **HRMS** (ESI) $[M + Na]^+$ m/z: calculated for $C_{18}H_{21}NO_5Na$ 354.1312; found: 354.1306.

(*S*)-3-methylene-1-phenyl-4-((3a*R*,5*R*,5a*S*,8a*S*,8b*R*)-2,2,7,7-tetramethyltetrahydro-3a*H*-bis([1,3]dioxolo)[4,5-*b*:4',5'-*d*]pyran-5-yl)azetidin-2-one

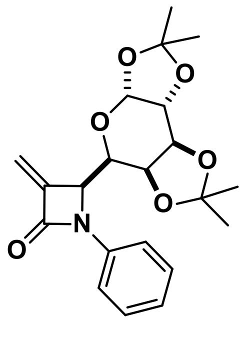

2e

The product **2e** was obtained as white foam in 37% yield (0.43 g) and *d.r.* 11:1. The crude material was purified by flash column chromatography (EtOAc in Hexanes 10-25%). R_f = 0.63 (40% EtOAc in Hexanes). (*) indicates the minor diastereoisomer when it is not superimposed. **^{1}H NMR (400 MHz, $CDCl_3$ diastereoisomeric mixture)** δ 7.75 (d, *J* = 8.3 Hz, 2H)*, 7.57 (d, *J* = 8.4 Hz, 2H), 7.33 (t, *J* = 7.7 Hz, 2H), 7.10 (t, *J* = 7.4 Hz, 1H), 5.95 (s, 1H)*, 5.90 (s, 1H), 5.61 (s, 1H), 5.60 (s, 0H)*, 5.51 (d, *J* = 4.9 Hz, 1H), 4.77 (d, *J* = 5.6 Hz, 1H), 4.55 (dd, *J* = 8.0, 1.4 Hz, 1H), 4.37 – 4.30 (m, 1H)*, 4.25 (dd, *J* = 4.5, 1.6 Hz, 1H), 4.20 (d, *J* = 8.1 Hz, 1H), 3.98 (d, *J* = 6.1 Hz, 1H), 3.95 (d, *J* = 6.1 Hz, 1H)*, 1.53 (s, 3H)*, 1.49 (s, 3H), 1.38 (s, 3H)*, 1.34 (s, 3H), 1.30 (s, 3H)*, 1.28 (s, 3H), 1.26 (s, 3H) ppm. **^{13}C NMR (101 MHz, $CDCl_3$ $CDCl_3$ diastereoisomeric mixture)** δ 161.4, 145.1, 137.5, 129.1, 128.8, 128.5*, 124.4, 118.4*, 118.1, 113.4, 113.3*, 109.5, 108.8, 96.3*, 96.2, 72.1*, 71.3, 70.8, 70.6,

70.2*, 68.0, 63.1*, 59.2, 26.1*, 26.0, 25.9, 24.6*, 24.9, 23.9 ppm. **HRMS** (ESI) [M + Na]$^+$ m/z: calculated for $C_{21}H_{25}NO_6Na$ 410.1574; found: 410.1566.

(*S*)-1-(*tert*-butyl)-3-methylene-4-((3a*R*,5*R*,5a*S*,8a*S*,8b*R*)-2,2,7,7-tetramethyltetrahydro-3a*H*-bis([1,3]dioxolo)[4,5-*b*:4',5'-*d*]pyran-5-yl)azetidin-2-one

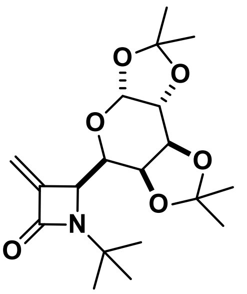

2f

The product **2f** was obtained as white foam in 40% yield (0.44 g) and *d.r.* 20:1. The crude material was purified by flash column chromatography (EtOAc in Hexanes 10-50%). R_f = 0.43 (50% EtOAc in Hexanes). **^{1}H NMR (400 MHz, $CDCl_3$)** δ 5.64 (d, *J* = 1.3 Hz, 1H), 5.52 (d, *J* = 5.3 Hz, 1H), 5.35 (d, *J* = 0.5 Hz, 1H), 4.57 (dd, *J* = 8.1, 2.0 Hz, 1H), 4.32 (s, 1H), 4.29 – 4.22 (m, 2H), 4.06 (s, 1H), 1.47 (s, 3H), 1.41 (s, 3H), 1.39 (s, 9H), 1.30 (s, 3H), 1.28 (s, 3H) ppm. **^{13}C NMR (101 MHz, $CDCl_3$)** δ 164.0, 144.2, 110.3, 109.1, 107.9, 96.5, 71.4, 70.9, 70.1, 66.3, 60.9, 54.0, 28.3, 25.9, 25.6, 24.5, 23.7 ppm. **HRMS** (ESI) [M + Na]$^+$ m/z: calculated for $C_{19}H_{29}NO_6Na$ 390.1887; found: 390.1885

(*S*)-1-(4-bromophenyl)-3-methylene-4-((3a*R*,5*R*,5a*S*,8a*S*,8b*R*)-2,2,7,7-tetramethyltetrahydro-3a*H*-bis([1,3]dioxolo)[4,5-*b*:4',5'-*d*]pyran-5-yl)azetidin-2-one

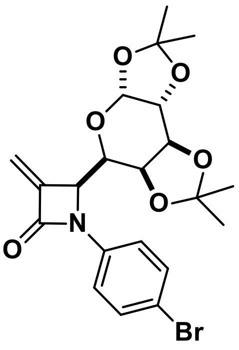

2g

The crude material was purified by flash column chromatography (Hexanes/DCM/EtOAc – 53/42/5 - 50/42/8 - 46/42/12 - 42/42/16). R_f = 0.37 (30% EtOAc in Hexanes). The product **2g** was obtained as white solid impregnated with a yellow oil. The isolated product was washed with hexanes and filtrated, yielding a white solid in 35% yield (0.49 g) and *d.r.* 20:1. **^{1}H NMR (400 MHz, $CDCl_3$)** δ 7.51 – 7.46 (m, 2H), 7.46 – 7.40 (m, 2H), 5.91 (s, 1H), 5.60 (s, 1H), 5.50 (d, *J* = 4.8 Hz, 1H), 4.74 (d, *J* = 6.2 Hz, 1H), 4.57 (dd, *J* = 8.1, 2.2 Hz, 1H), 4.25 (dd, *J* = 4.9, 2.2 Hz, 1H), 4.16 (dd, *J* = 8.0, 1.6 Hz, 1H), 3.92 (dd, *J* = 6.3, 1.5 Hz, 1H), 1.46 (s, 3H), 1.33 (d, *J* = 4.3 Hz, 6H), 1.27 (s, 3H) ppm. **^{13}C NMR (101 MHz, $CDCl_3$)** δ 161.3, 145.1, 136.8, 132.1, 119.7, 117.1, 113.8, 109.7, 108.9, 96.2, 71.3, 70.9, 70.7, 68.3, 59.5, 26.1, 25.9, 25.0, 24.1 ppm. **HRMS** (ESI) [M + Na]$^+$ m/z: calculated for $C_{21}H_{24}NO_6BrNa$ 488.0685; found: 488.0679.

2.8 Photoinduced reaction via EDA complex (3a – 3n; 4a – 4e)

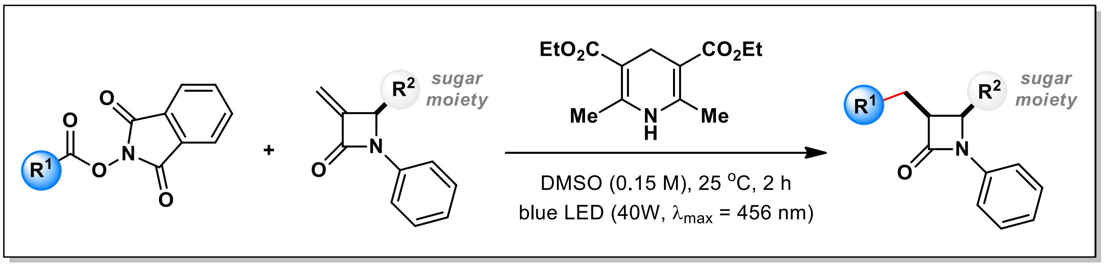

Scheme S15: Photoinduced diastereoselective functionalization of 3-exomethylene β-glycosyl β-lactams

In a Schlenk flask were added ***N*-(acyloxy)phthalimide** (0.3 mmol, 1.5 equiv.), **HE** (0.3 mmol, 1.5 equiv., 76 mg), **2e** (0.2 mmol, 1 equiv.; 77.5 mg) and DMSO (1.3 mL; 0.15M). This reaction solution was placed in front (4 cm away) of a blue LED (40W) and kept under stirring for 2 hours. At the end, the reaction solution was poured into a separation funnel, EtOAc (80 mL) was added, the organic phase was washed with $NaOH_{(aq)}$ (3 x 30 mL), $HCl_{(aq)}$ 10% (3 x 30 mL) and brine (1 x 30 mL). The organic phase was dried over anhydrous Na_2SO_4, filtered and concentrated in vacuo. The products were isolated by flash column chromatography with yields from 37% to 70% and *d.r.* from 5 to 20:1.

2.8.1 Spectroscopic data of compounds

(3*S*,4*S*)-3-((*Z*)-octadec-9-en-1-yl)-1-phenyl-4-((3a*R*,5*R*,5a*S*,8a*S*,8b*R*)-2,2,7,7-tetramethyltetrahydro-3a*H*-bis([1,3]dioxolo)[4,5-*b*:4',5'-*d*]pyran-5-yl)azetidin-2-one

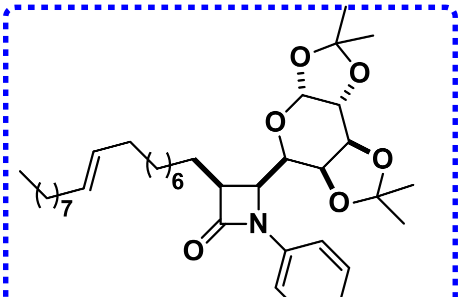

3a

The product **3a** was obtained as a viscous colorless oil in 50% yield (62.5 mg) and *d.r.* >20:1. The crude material was purified by flash column chromatography (EtOAc in Hexanes 5-10%). **^{1}H NMR (400 MHz, $CDCl_3$)** δ 7.58 (d, *J* = 8.0 Hz, 2H), 7.30 (t, *J* = 7.8 Hz, 2H), 7.09 (t, *J* = 7.4 Hz, 1H), 5.53 (d, *J* = 5.0 Hz, 1H), 5.40 – 5.30 (m, 2H), 4.47 (dd, *J* = 9.3, 6.2 Hz, 2H), 4.22 (dd, *J* = 5.0, 1.9 Hz, 1H), 3.99 (d, *J* = 8.1 Hz, 1H), 3.87 (d, *J* = 9.5 Hz, 1H), 3.39 (dt, *J* = 10.1, 5.7 Hz, 1H), 2.07 – 1.95 (m, 5H), 1.80 – 1.62 (m, 3H), 1.52 (d, *J* = 12.1 Hz, 7H), 1.34 – 1.25 (m, 27H), 0.88 (t, *J* = 6.7 Hz, 3H) ppm. **^{13}C NMR (101 MHz, $CDCl_3$)** δ 170.0, 137.6, 130.0, 128.8, 124.5, 119.3, 109.0, 108.5, 96.3, 67.7, 53.3, 52.8, 32.3, 32.0, 29.9, 29.8, 29.7, 29.66,

29.55, 29.5, 29.3, 28.5, 27.4, 26.2, 26.1, 25.4, 24.9, 23.7, 22.8, 14.2 ppm. **HRMS** (ESI) [M + H]$^+$ m/z: calculated for $C_{38}H_{58}NO_6H$ 626.4415; found: 626.4421.

(3*S*,4*S*)-3-(11-(benzylthio)undecyl)-1-phenyl-4-((3a*R*,5*R*,5a*S*,8a*S*,8b*R*)-2,2,7,7-tetramethyltetrahydro-3a*H*-bis([1,3]dioxolo)[4,5-*b*:4',5'-*d*]pyran-5-yl)azetidin-2-one

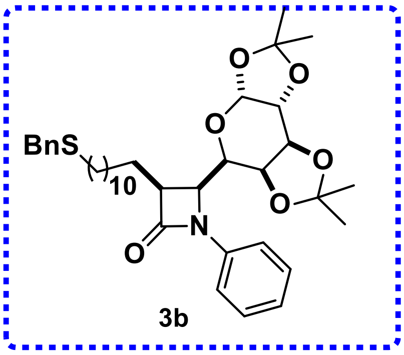

3b

The product **3b** was obtained as yellowish viscous oil in 46% yield (60 mg) and *d.r.* 16:1. The crude material was purified by flash column chromatography (DCM in Hexanes 50%). R_f = 0.55 (50% DCM in Hexanes). **^{1}H NMR (400 MHz, CDCl$_3$)** δ 7.52 (d, *J* = 7.6 Hz, 2H), 7.26 – 7.19 (m, 6H), 7.18 – 7.12 (m, 1H), 7.02 (t, *J* = 7.4 Hz, 1H), 5.45 (d, *J* = 5.0 Hz, 1H), 4.46 – 4.34 (m, 2H), 4.14 (dd, *J* = 5.0, 1.8 Hz, 1H), 3.91 (dd, *J* = 8.1, 1.0 Hz, 1H), 3.80 (d, *J* = 9.5 Hz, 1H), 3.62 (s, 2H), 3.30 (dt, *J* = 10.3, 5.7 Hz, 1H), 2.32 (t, *J* = 7.4 Hz, 2H), 1.99 – 1.89 (m, 2H), 1.75 – 1.65 (m, 1H), 1.64 – 1.56 (m, 1H), 1.55 – 1.40 (m, 9H), 1.25 – 1.15 (m, 20H) ppm. **^{13}C NMR (101 MHz, CDCl$_3$)** δ 169.9, 138.8, 137.6, 128.9, 128.7, 128.5, 126.9, 124.5, 119.2, 109.0, 108.4, 96.2, 70.9, 70.8, 70.6, 67.6, 53.2, 52.7, 36.3, 32.0, 31.4, 29.8, 29.74, 29.7, 29.67, 29.62, 29.6, 29.54, 29.5, 29.44, 29.4, 29.34, 29.31, 29.3, 29.0, 28.95, 28.4, 26.1, 26.0, 25.3, 24.8, 23.6, 22.8, 14.2 ppm. **HRMS** (ESI) [M + Na]$^+$ m/z: calculated for $C_{38}H_{53}NO_6SNa$ 674.3491; found: 674.3481.

(3*S*,4*S*)-3-((*R*)-4-((3*R*,5*R*,8*R*,9*S*,10*S*,13*R*,14*S*,17*R*)-3-hydroxy-10,13-dimethylhexadecahydro-1*H*-cyclopenta[*a*]phenanthren-17-yl)pentyl)-1-phenyl-4-((3a*R*,5*R*,5a*S*,8a*S*,8b*R*)-2,2,7,7-tetramethyltetrahydro-3a*H*-bis([1,3]dioxolo)[4,5-*b*:4',5'-*d*]pyran-5-yl)azetidin-2-one

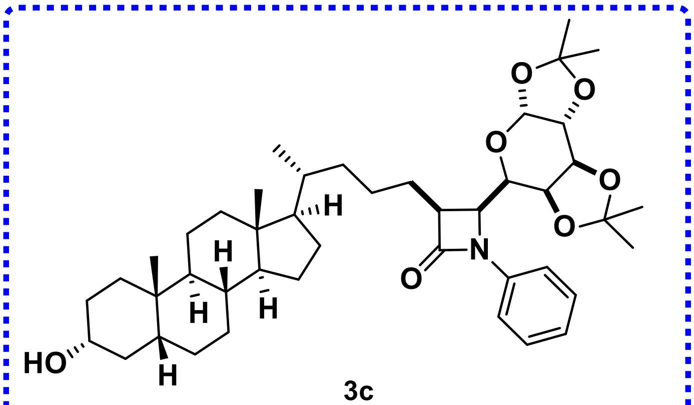

3c

The product **3c** was obtained as a viscous colorless oil in 45% yield (64.8 mg) and *d.r.* > 20:1. The crude material was purified by flash column chromatography (EtOAc in Hexanes 15%). **^{1}H NMR (400 MHz, CDCl$_3$)** δ 7.63 – 7.56 (m, 2H), 7.30 (t, *J* = 7.9

Hz, 2H), 7.09 (t, J = 7.4 Hz, 1H), 5.52 (d, J = 5.0 Hz, 1H), 4.57 – 4.39 (m, 2H), 4.22 (dd, J = 5.0, 1.9 Hz, 1H), 3.99 (dd, J = 8.1, 1.4 Hz, 1H), 3.86 (dd, J = 9.5, 1.3 Hz, 1H), 3.62 (tt, J = 10.8, 4.6 Hz, 1H), 3.39 (dt, J = 9.6, 5.8 Hz, 1H), 2.01 – 1.60 (m, 11H), 1.59 – 1.49 (m, 8H), 1.48 – 1.34 (m, 8H), 1.33 – 1.24 (m, 8H), 1.23 – 1.08 (m, 8H), 1.04 – 0.87 (m, 8H), 0.64 (s, 3H) ppm. **^{13}C NMR (101 MHz, CDCl$_3$)** δ 169.9, 137.6, 128.7, 124.5, 119.2, 109.0, 108.4, 96.3, 72.0, 70.9, 70.8, 70.6, 67.6, 56.6, 56.4, 53.3, 52.8, 42.8, 42.2, 40.5, 40.3, 36.5, 35.9, 35.7, 35.4, 34.7, 30.7, 28.4, 27.3, 26.5, 26.2, 26.0, 25.6, 25.1, 24.8, 24.4, 23.7, 23.5, 20.9, 18.7, 12.1 ppm. **HRMS** (ESI) $[M + Na]^+$ m/z: calculated for $C_{44}H_{65}NO_7Na$ 742.4653; found: 742.4653.

(3*S*,4*S*)-3-(cyclobutylmethyl)-1-phenyl-4-((3a*R*,5*R*,5a*S*,8a*S*,8b*R*)-2,2,7,7-tetramethyltetrahydro-3a*H*-bis([1,3]dioxolo)[4,5-*b*:4',5'-*d*]pyran-5-yl)azetidin-2-one

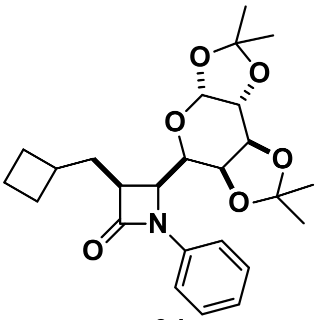

3d

The product **3d** was obtained as a white foam in 54% yield (47.9 mg) and *d.r.* = 20:1. The crude material was purified by flash column chromatography (DCM in Hexanes 50%). **^{1}H NMR (400 MHz, CDCl$_3$)** δ 7.51 (dd, J = 8.6, 1.0 Hz, 2H), 7.22 (t, J = 8.0 Hz, 2H), 7.02 (t, J = 7.4 Hz, 1H), 5.47 (d, J = 5.0 Hz, 1H), 4.42 – 4.34 (m, 2H), 4.15 (dd, J = 5.0, 1.9 Hz, 1H), 3.91 (dd, J = 8.1, 1.2 Hz, 1H), 3.79 (d, J = 9.5 Hz, 1H), 3.23 (dt, J = 10.3, 5.6 Hz, 1H), 2.70 – 2.58 (m, 1H), 2.11 – 2.00 (m, 3H), 1.87 – 1.72 (m, 3H), 1.65 – 1.49 (m, 2H), 1.46 (s, 3H), 1.43 (s, 3H), 1.24 (s, 3H), 1.19 (s, 3H) ppm. **^{13}C NMR (101 MHz, CDCl$_3$)** δ 169.8, 137.5, 128.7, 124.5, 119.24, 119.22, 109.0, 108.5, 96.2, 70.9, 70.8, 70.6, 67.5, 53.1, 50.4, 34.1, 32.2, 28.4, 28.0, 26.2, 26.0, 24.8, 23.6, 18.3 ppm. **HRMS** (ESI) $[M + Na]^+$ m/z: calculated for $C_{25}H_{33}NO_6Na$ 466.2200; found: 466.2190.

(3*S*,4*S*)-1-phenyl-3-((1-(phenylsulfonyl)piperidin-4-yl)methyl)-4-((3a*R*,5*R*,5a*S*,8a*S*,8b*R*)-2,2,7,7-tetramethyltetrahydro-3a*H*-bis([1,3]dioxolo)[4,5-*b*:4',5'-*d*]pyran-5-yl)azetidin-2-one

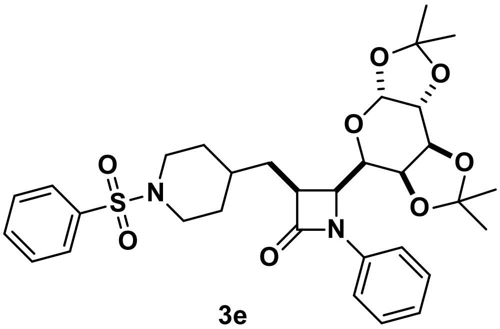

3e

The product **3e** was obtained as a white foam in 66% yield (331.3 mg; 0.82 mmol scale) and *d.r.* > 20:1. The crude material was purified by flash

column chromatography (EtOAc in Hexanes 10 – 20 %). **^{1}H NMR (400 MHz, CDCl$_3$)** δ 7.77 – 7.74 (m, 2H), 7.61 – 7.52 (m, 5H), 7.29 (t, *J* = 7.7 Hz, 2H), 7.12 – 7.07 (m, 1H), 5.48 (d, *J* = 5.0 Hz, 1H), 4.47 – 4.42 (m, 2H), 4.21 (dt, *J* = 5.1, 1.4 Hz, 1H), 3.96 (dd, *J* = 8.1, 1.4 Hz, 1H), 3.80 (dd, *J* = 10.7, 7.4 Hz, 3H), 3.44 (dt, *J* = 9.6, 5.7 Hz, 1H), 2.31 – 2.25 (m, 2H), 1.95 – 1.56 (m, 7H), 1.52 (s, 3H), 1.45 (s, 3H), 1.29 (s, 3H), 1.26 (s, 3H) ppm. **^{13}C NMR (101 MHz, CDCl$_3$)** δ 169.3, 137.3, 136.2, 132.8, 129.1, 128.8, 127.8, 124.7, 119.2, 109.1, 108.5, 96.2, 70.8, 70.7, 70.5, 67.5, 53.1, 49.5, 46.5, 33.4, 32.1, 31.1, 30.8, 26.2, 26.0, 24.8, 23.6 ppm. **HRMS** (ESI) [M + Na]$^+$ m/z: calculated for $C_{32}H_{40}N_2O_8SNa$ 635.2398; found: 635.2385.

(3*S*,4*S*)-1-(4-bromophenyl)-3-((1-(phenylsulfonyl)piperidin-4-yl)methyl)-4-((3a*R*,5*R*,5a*S*,8a*S*,8b*R*)-2,2,7,7-tetramethyltetrahydro-3a*H*-bis([1,3]dioxolo)[4,5-*b*:4',5'-*d*]pyran-5-yl)azetidin-2-one

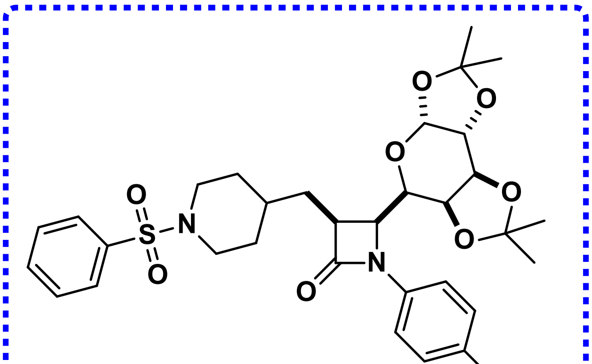

3f

The product **3f** was obtained as yellowish foam in 66% yield (523.8 mg; 1.15 mmol scale) and *d.r.* > 20:1. The crude material was purified by flash column chromatography (DCM in Hexanes 75 – 100 %; EtOAc in DCM 5 %). **^{1}H NMR (400 MHz, CDCl$_3$** δ 7.78 – 7.75 (m, 2H), 7.62 – 7.58 (m, 1H), 7.55 – 7.51 (m, 2H), 7.48 – 7.45 (m, 2H), 7.42 – 7.38 (m, 2H), 7.27 (s, 1H), 5.48 (d, *J* = 5.0 Hz, 1H), 4.49 (dd, *J* = 8.1, 2.0 Hz, 1H), 4.41 (dd, *J* = 9.5, 5.8 Hz, 1H), 4.23 (dd, *J* = 5.1, 1.9 Hz, 1H), 3.94 (dd, *J* = 8.1, 1.4 Hz, 1H), 3.79 (dd, *J* = 9.6, 1.4 Hz, 3H), 3.45 (dt, *J* = 10.2, 5.7 Hz, 1H), 2.32 – 2.25 (m, 2H), 1.99 – 1.88 (m, 2H), 1.84 – 1.78 (m, 1H), 1.72 – 1.65 (m, 1H), 1.63 – 1.55 (m, 2H), 1.51 (s, 3H), 1.47 (s, 3H), 1.30 (s, 3H), 1.29 (s, 3H) ppm. **^{13}C NMR (101 MHz, CDCl$_3$)** δ 169.2, 136.4, 136.2, 132.7, 131.7, 129.0, 127.8, 120.6, 117.4, 109.2, 108.5, 96.2, 70.8, 70.6, 70.6, 67.4, 53.3, 49.8, 46.4, 33.4, 32.1, 31.0, 30.8, 26.1, 26.0, 24.8, 23.8 ppm. **HRMS** (ESI) [M + Na]$^+$ m/z: calculated for $C_{32}H_{39}N_2O_8SBrNa$ 713.1503; found: 713.1511.

(3*S*,4*S*)-3-((1-methylcyclohexyl)methyl)-1-phenyl-4-((3a*R*,5*R*,5a*S*,8a*S*,8b*R*)-2,2,7,7-tetramethyltetrahydro-3a*H*-bis([1,3]dioxolo)[4,5-*b*:4',5'-*d*]pyran-5-yl)azetidin-2-one

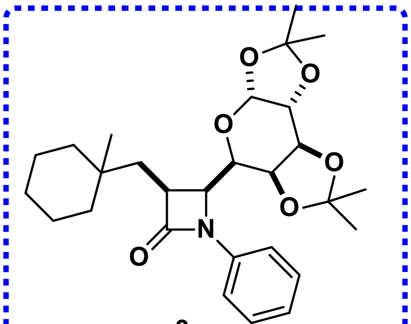

The product **3g** was obtained as a viscous colorless oil in 55% yield (53.4 mg) and *d.r.* > 20:1. The crude material was purified by flash column chromatography (EtOAc in Hexanes 10 – 20 %. **^{1}H NMR (400 MHz, CDCl$_3$)** δ 7.59 (d, *J* = 8.0 Hz, 2H), 7.29 (t, *J* = 7.8 Hz, 2H), 7.09 (t, *J* = 7.4 Hz, 1H), 5.54 (d, *J* = 5.1 Hz, 1H), 4.45 (d, *J* = 8.2 Hz, 1H), 4.41 (dd, *J* = 9.5, 5.8 Hz, 1H), 4.20 (dd, *J* = 5.0, 1.6 Hz, 1H), 3.97 (d, *J* = 8.2 Hz, 1H), 3.85 (d, *J* = 9.4 Hz, 1H), 3.54 – 3.46 (m, 1H), 2.28 (dd, *J* = 14.4, 2.5 Hz, 1H), 1.66 – 1.49 (m, 9H), 1.48 – 1.29 (m, 12H), 1.23 (s, 3H), 0.99 (s, 3H) ppm. **^{13}C NMR (101 MHz, CDCl$_3$)** δ 170.2, 137.6, 128.6, 124.4, 119.3, 108.9, 108.5, 96.2, 71.0, 70.7, 70.6, 67.6, 53.2, 48.3, 38.0, 37.7, 35.1, 32.8, 26.5, 26.2, 26.0, 24.8, 23.6, 22.1, 22.1 ppm. **HRMS** (ESI) [M + Na]$^+$ m/z: calculated for $C_{28}H_{39}NO_6Na$ 508.2670; found: 508.2667.

(3*S*,4*S*)-3-(adamantan-1-ylmethyl)-1-phenyl-4-((3a*R*,5*R*,5a*S*,8a*S*,8b*R*)-2,2,7,7-tetramethyltetrahydro-3a*H*-bis([1,3]dioxolo)[4,5-*b*:4',5'-*d*]pyran-5-yl)azetidin-2-one

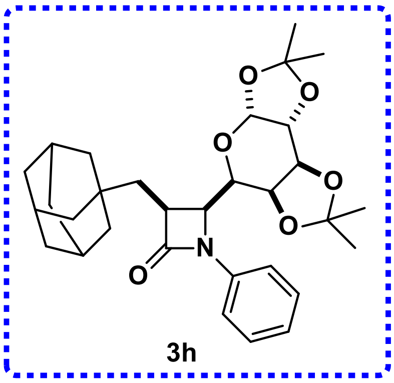

The product **3h** was obtained as yellowish foam in 62% yield (64.9 mg) and *d.r.* > 20:1. The crude material was purified by flash column chromatography (isocratic – Hexanes/DCM/EtOAc 6:2.5:1.5). **^{1}H NMR (400 MHz, CDCl$_3$)** δ 7.59 (d, *J* = 8.0 Hz, 2H), 7.29 (t, *J* = 7.8 Hz, 2H), 7.09 (t, *J* = 7.4 Hz, 1H), 5.54 (d, *J* = 4.9 Hz, 1H), 4.45 (dd, *J* = 8.1, 1.8 Hz, 1H), 4.40 (dd, *J* = 9.4, 5.8 Hz, 1H), 4.21 (dd, *J* = 5.0, 1.8 Hz, 1H), 3.97 (d, *J* = 8.1 Hz, 1H), 3.84 (d, *J* = 9.4 Hz, 1H), 3.55 (ddd, *J* = 10.0, 5.8, 2.4 Hz, 1H), 2.13 (dd, *J* = 14.3, 2.5 Hz, 1H), 1.99 – 1.98 (m, 3H), 1.73 – 1.56 (m, 13H), 1.53 (s, 3H), 1.51 (s, 3H), 1.32 (s, 3H), 1.24 (s, 3H) ppm. **^{13}C NMR (101 MHz, CDCl$_3$)** δ 170.3, 137.6, 128.6, 124.5, 119.4, 109.0, 108.6, 96.2, 71.0, 70.7, 70.6, 67.6, 53.1, 47.5, 42.4, 37.7, 37.1, 32.2, 28.8, 26.3, 26.1, 26.1, 24.9, 23.6 ppm. **HRMS** (ESI) [M + Na]$^+$ m/z: calculated for $C_{31}H_{41}NO_6Na$ 546.2826; found: 546.2809.

(3*S*,4*S*)-3-(5-(2,5-dimethylphenoxy)-2,2-dimethylpentyl)-1-phenyl-4-((3a*R*,5*R*,5a*S*,8a*S*,8b*R*)-2,2,7,7-tetramethyltetrahydro-3a*H*-bis([1,3]dioxolo)[4,5-*b*:4',5'-*d*]pyran-5-yl)azetidin-2-one

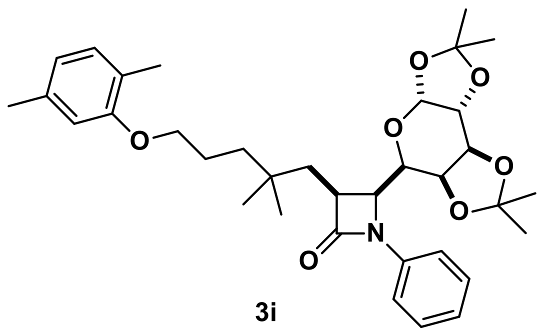

3i

The product **3i** was obtained as a viscous brownish oil in 52% yield (61.7 mg) and *d.r.* > 20:1. The crude material was purified by flash column chromatography (isocratic – DCM in Hexanes 80 %). **^{1}H NMR (400 MHz, CDCl$_3$)** δ 7.64 – 7.60 (m, 2H), 7.34 – 7.29 (m, 2H), 7.14 – 7.09 (m, 1H), 7.02 (d, *J* = 7.7 Hz, 1H), 6.67 (d, *J* = 5.8 Hz, 2H), 5.56 (d, *J* = 4.9 Hz, 1H), 4.49 – 4.42 (m, 2H), 4.23 (dt, *J* = 5.0, 1.5 Hz, 1H), 4.01 – 3.93 (m, 3H), 3.86 (d, *J* = 9.4 Hz, 1H), 3.51 (ddd, *J* = 9.2, 5.8, 2.4 Hz, 1H), 2.34 (s, 4H), 2.21 (s, 3H), 1.90 – 1.79 (m, 2H), 1.66 (dd, *J* = 14.2, 9.5 Hz, 1H), 1.56 (s, 3H), 1.50 (d, *J* = 12.5 Hz, 5H), 1.32 (s, 3H), 1.26 (s, 3H), 1.05 (d, *J* = 5.5 Hz, 6H) ppm. **^{13}C NMR (101 MHz, CDCl$_3$)** δ 170.0, 157.2, 137.6, 136.5, 130.3, 128.6, 124.5, 123.6, 120.6, 119.4, 112.1, 109.0, 108.5, 96.2, 70.9, 70.7, 70.6, 68.8, 67.6, 53.1, 48.8, 38.4, 34.9, 32.7, 27.3, 27.1, 26.2, 26.0, 24.8, 24.4, 23.6, 21.5, 15.9 ppm. **HRMS** (ESI) [M + Na]$^+$ m/z: calculated for $C_{35}H_{47}NO_7Na$ 616.3245; found: 616.3238.

(3*S*,4*S*)-3-(2-((2,6-dichlorophenyl)amino)phenethyl)-1-phenyl-4-((3a*R*,5*R*,5a*S*,8a*S*,8b*R*)-2,2,7,7-tetramethyltetrahydro-3a*H*-bis([1,3]dioxolo)[4,5-*b*:4',5'-*d*]pyran-5-yl)azetidin-2-one

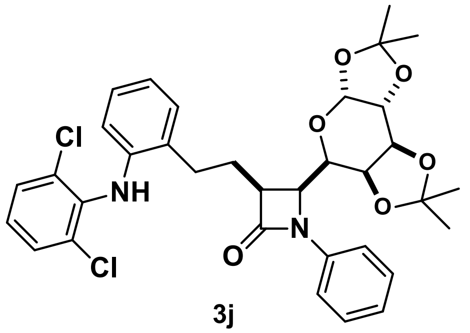

3j

The product **3j** was obtained as a white foam in 51% yield (65.1 mg) and *d.r.* = 12:1. The crude material was purified by flash column chromatography (gradient – DCM in Hexanes 20 – 60 %). **^{1}H NMR (400 MHz, CDCl$_3$)** δ 7.51 (dd, *J* = 8.5, 0.9 Hz, 2H), 7.31 – 7.27 (m, 2H), 7.25 – 7.16 (m, 3H), 7.02 (t, *J* = 7.4 Hz, 1H), 6.99 – 6.92 (m, 2H), 6.78 (td, *J* = 7.4, 1.1 Hz, 1H), 6.27 (dd, *J* = 8.0, 0.8 Hz, 1H), 6.11 (s, 1H), 5.51 (d, *J* = 5.0 Hz, 1H), 4.49 (dd, *J* = 9.5, 5.7 Hz, 1H), 4.39 (dd, *J* = 8.1, 2.0 Hz, 1H), 4.16 (dd, *J* = 5.1, 2.0 Hz, 1H), 3.92 (dd, *J* = 8.1, 1.2 Hz, 1H), 3.85 (d, *J* = 9.5 Hz, 1H), 3.50 (dt, *J* = 8.9, 6.9 Hz, 1H), 3.09 – 2.92 (m, 2H),

2.47 – 2.34 (m, 1H), 2.20 – 2.08 (m, 1H), 1.36 (s, 3H), 1.35 (s, 3H), 1.19 (s, 3H), 1.17 (s, 3H) ppm. **^{13}C NMR (101 MHz, CDCl$_3$)** δ 169.8, 141.9, 137.3, 132.0, 130.1, 128.7, 128.6, 128.2, 126.6, 125.2, 124.5, 120.4, 119.1, 114.6, 108.9, 108.5, 96.2, 70.8, 70.5, 70.4, 67.6, 53.6, 51.4, 29.2, 25.9, 25.7, 25.1, 24.6, 23.6 ppm. **HRMS** (ESI) [M + Na]$^+$ m/z: calculated for $C_{34}H_{36}Cl_2N_2O_6Na$ 661.1843; found: 661.1823.

(3*S*,4*S*)-3-(2-(1-(4-chlorobenzoyl)-5-methoxy-2-methyl-1H-indol-3-yl)ethyl)-1-phenyl-4-((3a*R*,5*R*,5a*S*,8a*S*,8b*R*)-2,2,7,7-tetramethyltetrahydro-3a*H*-bis([1,3]dioxolo)[4,5-*b*:4',5'-*d*]pyran-5-yl)azetidin-2-one

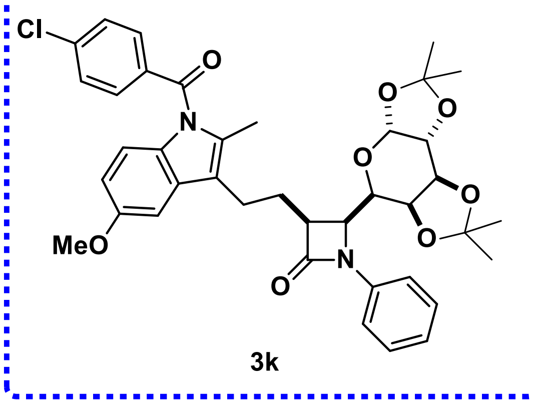

3k

The product **3k** was obtained as a yellowish foam in 46% yield (64.5 mg) and *d.r.* = 5:1. The reaction was not completed even after seven hours of reaction and several attempts of purification was performed to isolate the product but unsuccessful in all cases. Inseparable mixture of alkylated product (32%) and **2e** (14%). The crude material was purified by flash column chromatography (gradient – EtOAc in Hexanes 5 – 10 %). **^{1}H NMR (400 MHz, CDCl$_3$)** δ 7.58 (d, *J* = 7.4 Hz, 1H), 7.51 (d, *J* = 7.5 Hz, 2H), 7.39 (d, *J* = 7.5 Hz, 2H), 7.23 (t, *J* = 7.8 Hz, 2H), 7.04 (d, *J* = 11.0 Hz, 2H), 6.80 (d, *J* = 9.0 Hz, 1H), 6.57 (d, *J* = 9.0 Hz, 1H), 5.46 (d, *J* = 5.1 Hz, 1H), 4.39 (t, *J* = 6.7 Hz, 2H), 4.18 – 4.11 (m, 2H), 3.93 (d, *J* = 7.7 Hz, 1H), 3.83 (d, *J* = 9.3 Hz, 1H), 3.78 (s, 3H), 3.37 – 3.29 (m, 1H), 3.07 – 2.97 (m, 1H), 2.87 (dt, *J* = 14.5, 7.4 Hz, 1H), 2.37 (dt, *J* = 13.8, 6.8 Hz, 1H), 2.31 (s, 3H), 2.03 (dt, *J* = 15.6, 6.9 Hz, 1H), 1.44 (s, 3H), 1.31 (s, 3H), 1.20 (s, 6H) ppm. **^{13}C NMR (101 MHz, CDCl$_3$)** δ 169.7, 168.4, 156.0, 139.0, 137.5, 134.7, 134.4, 131.6, 131.2, 131.0, 129.2, 129.1, 128.8, 124.6, 119.2, 119.1, 118.2, 115.0, 111.5, 109.1, 108.5, 101.6, 96.3, 70.9, 70.8, 70.6, 67.6, 59.3, 55.8, 53.4, 51.3, 26.0, 25.2, 24.8, 23.7, 21.9, 13.5 ppm. **HRMS** (ESI) [M + Na]$^+$ m/z: calculated for $C_{39}H_{41}ClN_2O_8Na$ 723.2444; found: 723.2426.

***tert*-butyl (2-methyl-1-((3*S*,4*S*)-2-oxo-1-phenyl-4-((3a*R*,5*R*,5a*S*,8a*S*,8b*R*)-2,2,7,7-tetramethyltetrahydro-3aH-bis([1,3]dioxolo)[4,5-*b*:4',5'-*d*]pyran-5-yl)azetidin-3-yl)propan-2-yl)carbamate**

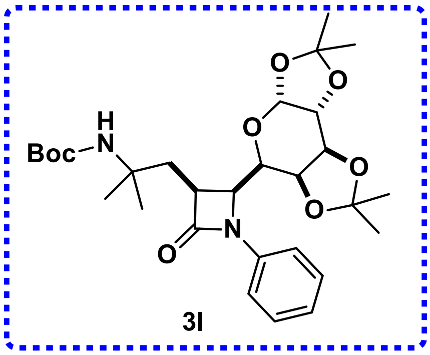

3l

The product **3l** was obtained as a viscous colorless oil in 37% yield (40.4 mg) and *d.r.* > 20:1. The crude material was purified by flash column chromatography (gradient – EtOAc in Hexanes 10 – 20 %). **^{1}H NMR (400 MHz, CDCl$_3$)** δ 7.59 (d, *J* = 8.1 Hz, 2H), 7.29 (t, *J* = 7.8 Hz, 2H), 7.09 (t, *J* = 7.4 Hz, 1H), 5.52 (d, *J* = 5.0 Hz, 1H), 5.04 (t, *J* = 8.7 Hz, 1H), 4.53 (dd, *J* = 9.3, 5.8 Hz, 1H), 4.45 (d, *J* = 8.1 Hz, 1H), 4.22 (dd, *J* = 5.0, 1.3 Hz, 1H), 3.96 (d, *J* = 7.7 Hz, 1H), 3.79 (d, *J* = 9.4 Hz, 1H), 3.64 (t, *J* = 9.9 Hz, 1H), 3.49 (dd, *J* = 11.7, 5.7 Hz, 1H), 1.96 (s, 1H), 1.53 (s, 3H), 1.47 (s, 3H), 1.42 (s, 9H), 1.29 (s, 3H), 1.26 (s, 3H), 0.91 (d, *J* = 6.7 Hz, 3H), 0.87 (d, *J* = 6.8 Hz, 3H) ppm. **^{13}C NMR (101 MHz, CDCl$_3$)** δ 169.9, 156.6, 137.4, 128.7, 124.7, 119.2, 109.1, 108.4, 96.3, 70.8, 70.6, 70.5, 67.9, 54.9, 53.4, 50.2, 31.9, 28.5, 28.3, 26.1, 26.0, 24.8, 23.6, 18.9, 17.1 ppm. **HRMS** (ESI) [M + Na]$^+$ m/z: calculated for $C_{29}H_{42}N_2O_8Na$ 569.2833; found: 569.2864.

tert-butyl 2-(((3*S*,4*S*)-2-oxo-1-phenyl-4-((3a*R*,5*R*,5a*S*,8a*S*,8b*R*)-2,2,7,7-tetramethyltetrahydro-3a*H*-bis([1,3]dioxolo)[4,5-*b*:4',5'-*d*]pyran-5-yl)azetidin-3-yl)methyl)piperidine-1-carboxylate

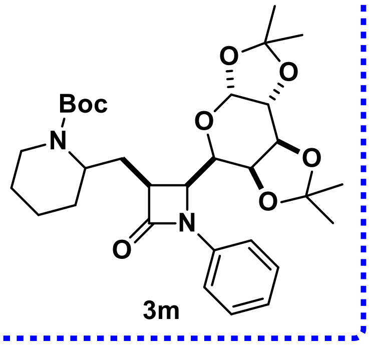

3m

The product **3m** was obtained as a viscous colorless oil in 39% yield (44.6 mg) and *d.r* = 20:1 (C3 *β*-lactam) and 1.1:1 (*α*-*N*-Boc). The crude material was purified by flash column chromatography (gradient – EtOAc in Hexanes 10 – 20 %). **^{1}H NMR (400 MHz, CDCl$_3$ diastereoisomeric mixture)** δ 7.60 (dt, *J* = 8.5, 1.4 Hz, 2H), 7.30 (t, *J* = 7.8 Hz, 2H), 7.09 (td, *J* = 7.6, 1.5 Hz, 1H), 5.54 (d, *J* = 5.0 Hz, 1H), 5.52 (d, *J* = 5.0 Hz, 1H), 4.66 – 4.56 (m, 1H), 4.47 (tdd, *J* = 16.6, 9.5, 5.7 Hz, 2H), 4.22 (dd, *J* = 5.0, 1.9 Hz, 1H), 4.14 – 3.96 (m, 2H), 3.83 (ddd, *J* = 9.3, 7.6, 1.4 Hz, 1H), 3.41 – 3.31 (m, 1H), 2.93 – 2.75 (m, 1H), 2.20 (ddd, *J* = 15.1, 10.4, 4.9 Hz, 1H), 1.72 – 1.53 (m, 7H), 1.52 – 1.38 (m, 16H), 1.37 – 1.27 (m, 6H) ppm. **^{13}C NMR (101 MHz, CDCl$_3$ diastereoisomeric mixture)** δ 169.5, 155.5, 137.7, 137.5, 128.7, 124.5, 124.4, 119.2, 118.9, 109.1, 109.0, 108.4, 96.3, 96.2, 70.9, 70.8, 70.6, 68.0, 67.7, 53.6, 52.8, 49.5, 49.1, 28.7, 28.5, 26.1, 26.0, 26.0, 25.9, 24.8, 23.6, 23.6, 19.0 ppm. **HRMS** (ESI) [M + Na]$^+$ m/z: calculated for $C_{31}H_{44}N_2O_8Na$ 595.2990; found: 595.2993.

(3*S*,4*S*)-1-phenyl-4-((3a*R*,5*R*,5a*S*,8a*S*,8b*R*)-2,2,7,7-tetramethyltetrahydro-5*H*-bis([1,3]dioxolo)[4,5-*b*:4',5'-*d*]pyran-5-yl)-3-(((3a*R*,5*R*,5a*S*,8a*S*,8b*R*)-2,2,7,7-tetramethyltetrahydro-5*H*-bis([1,3]dioxolo)[4,5-*b*:4',5'-*d*]pyran-5-yl)methyl)azetidin-2-one

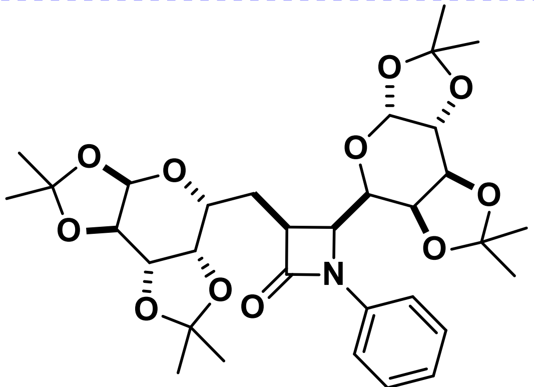
3n

The product **3n** was obtained as a white foam in 47% yield (58.0 mg) and *d.r.* > 20:1. The crude material was purified by flash column chromatography (gradient – EtOAc in Hexanes 5 – 15 %). **^{1}H NMR (400 MHz, CDCl$_3$)** δ 7.61 – 7.56 (m, 2H), 7.31 – 7.26 (m, 2H), 7.08 (t, *J* = 7.4 Hz, 1H), 5.52 (d, *J* = 5.0 Hz, 1H), 5.29 (d, *J* = 2.5 Hz, 1H), 4.52 (d, *J* = 5.5 Hz, 1H), 4.49 – 4.43 (m, 2H), 4.26 (dd, *J* = 9.6, 5.5 Hz, 1H), 4.22 – 4.17 (m, 2H), 3.99 (dd, *J* = 8.1, 1.1 Hz, 1H), 3.92 – 3.83 (m, 2H), 3.63 – 3.54 (m, 1H), 2.49 (ddd, *J* = 14.4, 6.4, 5.0 Hz, 1H), 2.18 – 2.06 (m, 1H), 1.51 (s, 3H), 1.49 (s, 3H), 1.48 (s, 3H), 1.47 (s, 3H), 1.36 (s, 3H), 1.35 (s, 3H), 1.28 (s, 3H), 1.26 (s, 3H) ppm. **^{13}C NMR (101 MHz, CDCl$_3$)** δ 169.1, 137.5, 128.7, 124.4, 119.1, 110.6, 109.0, 108.8, 108.5, 96.9, 96.2, 76.1, 74.6, 74.1, 70.8, 70.7, 70.6, 70.2, 67.5, 52.9, 47.6, 28.1, 28.0, 27.6, 26.14, 26.11, 26.0, 25.9, 24.8, 23.7 ppm. **HRMS** (ESI) $[M + Na]^+$ m/z: calculated for $C_{32}H_{43}NO_{11}Na$ 640.2728; found: 640.2712.

3*S*,4*S*)-4-((*S*)-2,2-dimethyl-1,3-dioxolan-4-yl)-1-phenyl-3-((1-(phenylsulfonyl)piperidin-4-yl)methyl)azetidin-2-one

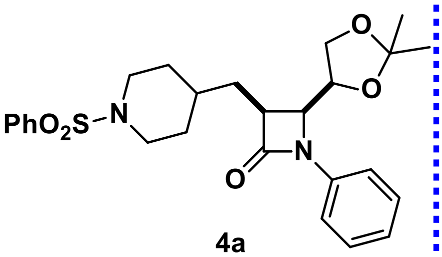
4a

The product **4a** was obtained as a white solid in 67% yield (64.9 mg) and *d.r.* > 20:1. The crude material was purified by flash column chromatography (gradient – EtOAc in Hexanes 20 – 30 %). **^{1}H NMR (400 MHz, CDCl$_3$)** δ 7.69 (d, *J* = 7.7 Hz, 2H), 7.57 – 7.51 (m, 1H), 7.47 (t, *J* = 7.2 Hz, 2H), 7.34 (d, *J* = 7.9 Hz, 2H), 7.22 (dd, *J* = 15.2, 7.7 Hz, 2H), 7.03 (t, *J* = 7.3 Hz, 1H), 4.32 (t, *J* = 5.3 Hz, 1H), 4.16 (dd, *J* = 12.0, 6.0 Hz, 1H), 3.83 (t, *J* = 7.0 Hz, 1H), 3.78 – 3.68 (m, 2H), 3.39 (dt, *J* = 9.9, 5.1 Hz, 1H), 2.21 (t, *J* = 11.5 Hz, 2H), 1.80 (dd, *J* = 21.2, 13.5 Hz, 2H), 1.63 – 1.45 (m, 4H), 1.39 – 1.18 (m, 8H)

ppm. **^{13}C NMR (101 MHz, CDCl$_3$)** δ 167.9, 137.3, 136.1, 132.8, 129.1, 129.0, 127.7, 124.8, 118.7, 109.0, 75.4, 66.5, 55.6, 48.8, 46.48, 46.43, 33.6, 32.3, 31.8, 31.0, 26.3, 25.1 ppm. **HRMS** (ESI) $[M + H]^+$ m/z: calculated for $C_{26}H_{32}N_2O_5SH$ 485.2110; found: 485.2103.

(3*S*,4*S*)-4-((3a*S*,4*R*,6*S*,6a*S*)-6-methoxy-2,2-dimethyltetrahydrofuro[3,4-*d*][1,3]dioxol-4-yl)-1-phenyl-3-((1-(phenylsulfonyl)piperidin-4-yl)methyl)azetidin-2-one

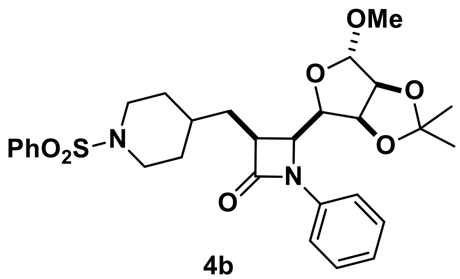

4b

The product **4b** was obtained as a white foam in 50% yield (55.6 mg) and *d.r.* > 20:1. The crude material was purified by flash column chromatography (isocratic – EtOAc in Hexanes 20 %). **^{1}H NMR (400 MHz, CDCl$_3$)** δ 7.75 (dd, *J* = 8.4, 1.4 Hz, 2H), 7.60 – 7.51 (m, 5H), 7.32 – 7.25 (m, 2H), 7.09 (ddd, *J* = 7.5, 4.2, 1.2 Hz, 1H), 4.82 (s, 1H), 4.59 (dd, *J* = 9.4, 5.7 Hz, 1H), 4.47 (d, *J* = 5.6 Hz, 1H), 4.42 (dd, *J* = 5.7, 2.7 Hz, 1H), 3.95 (dd, *J* = 9.4, 2.7 Hz, 1H), 3.85 – 3.74 (m, 2H), 3.53 (dt, *J* = 11.3, 5.7 Hz, 1H), 3.29 (s, 3H), 2.30 (td, *J* = 11.9, 2.6 Hz, 2H), 1.99 – 1.91 (m, 1H), 1.89 – 1.66 (m, 5H), 1.51 (s, 3H), 1.39 – 1.27 (m, 5H) ppm. **^{13}C NMR (101 MHz, CDCl$_3$)** δ 168.2, 137.1, 136.1, 132.8, 129.0, 128.8, 127.7, 124.6, 119.1, 112.6, 107.8, 85.3, 80.1, 78.8, 55.3, 52.3, 49.3, 46.4, 46.4, 33.0, 32.6, 32.0, 30.8, 26.3, 24.9 ppm. **HRMS** (ESI) $[M + H]^+$ m/z: calculated for $C_{29}H_{36}N_2O_7SH$ 557.2316; found: 557.2313.

(3*S*,4*S*)-4-((3a*S*,4*R*,6*S*,6a*S*)-6-(benzyloxy)-2,2-dimethyltetrahydrofuro[3,4-*d*][1,3]dioxol-4-yl)-1-phenyl-3-((1-(phenylsulfonyl)piperidin-4-yl)methyl)azetidin-2-one

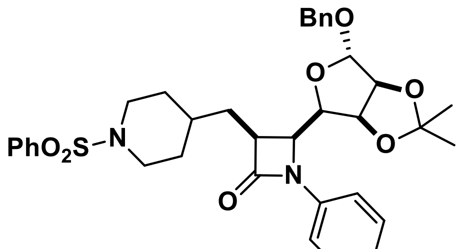

4c

The product **4c** was obtained as a white foam in 65% yield (201.4 mg; 0.49 mmol scale) and *d.r.* > 20:1. The crude material was purified by flash column chromatography (gradient – DCM in Hexanes 75 – 100 %; EtOAc in DCM 5%). **^{1}H NMR (400 MHz, CDCl$_3$)** δ 7.75 (d, *J* = 7.6 Hz, 2H), 7.59 (t, *J* = 7.4 Hz, 1H), 7.52 (t, *J* = 7.6 Hz, 2H), 7.48 (d, *J* = 8.5 Hz, 2H), 7.40 (d, *J* = 8.5 Hz, 2H), 7.34 (t, *J* = 7.2 Hz, 2H), 7.30 (t, *J* = 7.2 Hz, 1H), 7.25 (t, *J* = 6.5 Hz, 3H), 5.03 (s, 1H), 4.64 – 4.55 (m, 3H), 4.45 (dd, *J* = 10.0, 5.5 Hz, 2H), 4.05 (dd, *J* = 9.2,

2.8 Hz, 1H), 3.80 (dt, J = 15.9, 7.9 Hz, 2H), 3.61 – 3.54 (m, 1H), 2.33 – 2.24 (m, 2H), 1.99 – 1.66 (m, 6H), 1.50 (s, 3H), 1.39 – 1.26 (m, 5H) ppm. **^{13}C NMR (101 MHz, $CDCl_3$)** δ 168.1, 136.7, 136.1, 136.0, 132.8, 131.8, 129.0, 128.7, 128.2, 127.7, 127.7, 120.6, 117.4, 112.8, 105.6, 85.4, 80.0, 78.8, 69.5, 52.5, 49.5, 46.4, 46.4, 33.0, 32.6, 32.1, 30.7, 26.2, 24.9 ppm. **HRMS** (ESI) $[M + Na]^+$ m/z: calculated for $C_{35}H_{40}N_2O_7SNa$ 655.2448; found: 655.2440.

(3*S*,4*S*)-4-((3a*R*,5*R*,6*S*,6a*R*)-6-methoxy-2,2-dimethyltetrahydrofuro[2,3-*d*][1,3]dioxol-5-yl)-1-phenyl-3-((1-(phenylsulfonyl)piperidin-4-yl)methyl)azetidin-2-one

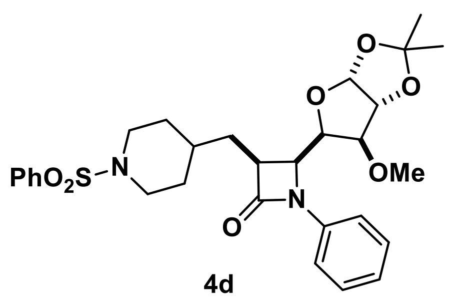

4d

The product **4d** was obtained as a white foam in 45 % yield (50.1 mg) and *d.r.* = 19:1. The crude material was purified by flash column chromatography. **^{1}H NMR (400 MHz, $CDCl_3$)** δ 7.76 – 7.72 (m, 2H), 7.61 – 7.54 (m, 1H), 7.54 – 7.48 (m, 2H), 7.36 – 7.27 (m, 4H), 7.13 – 7.08 (m, 1H), 5.79 (d, J = 3.7 Hz, 1H), 4.57 (d, J = 3.8 Hz, 1H), 4.48 (dd, J = 9.5, 5.6 Hz, 1H), 4.05 (dd, J = 9.5, 2.7 Hz, 1H), 3.80 – 3.73 (m, 2H), 3.48 (dt, J = 8.8, 6.8 Hz, 1H), 3.36 (d, J = 2.7 Hz, 1H), 3.29 (s, 3H), 2.25 (tdd, J = 11.7, 4.1, 2.4 Hz, 2H), 1.88 – 1.59 (m, 5H), 1.41 (s, 3H), 1.35 – 1.26 (m, 5H) ppm. **^{13}C NMR (101 MHz, $CDCl_3$)** δ 168.5, 136.6, 136.0, 134.3, 132.7, 132.7, 129.0, 129.0, 127.7, 124.9, 123.5, 119.3, 119.3, 112.0, 104.2, 83.2, 80.1, 79.0, 56.6, 51.6, 49.7, 46.5, 46.4, 33.3, 31.8, 31.1, 30.7, 26.8, 26.2 ppm. **HRMS** (ESI) $[M + Na]^+$ m/z: calculated for $C_{29}H_{36}N_2O_7SNa$ 579.2135; found: 579.2131.

(3*S*,4*S*)-1-(*tert*-butyl)-3-((1-(phenylsulfonyl)piperidin-4-yl)methyl)-4-((3a*R*,5*R*,5a*S*,8a*S*,8b*R*)-2,2,7,7-tetramethyltetrahydro-3a*H*-bis([1,3]dioxolo)[4,5-*b*:4',5'-*d*]pyran-5-yl)azetidin-2-one

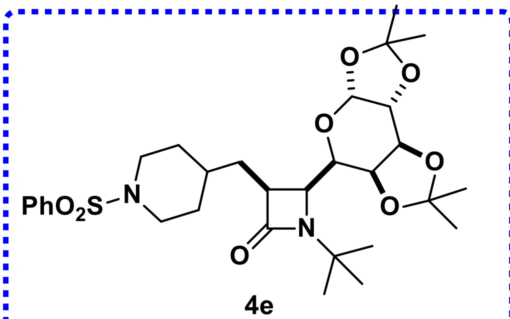

4e

The product **4e** was obtained as a white foam in 50% yield (59.2 mg) and *d.r.* > 20:1. The crude material was purified by flash column chromatography (gradient – EtOAc in Hexanes 20 – 50 %). R_f = 0.35 (50% EtOAc in Hexanes). **^{1}H NMR (400 MHz, $CDCl_3$)** δ 7.72 (d, J = 7.2 Hz, 2H), 7.59 – 7.47 (m,

3H), 5.51 (d, J = 5.3 Hz, 1H), 4.58 (dd, J = 8.1, 2.0 Hz, 1H), 4.29 (d, J = 8.1 Hz, 1H), 4.25 (dd, J = 5.2, 2.0 Hz, 1H), 3.88 (d, J = 3.6 Hz, 2H), 3.78 – 3.70 (m, 2H), 3.16 (dt, J = 10.4, 4.8 Hz, 1H), 2.26 – 2.18 (m, 2H), 1.89 (d, J = 13.4 Hz, 1H), 1.76 (ddd, J = 28.5, 10.9, 6.5 Hz, 4H), 1.45 (d, J = 12.4 Hz, 6H), 1.35 – 1.26 (m, 17H) ppm. **^{13}C NMR (101 MHz, $CDCl_3$)** δ 172.2, 136.1, 132.7, 129.04, 128.99, 127.8, 109.1, 108.2, 96.4, 71.4, 71.1, 70.3, 67.6, 54.7, 54.5, 49.0, 46.7, 46.6, 33.5, 32.6, 32.4, 30.6, 28.74, 28.65, 26.0, 25.9, 24.7, 23.6 ppm. **HRMS** (ESI) $[M + Na]^+$ m/z: calculated for $C_{30}H_{44}N_2O_8SNa$ 615.2711; found: 615.2697.

2.9 Synthetic application

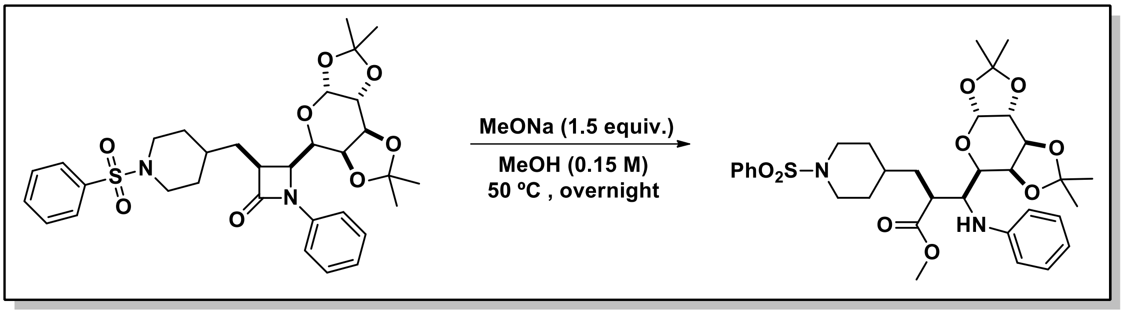

Scheme S16: Cleavage of the β-lactam core to obtain *β*-glicoamino ester

Cleavage at position N1-C2[37]: To a solution of **3e** (0.93 mmol, 1 equiv.; 570 mg) in MeOH (0.12 M, 8 mL), MeONa (1.4 mmol, 1.5 equiv.; 75 mg) was added. This reaction solution remained under vigorous stirring overnight at 50 ºC in an oil bath. At the end, H_2O (10 mL) was added, which caused partial precipitation of the product as a white solid (205 mg, 33 %), which was recovered by filtration. The reaction solution was extracted with AcOEt (3 x 30 mL) and the combined organic phases were washed with brine (1 x 30 mL), dried over anhydrous Na_2SO_4, filtered and the solvent evaporated under reduced pressure, affording another portion of the *β*-glycoamino ester as a white solid (200 mg, 33%). Final yield 66% and *d.r.* = 20:1.

2.9.1 Spectroscopic data of the compound

(2*S*,3*S*)-methyl 3-(phenylamino)-2-((1-(phenylsulfonyl)piperidin-4-yl)methyl)-3-((3a*R*,5*S*,5a*S*,8a*S*,8b*R*)-2,2,7,7-tetramethyltetrahydro-3aH-bis([1,3]dioxolo)[4,5-*b*:4',5'-*d*]pyran-5-yl)propanoate

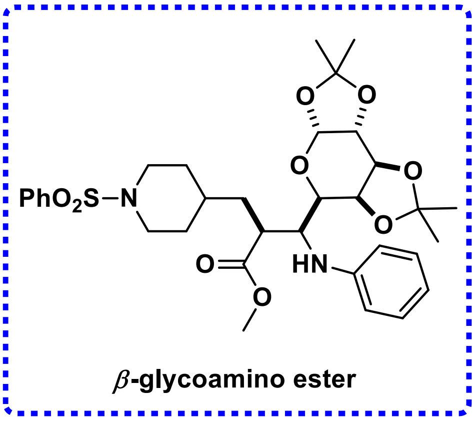

***β*-glycoamino ester** was obtained as a white solid by aqueous precipitation in 66 % yield (395.4 mg) and *d.r.* > 20:1. **^{1}H NMR (400 MHz, CDCl$_3$)** δ 7.76 – 7.72 (m, 2H), 7.60 – 7.55 (m, 1H), 7.2 (t, J = 7.2 Hz, 2H), 7.10 (t, J = 7.7 Hz, 2H), 6.69 – 6.63 (m, 3H), 5.49 (d, J = 5.0 Hz, 1H), 4.55 (dd, J = 8.0, 2.2 Hz, 1H), 4.37 – 4.34 (m, 1H), 4.33 – 4.26 (m, 2H), 3.78 (t, J = 10.8 Hz, 2H), 3.57 (d, J = 9.5 Hz, 1H), 3.30 (s, 3H), 2.90 (dt, J = 11.9, 3.3 Hz, 1H), 2.16 (ddt, J = 21.0, 11.9, 5.9 Hz, 2H), 1.79 – 1.53 (m, 6H), 1.50 (d, J = 4.0 Hz, 6H), 1.40 (ddd, J = 16.7, 8.1, 3.3 Hz, 2H), 1.33 (s, 3H), 1.28 (s, 3H). **^{13}C NMR (101 MHz, CDCl$_3$)** δ 174.7, 147.7, 136.2, 132.7, 129.1, 129.15, 127.8, 118.1, 114.3, 109.2, 108.5, 96.7, 71.0, 70.9, 70.7, 67.7, 55.0, 51.6, 46.6, 43.2, 33.6, 32.7, 30.8, 30.5, 26.2, 24.9, 24.4. **HRMS** (ESI) [M + H]$^+$ m/z: calculated for $C_{33}H_{44}N_2O_9SH$ 645.2840; found: 645.2831.

3. Mechanistic studies

3.1 UV/Vis absorption spectroscopy

Experimental details: UV/Vis absorption spectra were measured with a 1.0 cm quartz cell using a Shimadzu UV/Vis spectrophotometer, model 1800 at 20 ºC operating with a resolution of up to 0.1 nm in a spectral window of 190-1100 nm. Absorption spectra of individual reaction components **1e**, **2e** and **HE** and mixtures were recorded in DMSO solution (0.1 M).

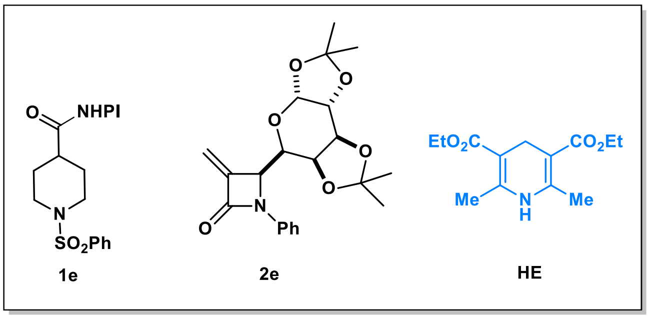

Scheme S17: Redox-active ester, lactam and HE used in the UV/Vis experiment

Considerations: The lack of reactivity in the absence of Hantzsch ester (**HE**) is a good indicative of the formation of the EDA complex. Additionally, the formation of EDA complexes between *N*-(acyloxy)phthalimides and **HE** suggested in this work is well supported by

literature. However, in order to verify the formation and the possibility of a ternary EDA-complex, we performed an UV/Vis experiment and NMR titration.

Visual evidence: It is possible to observe that the compound 3-exomethylene *β*-lactam (**2e**) absorbs around 480 nm. In this way, it is expected that its mixture with the other components of the reaction, that exhibit a yellow color, will absorb in regions of lower energy. Thus, no significant changes in UV/Vis absorptions were observed for the solutions **1e**, **2e**, **1e**+**2e** or **2e**+**HE** and the absorbance of **2e** in the presence of **HE** is reduced in 480 nm. However, the intensification of the color is observed in the solution containing **1e**+**HE**. It can be attributed to the formation of an EDA complex between the reactants.

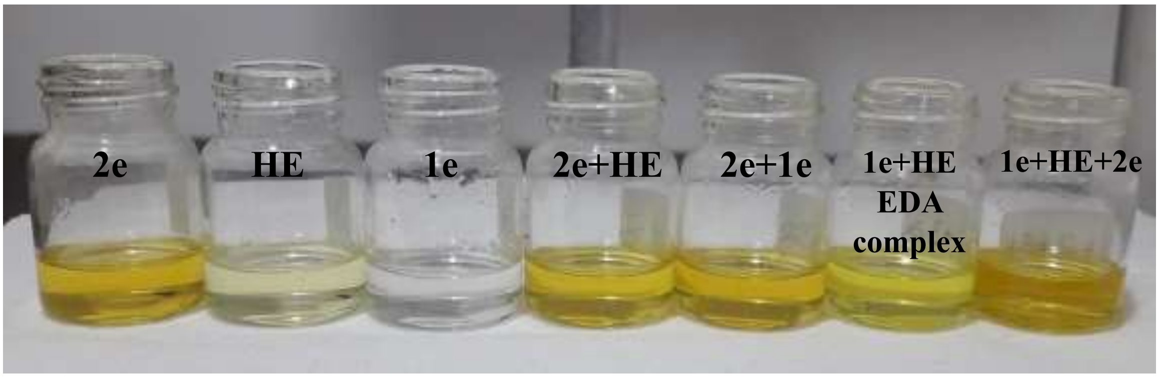

Figure S2: Visual evidence of the formations of EDA complex between **HE** and **1e**

Spectroscopic evidence: The charge-transfer (CT) band was observed in visible-light region with **1e** + **HE**, where a clear bathochromic displacement could be observed, which is an indicative of an electron donor-acceptor complex. The maximum absorptions observed between the mixtures combinations of the reaction components were around 435 - 438 nm.

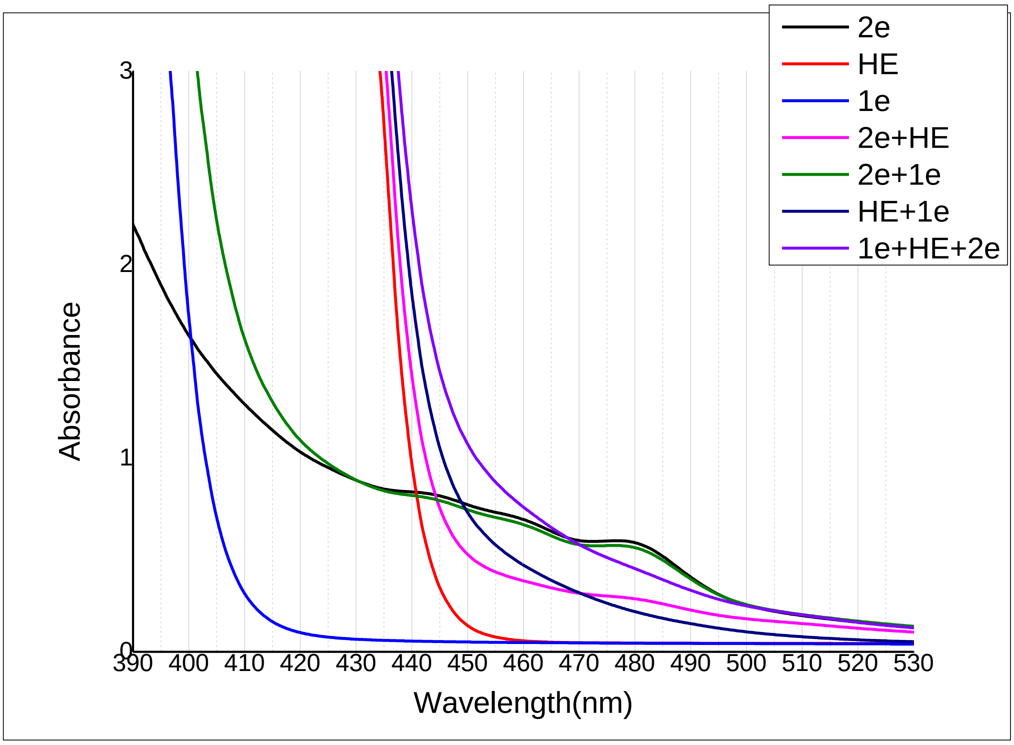

Figura S3: UV-Vis spectra of the formation of EDA complex between **HE** and **1e**

3.2 NMR titration

^{1}H NMR experiments were performed by the preparation of DMSO-d^{6} solutions containing **HE** and **1e** in three different ratios, keeping constant the amount of **HE** (0.05 mol/L) and increasing the amount of **1e**. The figure below shows the expansion of the NMR spectras collected on which the **HE** methylenic protons shift were monitored. From this set of experiments, it is possible to observe the change in the chemical shifts of the monitored hydrogens with the addition of increasing amounts of **1e** to downfield. In presence of **2b**, the shift is displaced to upfield, ruling out the possibility of a ternary EDA-complex system. So, 3-exomethylene *β*-lactam seems to disrupts the EDA complexation between HE and phthalimide.

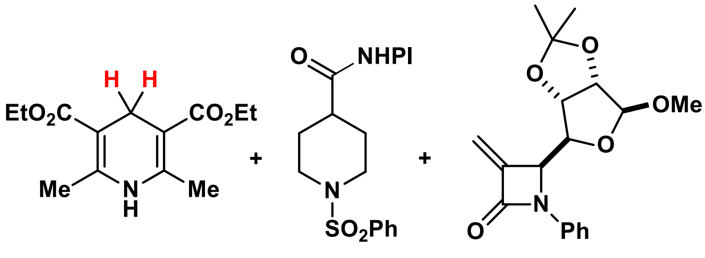

HE **1e** **2b**

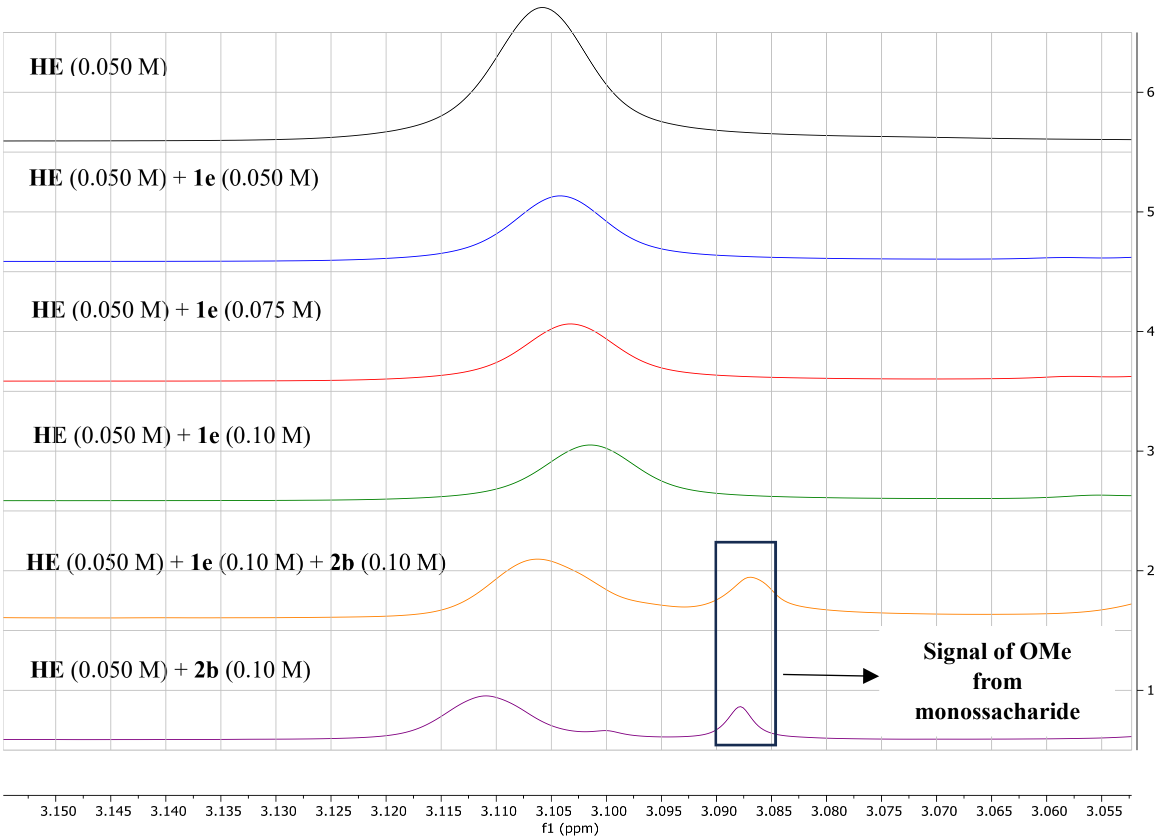

Figura S4: NMR titration for monitoring **HE** methylenic protons

3.3 Radical trapping experiment

Reaction condition as described in the general procedure 2.8 with the addition of the radical scavenger TEMPO (0.4 mmol, 2 equiv.). The reaction solution was not treated as described in the general procedure and the crude reaction solution was injected in the HRMS.

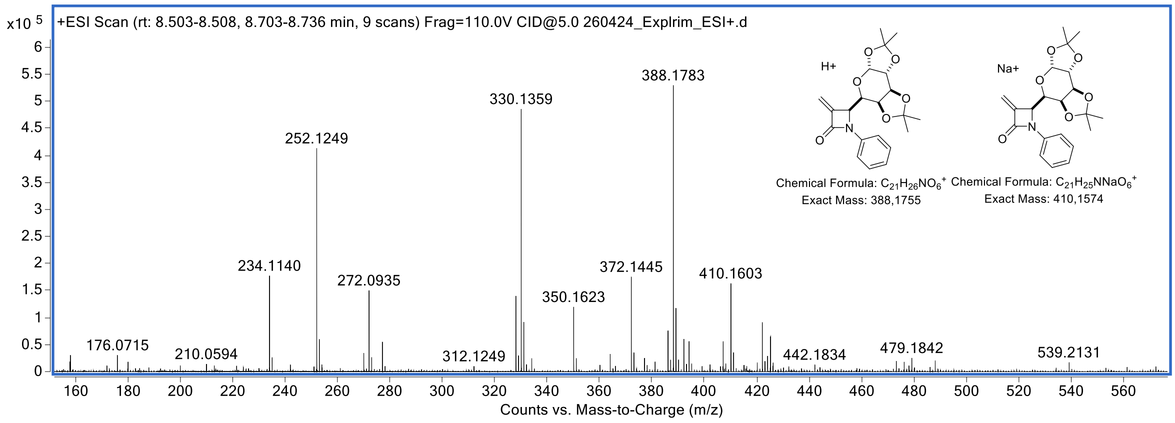

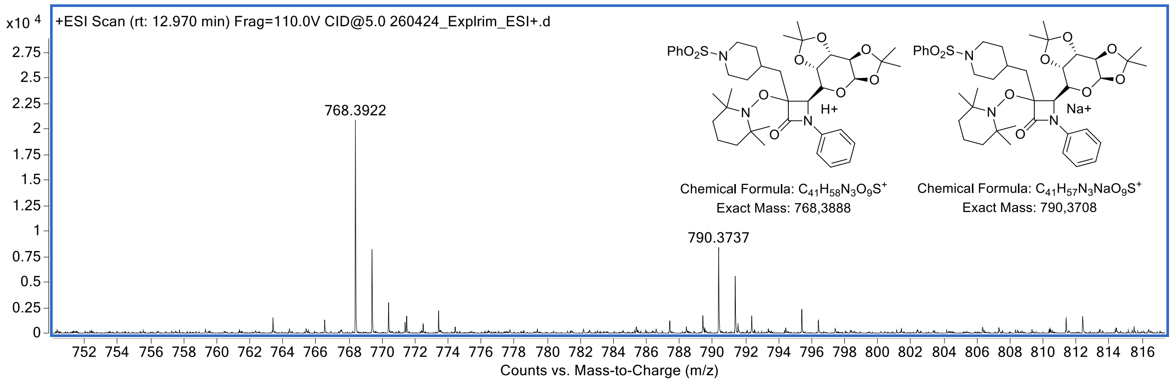

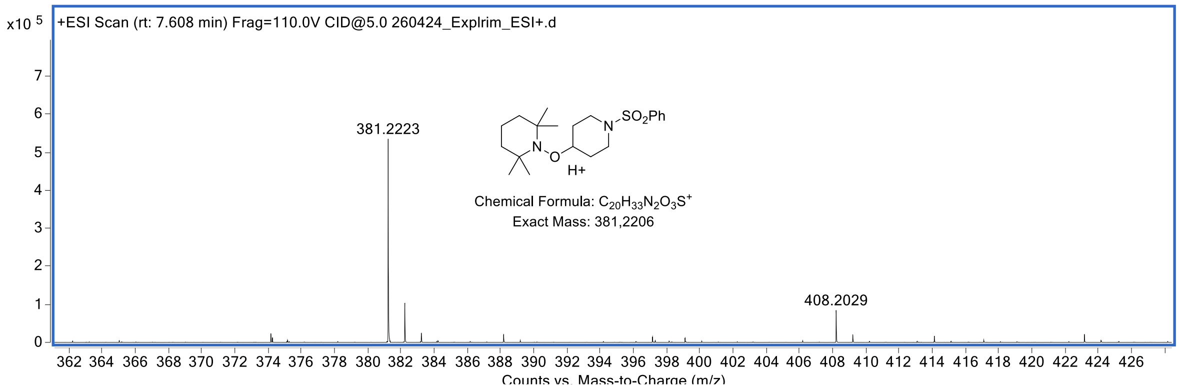

Figure S5: TEMPO experiment: substrate not completed consumed and products of radical-radical coupling between intermediates and TEMPO captured

4. Scale-up of the photoinduced EDA reaction

A

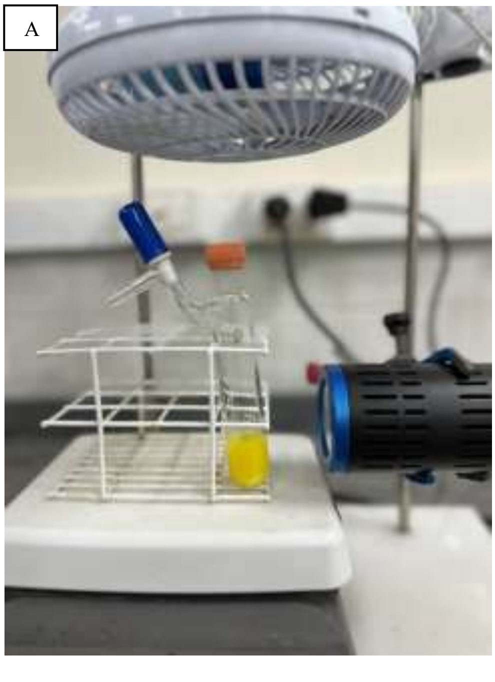

B

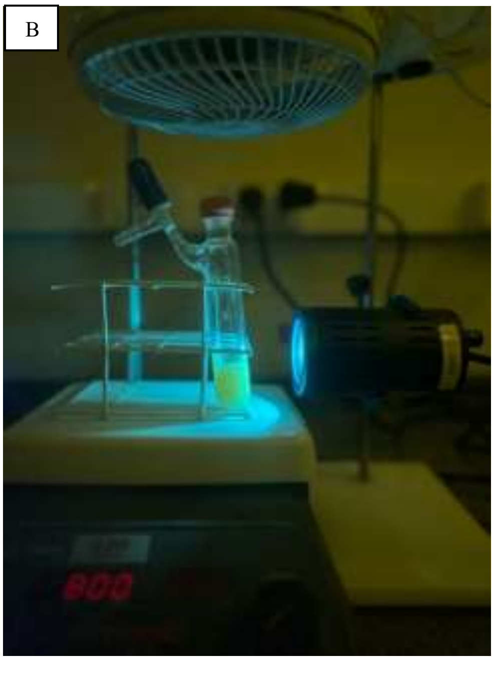

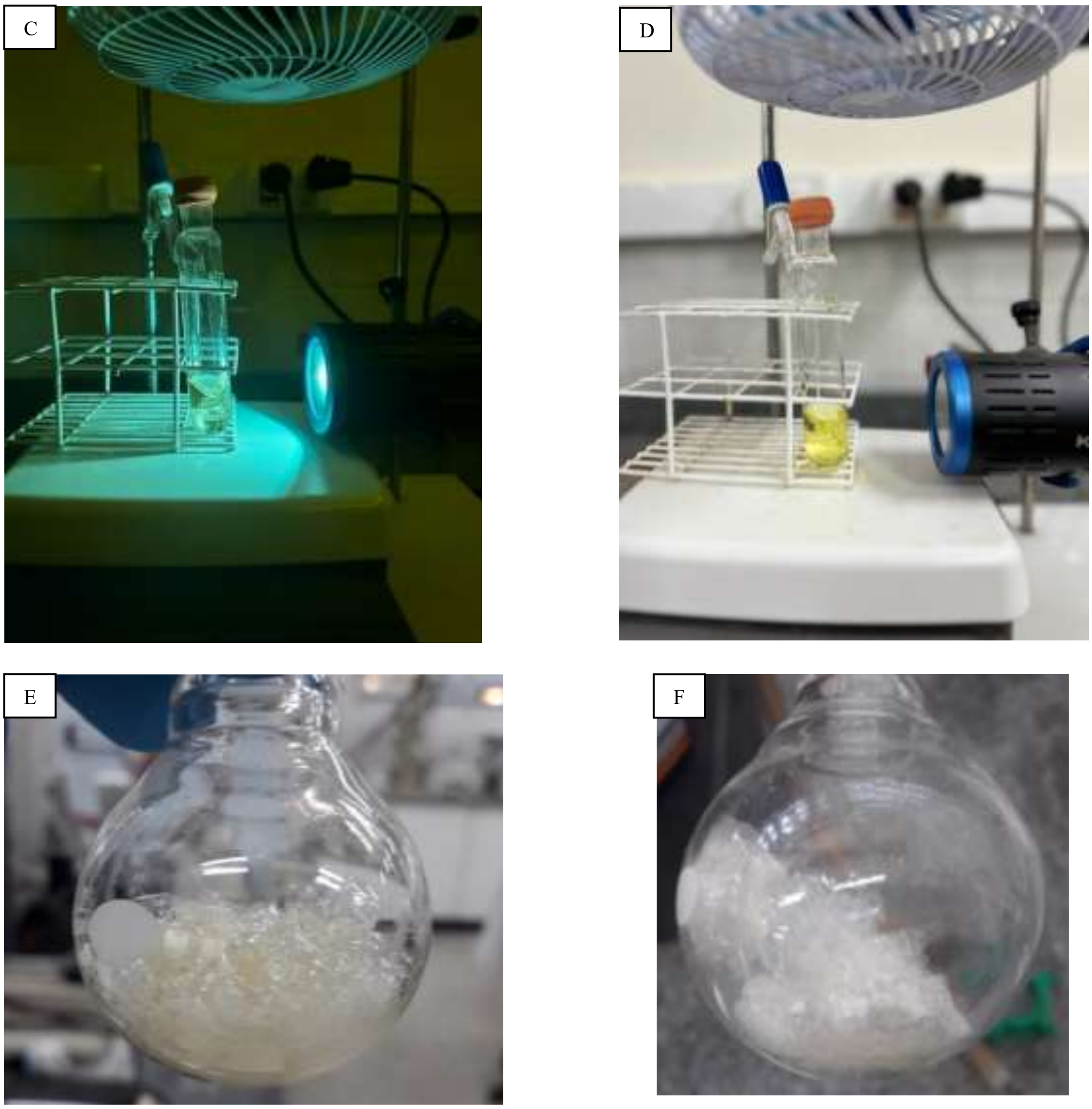

Figure S6: A) Heterogeneous reaction mixture before turning on the LED lamp. B) Reaction mixture when LED lamp is turned on. C) Homogeneous reaction mixture after 20 minutes of LED lamp was turn on D) Homogeneous reaction mixture with the LED lamp off at the end of the reaction (2h). E) Isolated product **3f** (1.15 mmol scale). F) Isolated product **4c** (0.49 mmol scale)

5. X-ray analysis parameters

General procedure for the single-crystal growth: In a 15 ml vial, 35 mg of compound **4a** was solubilized in 6 ml of DCM:MeOH (5:1) as solvent system. The vial was sealed with a stopper, and several holes were made in it using a needle. The sample was placed on a vibration-free

surface and left for 8 days at room temperature to allow the solvent evaporation and crystal growth.

Description of the instrumentation used: The crystallographic data for **4a** (Table 1) was collected on a diffractometer, Bruker D8 VENTURE model with Cu- Kα radiation (1,54178 Å) at 296 K. The D8 VENTURE incorporates a two-dimensional CMOS Photon III-C14 next-generation detector. All diffraction patterns were integrated with the APEX4 software and multi-scan absorption correction with SADABS.[38] The data set was refined with ShelXL[39] by least squares using the OLEX2 1.5 version.[40] The structures were solved with the ShelXT program[41] using the Intrinsic Phasing method. All the atoms were refined with anisotropic parameters. The hydrogen atoms were calculated and refined from isotropic thermal parameters with riding coordinates. For the **4a**, the displacement parameters of the atoms C35-C36-O8-C37-O7 were treated using RIGU with sigma for 1-2 distances of 0.004 and sigma for 1-3 distances of 0.004. The absolute configuration of **4a** was established by anomalous-dispersion effects in diffraction measurements on the crystal, with a Friedel pairs fraction of 0.984 (full). Finally the twinned data refinement show a scales of 0.937(13) for the (S,S,S) component.

Table 1. Crystal data, data collection, and structure refinement for **4a** compound.

Compound	**4a**
Empirical formula	$C_{26}H_{32}N_2O_5S$
Formula weight (mol/g)	483.62
Temperature (K)	296
Crystal system	Orthorhombic
Space group	$P2_12_12_1$
a (Å)	6.049(1)
b (Å)	24.322(4)
c (Å)	33.702(6)
Volume (Å^3)	4958(14)
Z	4
r calcg (cm^3)	1.298
m (mm^{-1})	1.483
F(000)	2064
Crystal size (mm^3)	0.68 × 0.12 × 0.12
Radiation	Cu Ka (l= 1.54178)
2q range for data collection (°)	7.4 to 68.3
Index ranges	$-7 \leq h \leq 6$ / $-29 \leq k \leq 29$ / $-40 \leq l \leq 40$
Reflections collected	144932
Independent reflections	8952 [R_{int} = 0.086]
Data/restraints/parameters	8821/618
Goodness-of-fit on F^2	1.036
Final R indexes [all data]	R^1 = 0.0320 / wR^2 = 0.0862
Largest diff. peak/hole / e Å^{-3}	0.35/-0.27

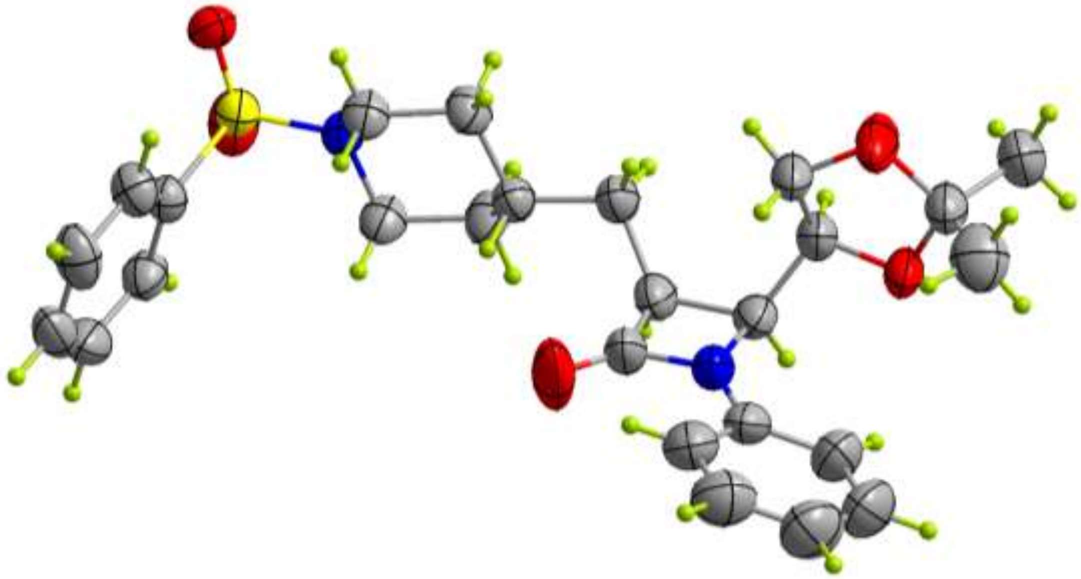

Figure S7: View of the molecular structure of **4a** (one selected molecule of the asymmetric unit). Atomic displacement ellipsoids are drawn at the 50% probability level for all non-hydrogen atoms.

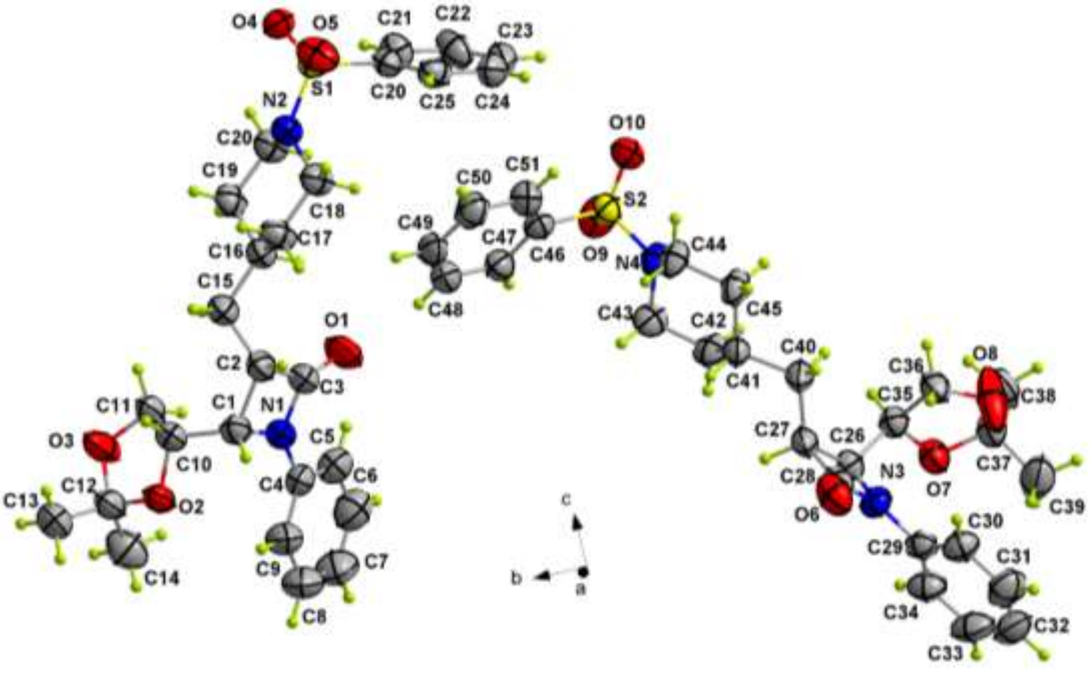

Figure S8: View of the asymmetric unit of **4a** with the numbering scheme of molecular structure. Atomic displacement ellipsoids are drawn at the 50% probability level.

6. NMR spectra

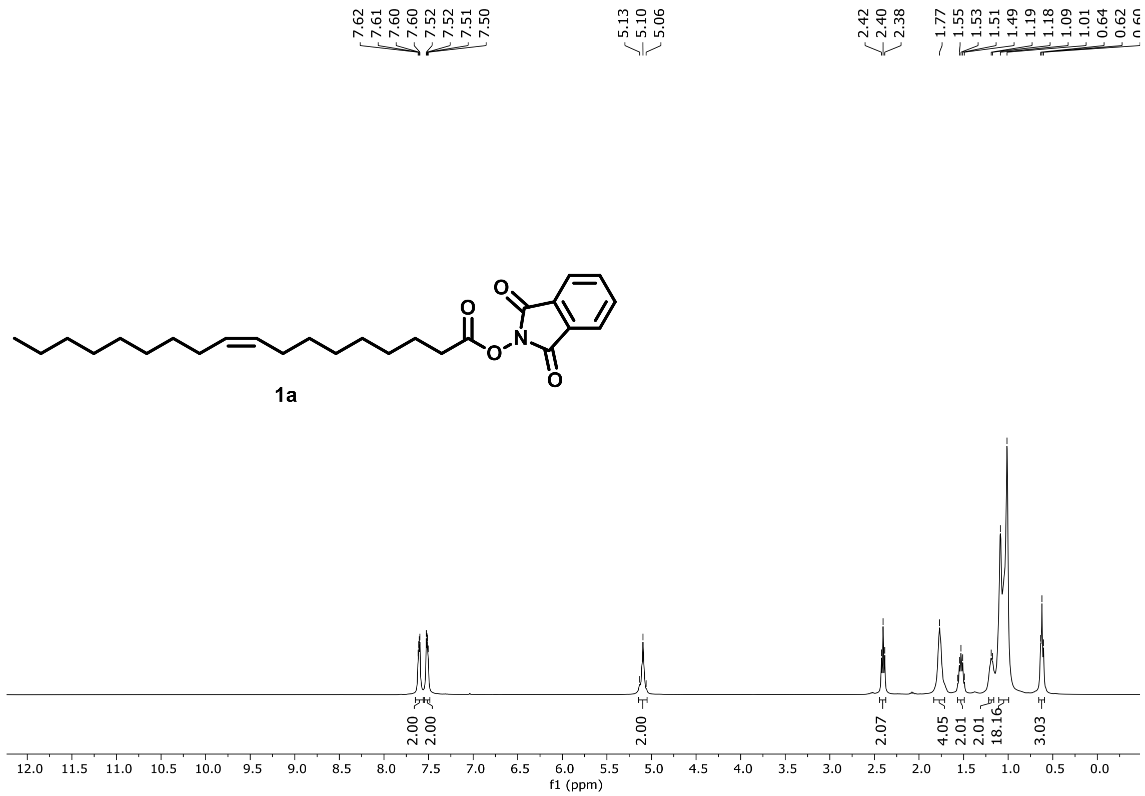

Figure S9: ^{1}H NMR spectrum of phthalimide **1a** (400 MHz, $CDCl_3$)

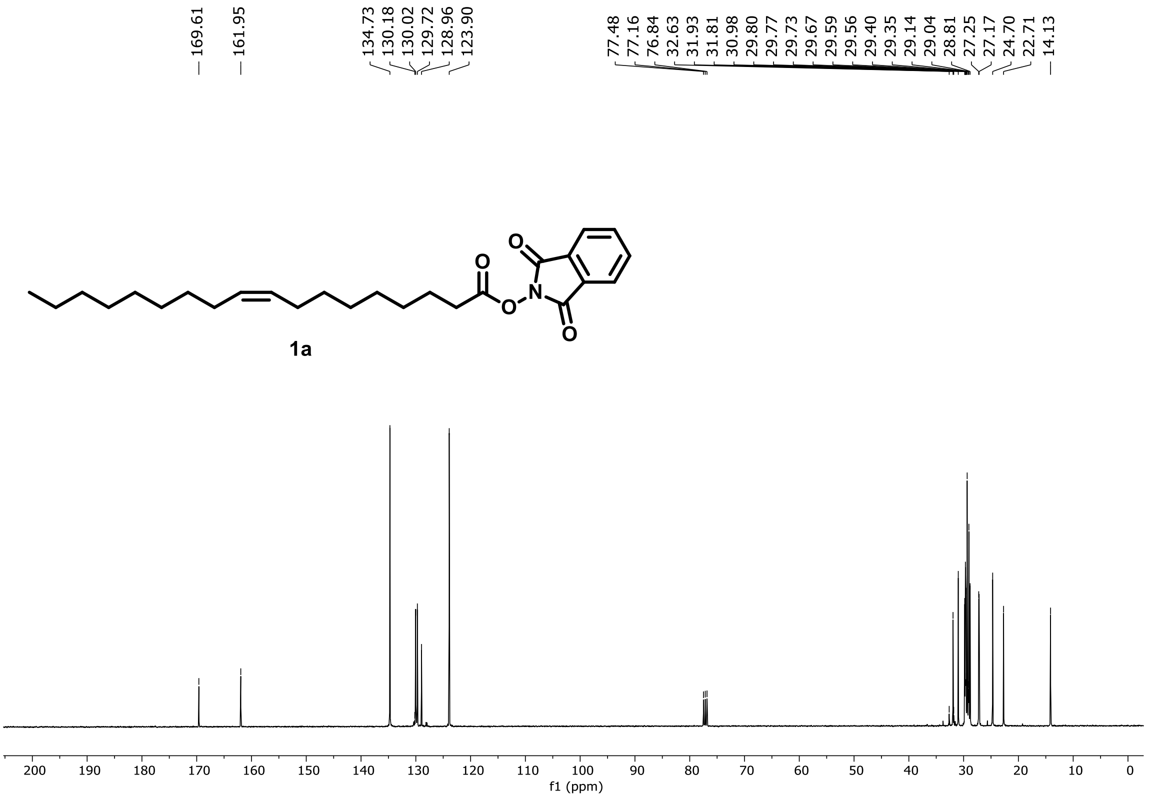

Figure S10: ^{13}C NMR spectrum of phthalimide **1a** (101 MHz, $CDCl_3$)

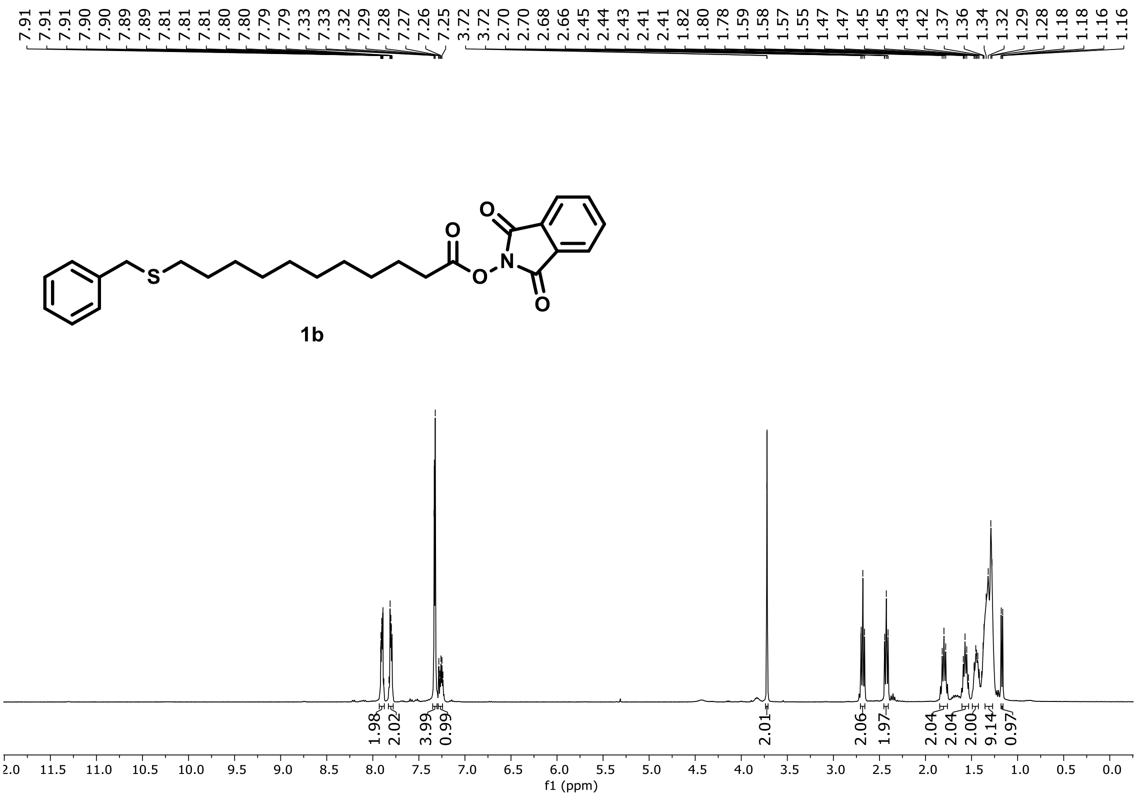

Figure S11: ^{1}H NMR spectrum of phthalimide **1b** (400 MHz, $CDCl_3$)

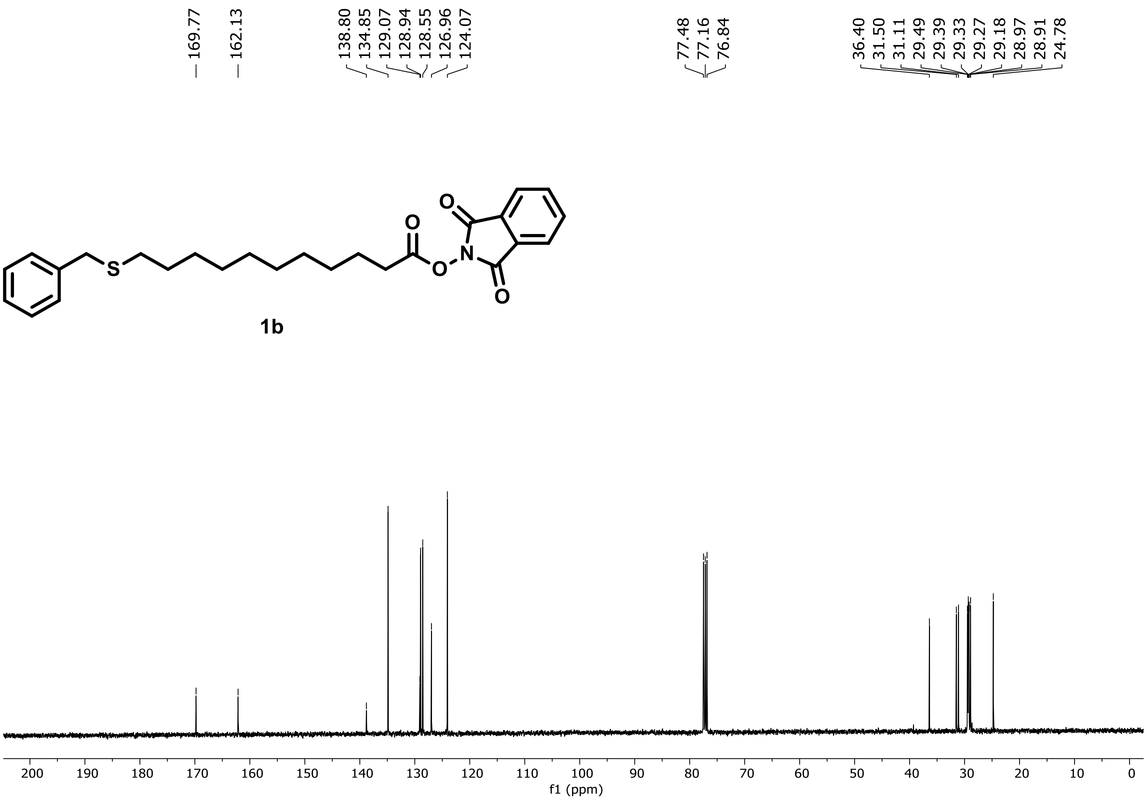

Figure S12: ^{13}C NMR spectrum of phthalimide **1b** (101 MHz, $CDCl_3$)

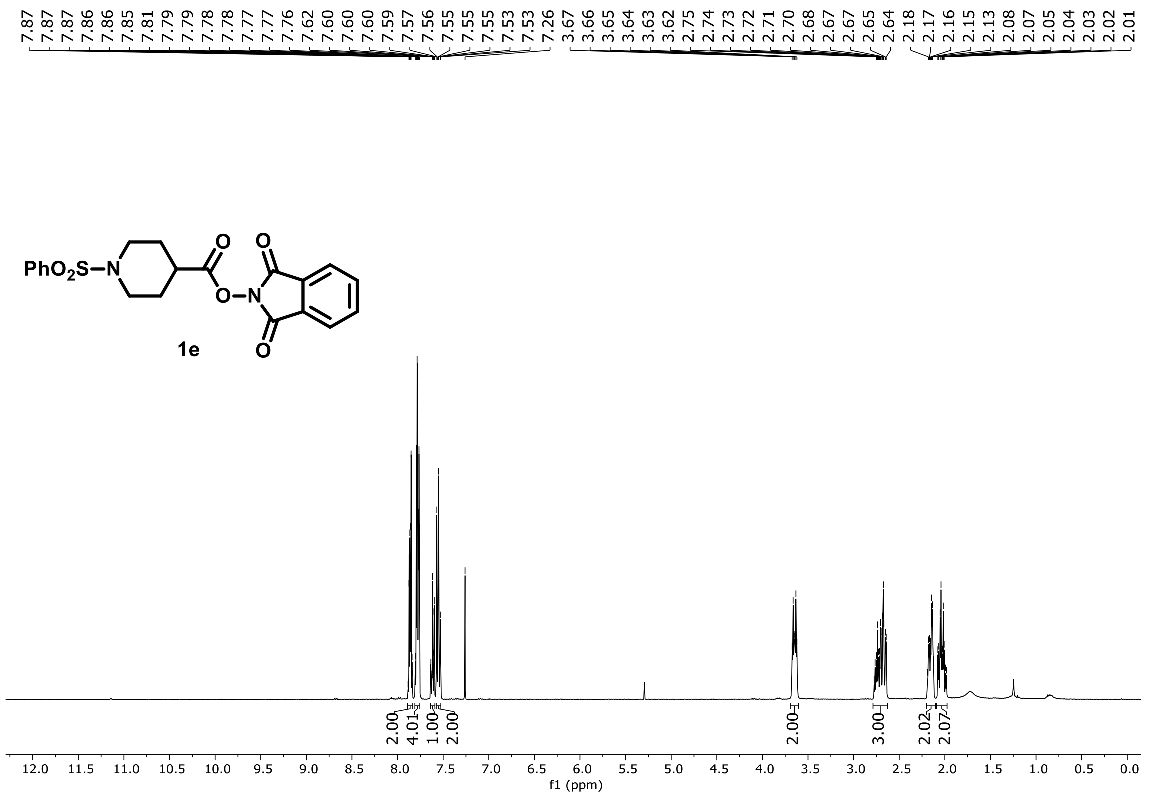

Figure S13: ^{1}H NMR spectrum of phthalimide **1e** (400 MHz, $CDCl_3$)

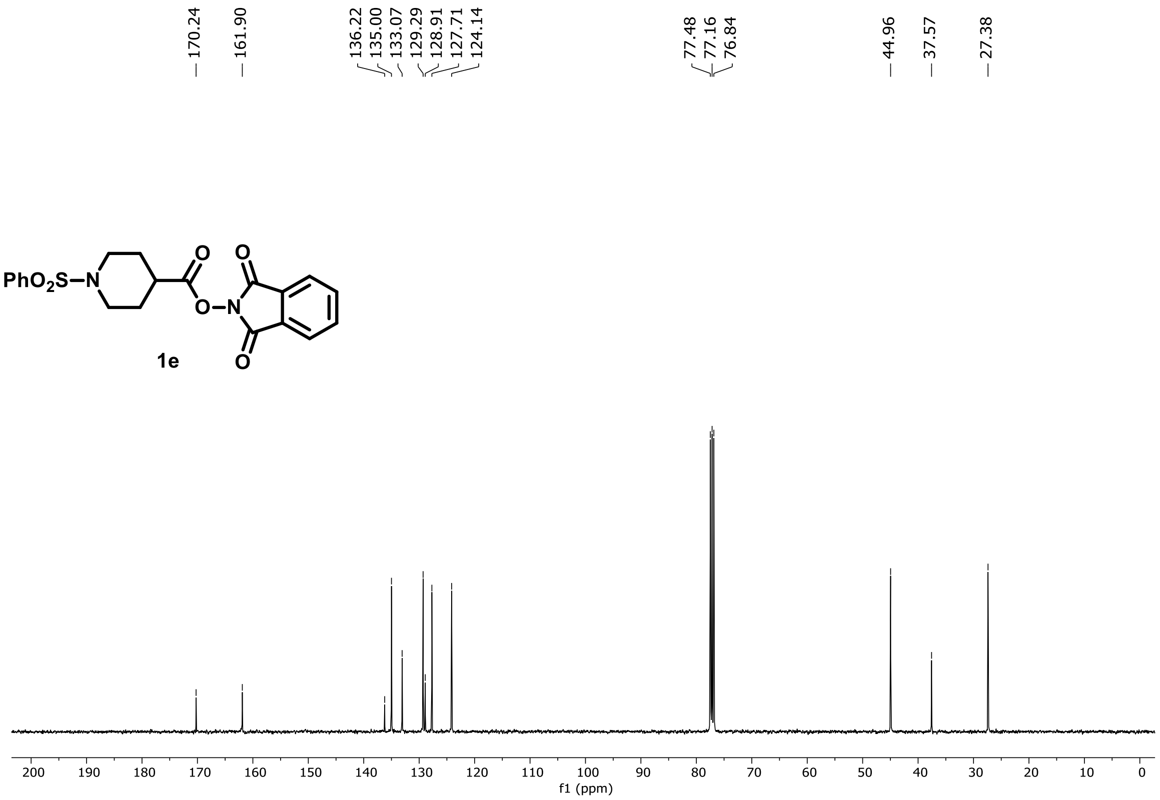

Figure S14: ^{13}C NMR spectrum of phthalimide **1e** (101 MHz, $CDCl_3$)

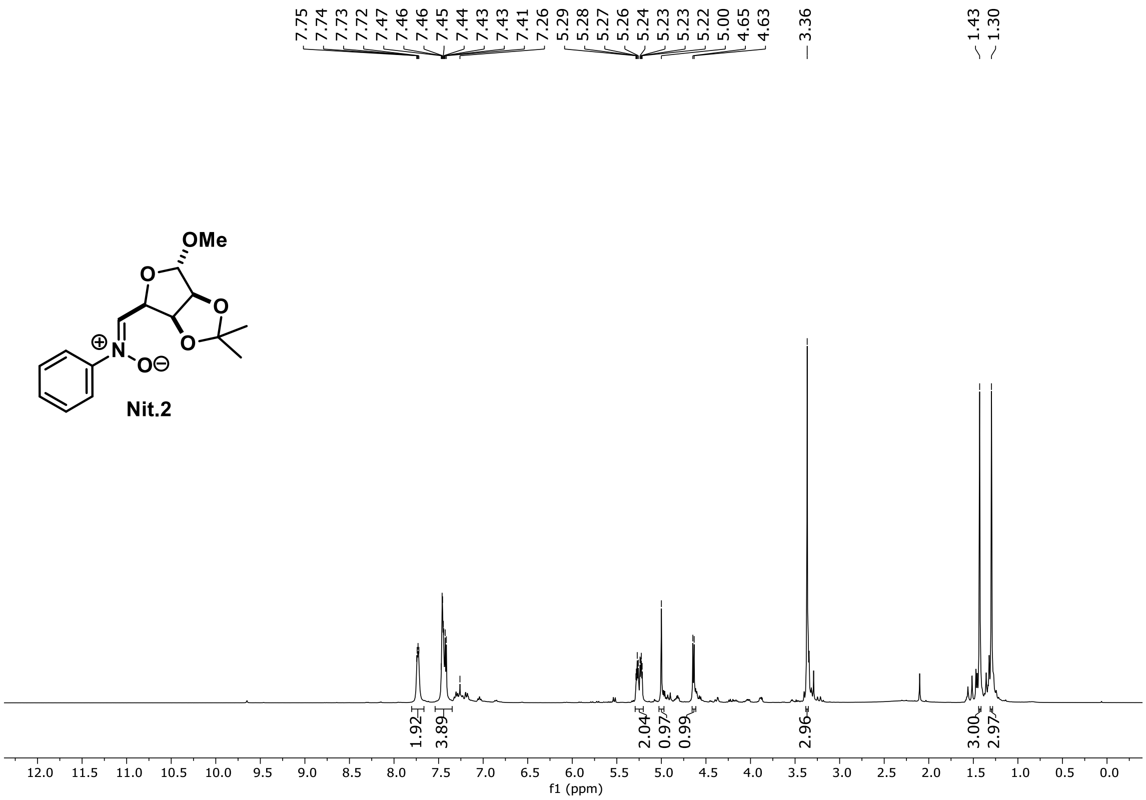

Figure S15: ^{1}H NMR spectrum of **Nit.2** (400 MHz, $CDCl_3$)

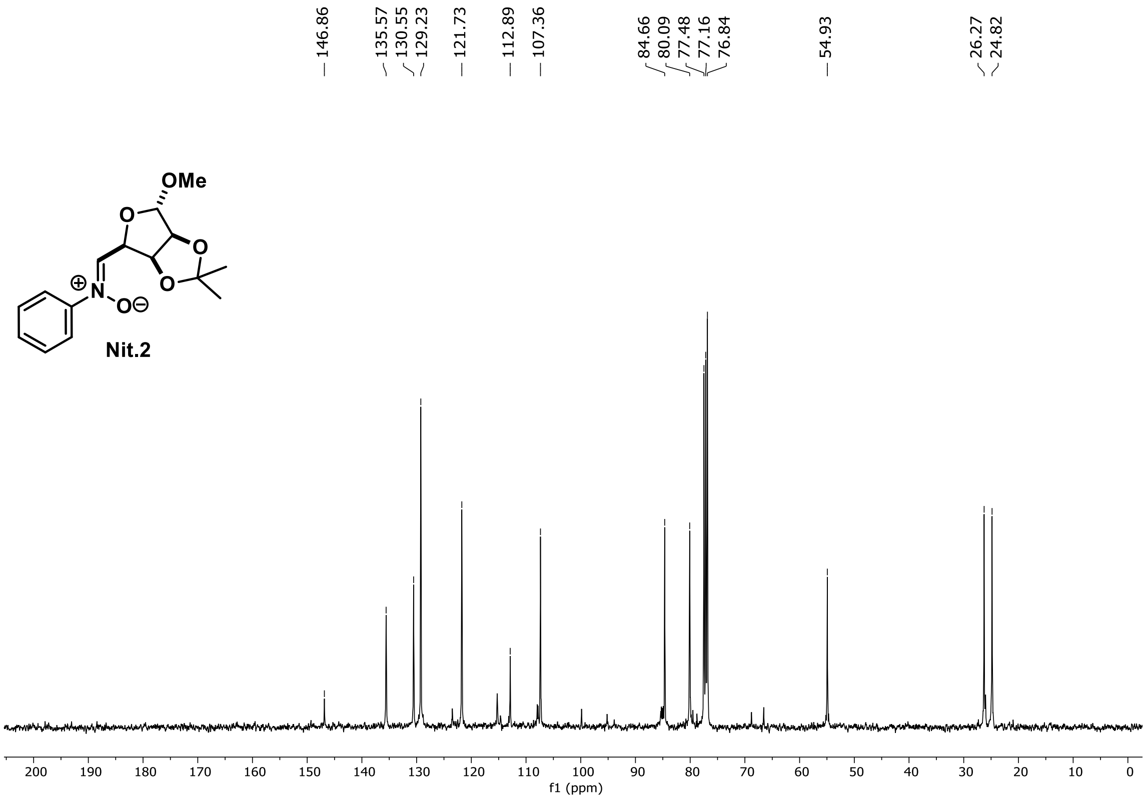

Figure S16: ^{13}C NMR spectrum of **Nit.2** (101 MHz, $CDCl_3$)

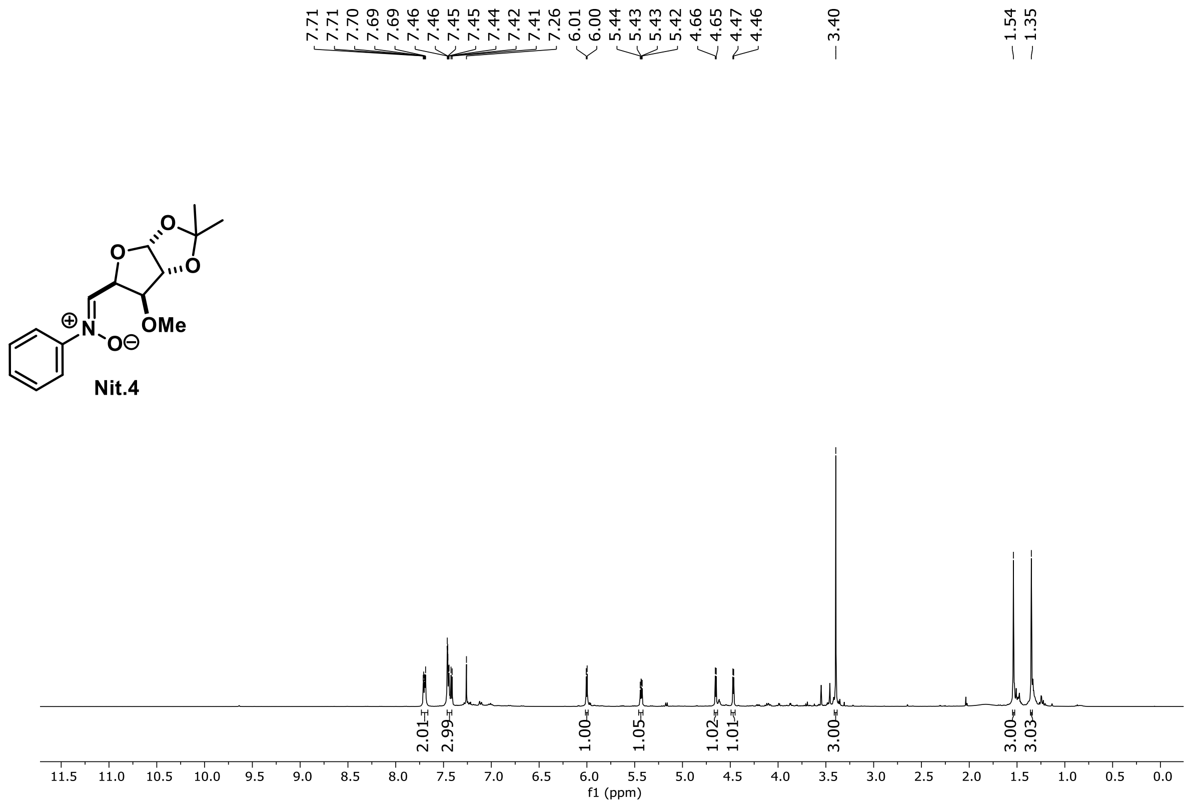

Figure S17: ^{1}H NMR spectrum of **Nit.4** (400 MHz, $CDCl_3$)

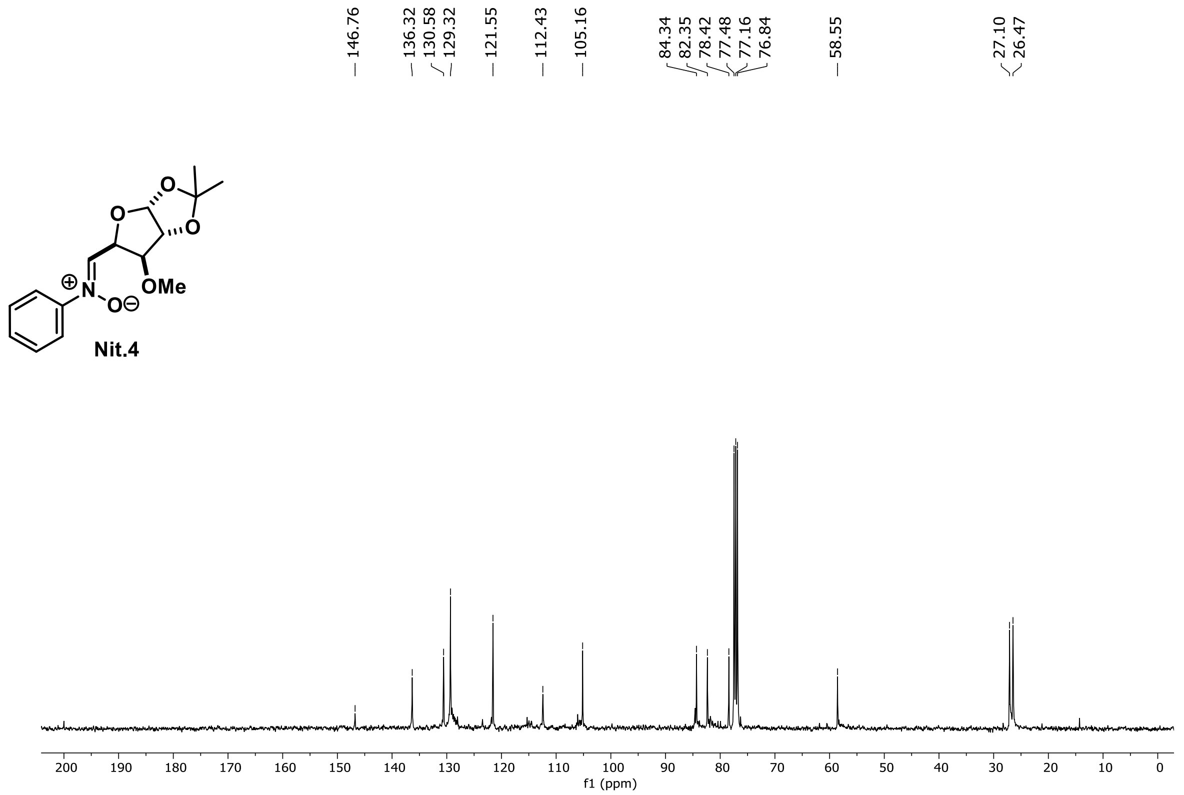

Figure S18: ^{13}C NMR spectrum of **Nit.4** (101 MHz, $CDCl_3$)

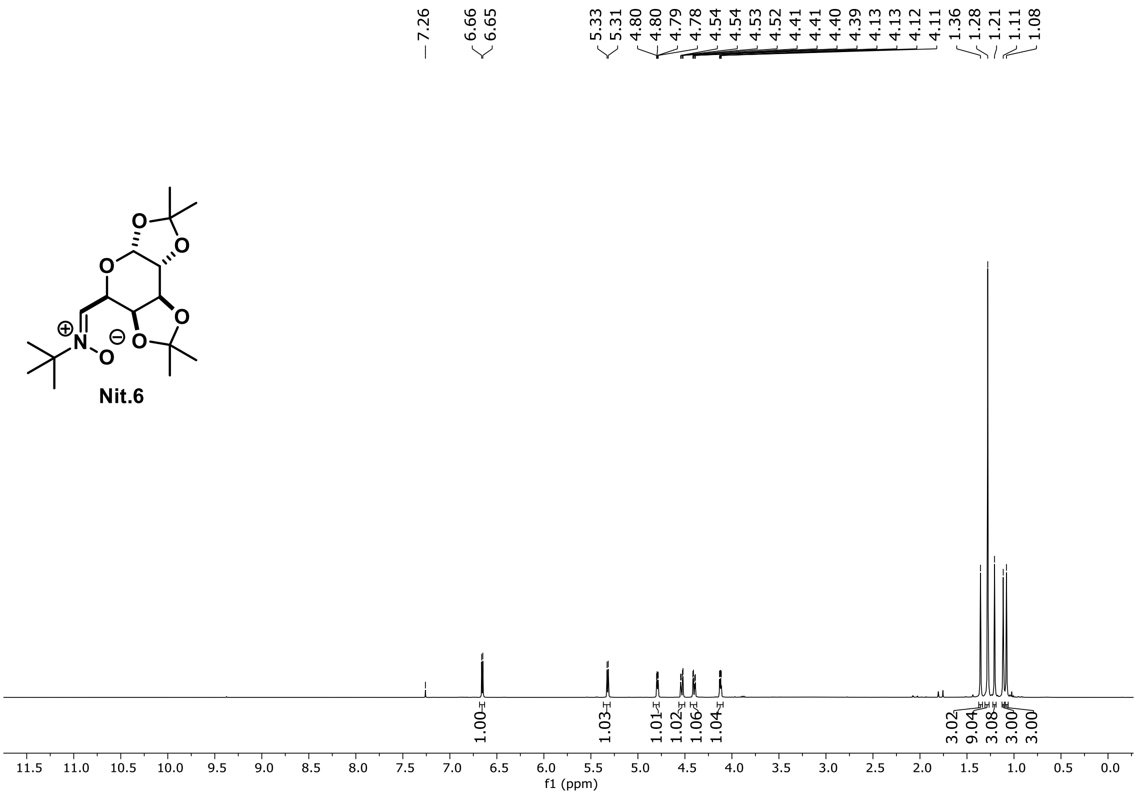

Figure S19: ^{1}H NMR spectrum of **Nit.6** (400 MHz, $CDCl_3$)

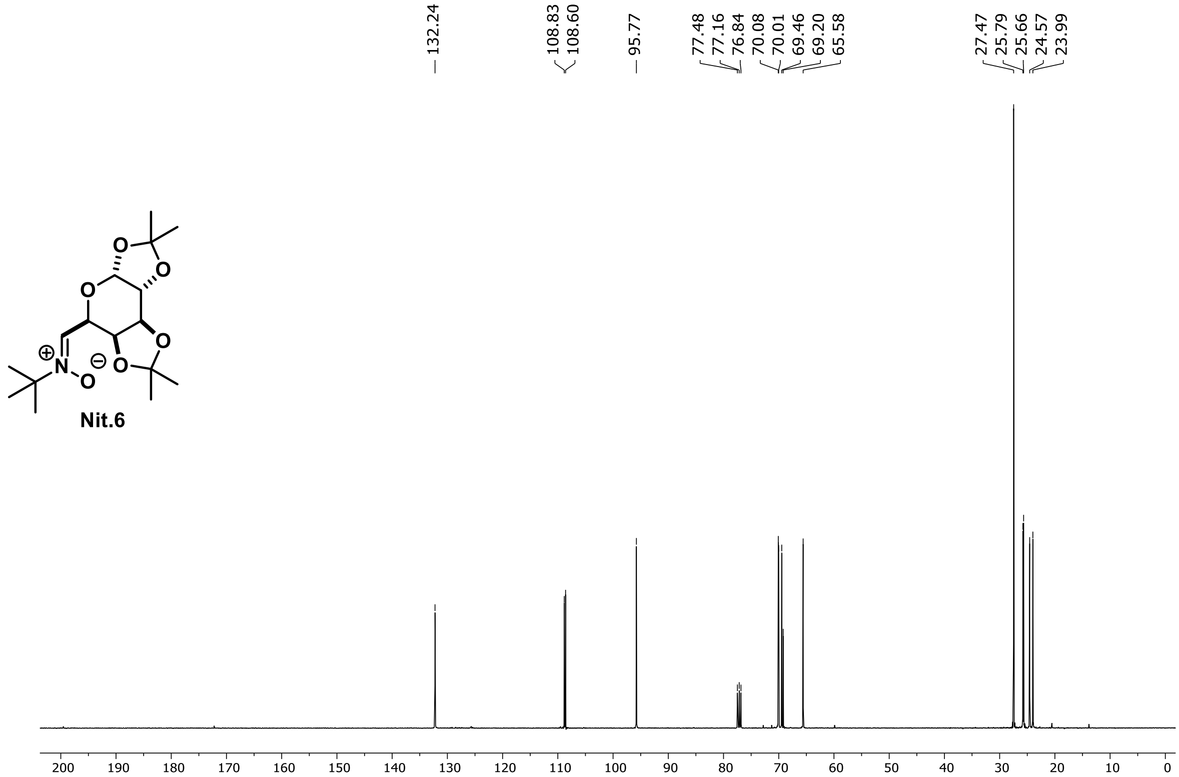

Figure S20: ^{13}C NMR spectrum of **Nit.6** (101 MHz, $CDCl_3$)

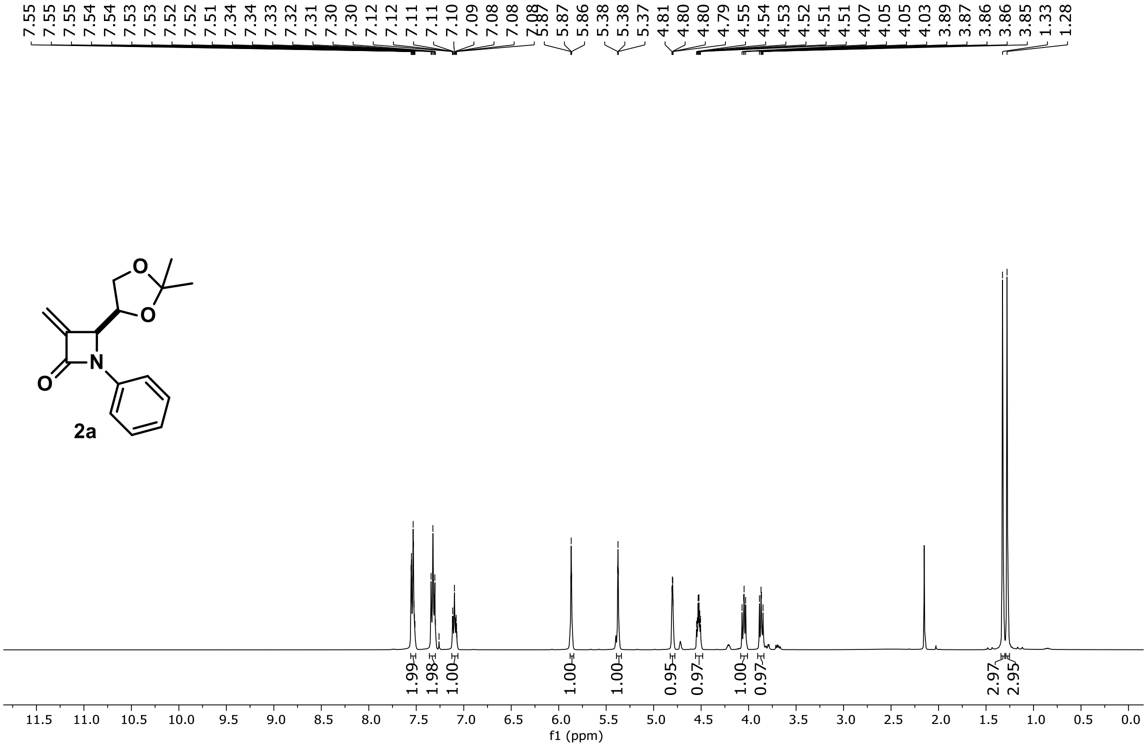

Figure S21: ^{1}H NMR spectrum of **2a** (400 MHz, $CDCl_3$)

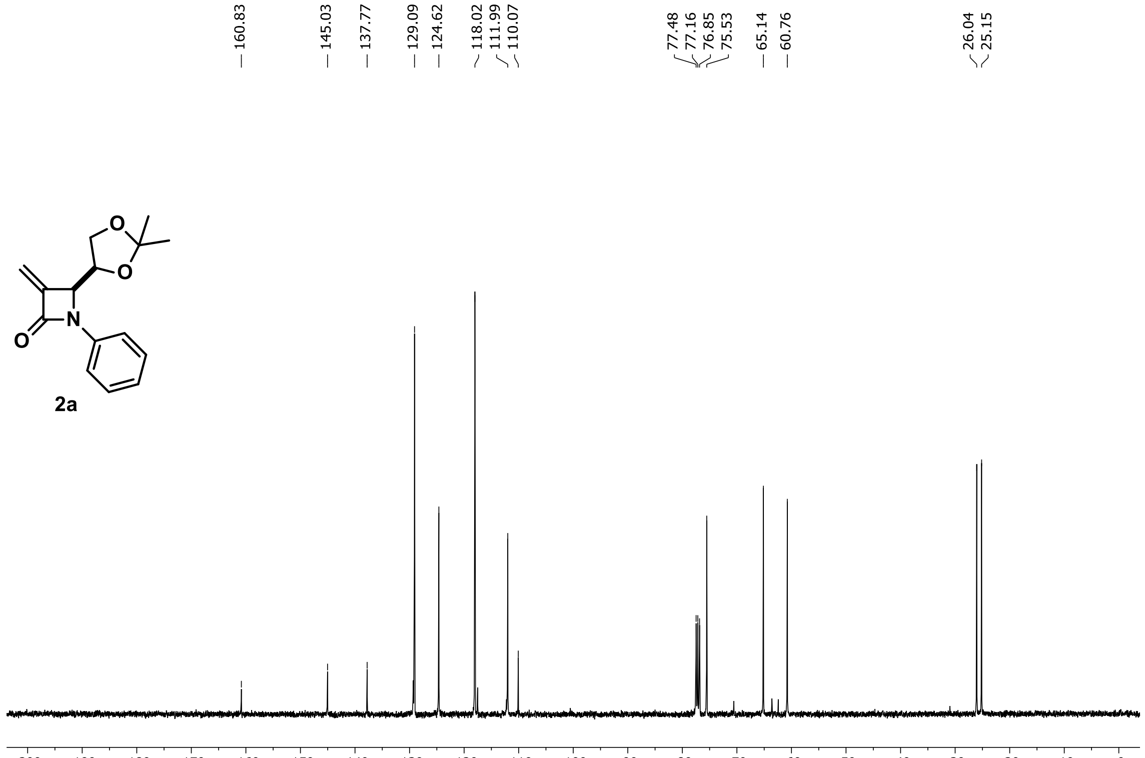

Figure S22: ^{13}C NMR spectrum of **2a** (101 MHz, $CDCl_3$)

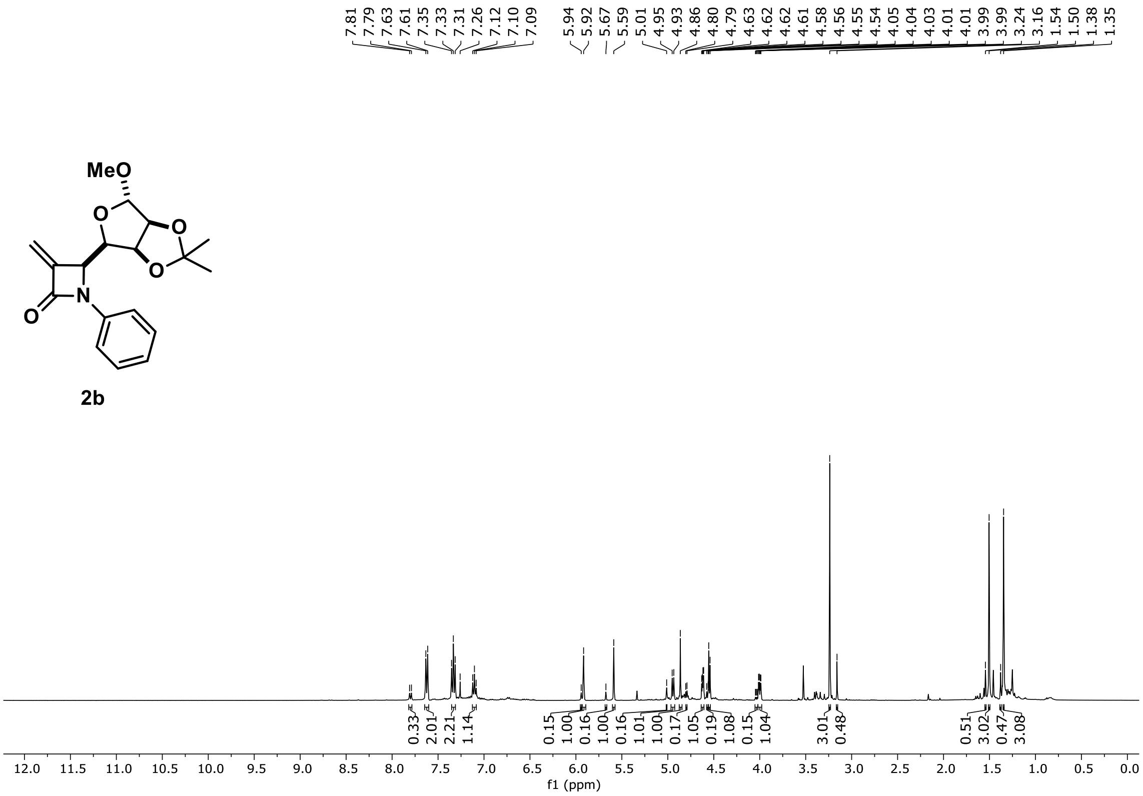

Figure S23: ^{1}H NMR spectrum of **2b** (400 MHz, $CDCl_3$)

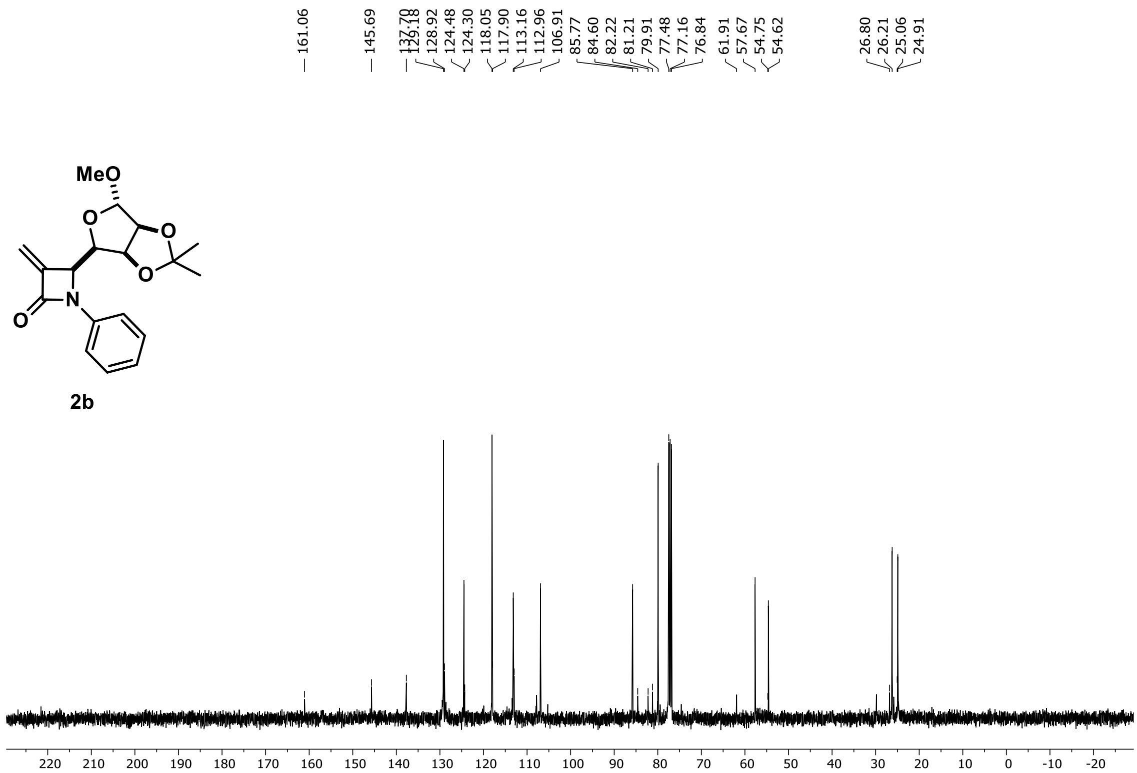

Figure S24: ^{13}C NMR spectrum of **2b** (101 MHz, $CDCl_3$)

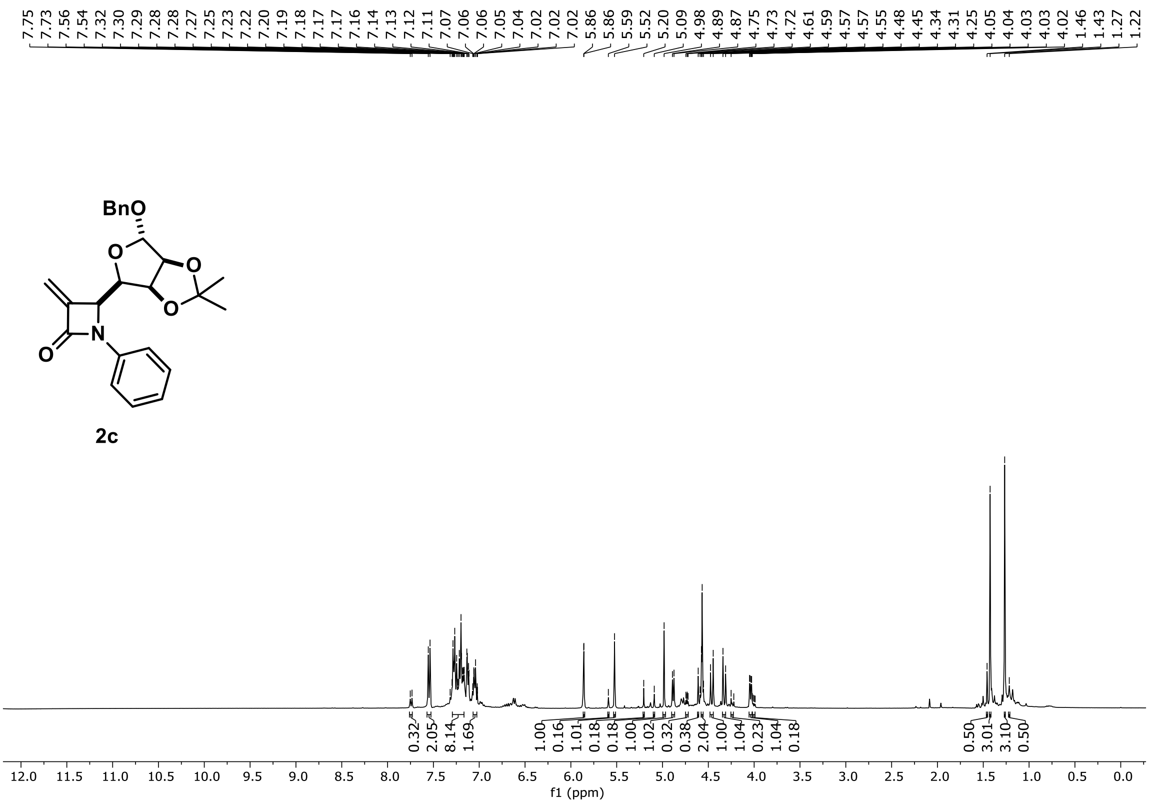

Figure S25: ^{1}H NMR spectrum of **2c** (400 MHz, $CDCl_3$)

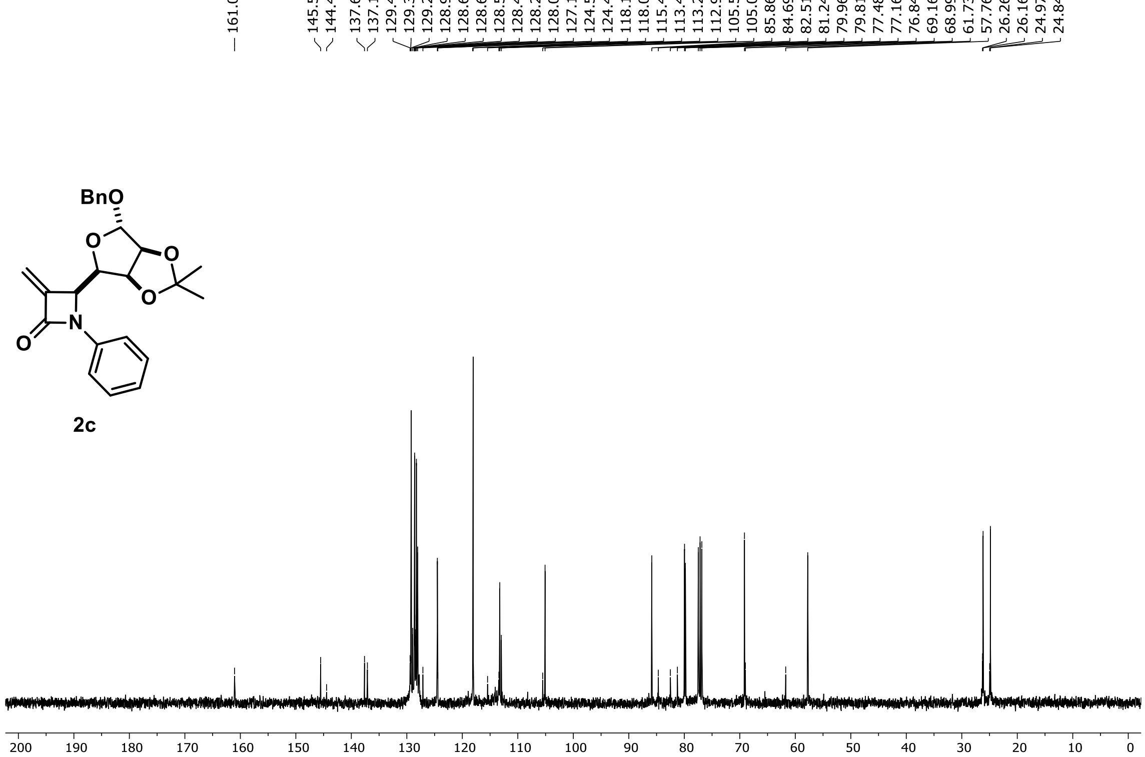

Figure S26: ^{13}C NMR spectrum of **2c** (101 MHz, $CDCl_3$)

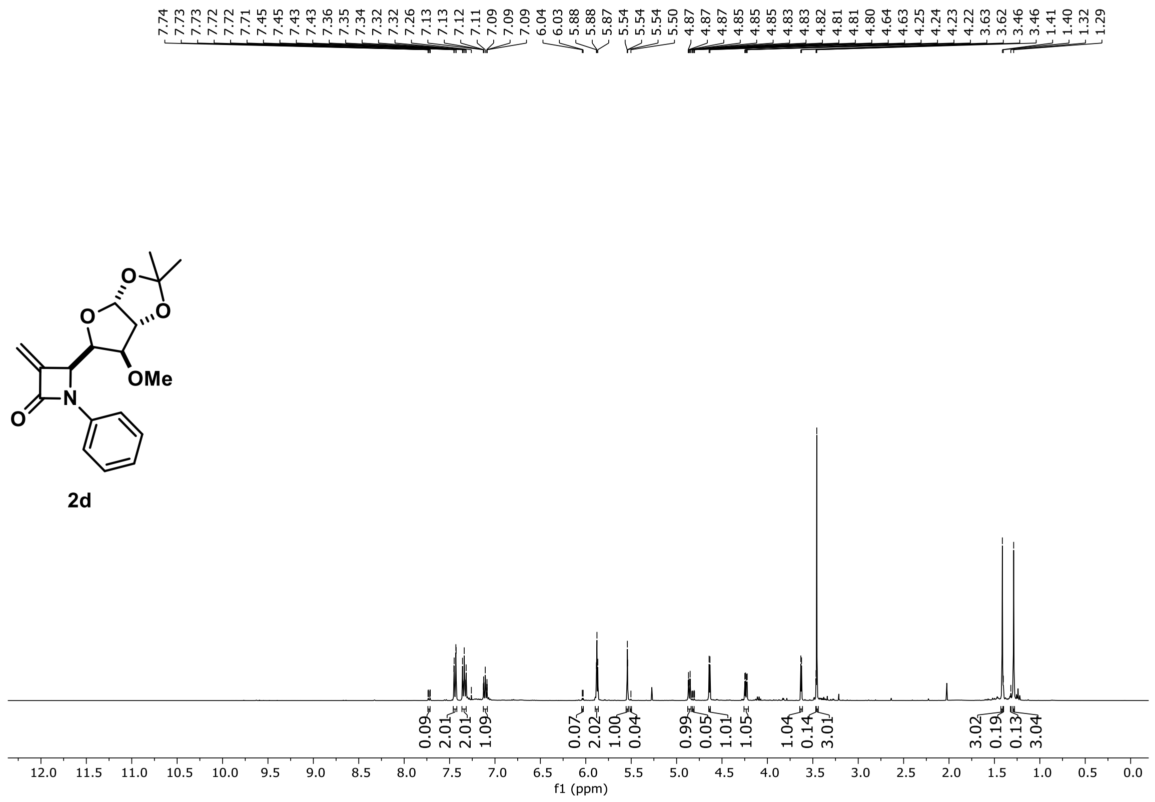

Figure S27: ^{1}H NMR spectrum of **2d** (400 MHz, $CDCl_3$)

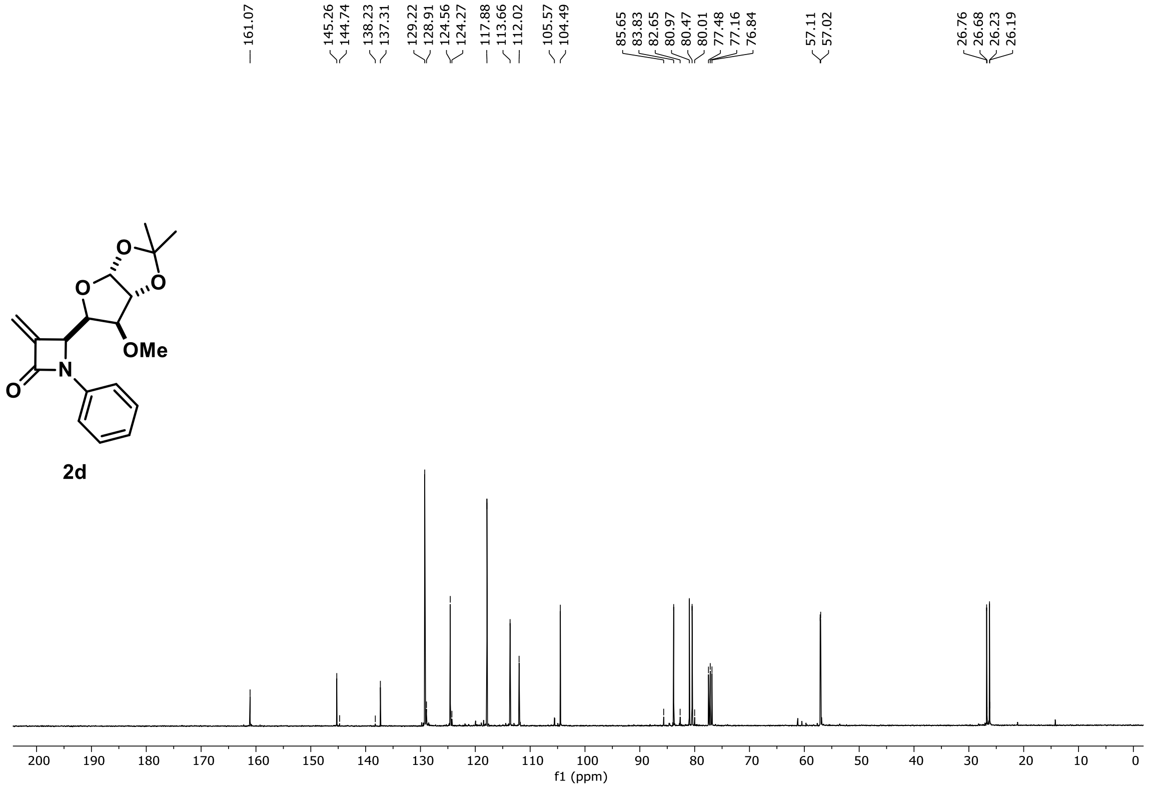

Figure S28: ^{13}C NMR spectrum of **2d** (101 MHz, $CDCl_3$)

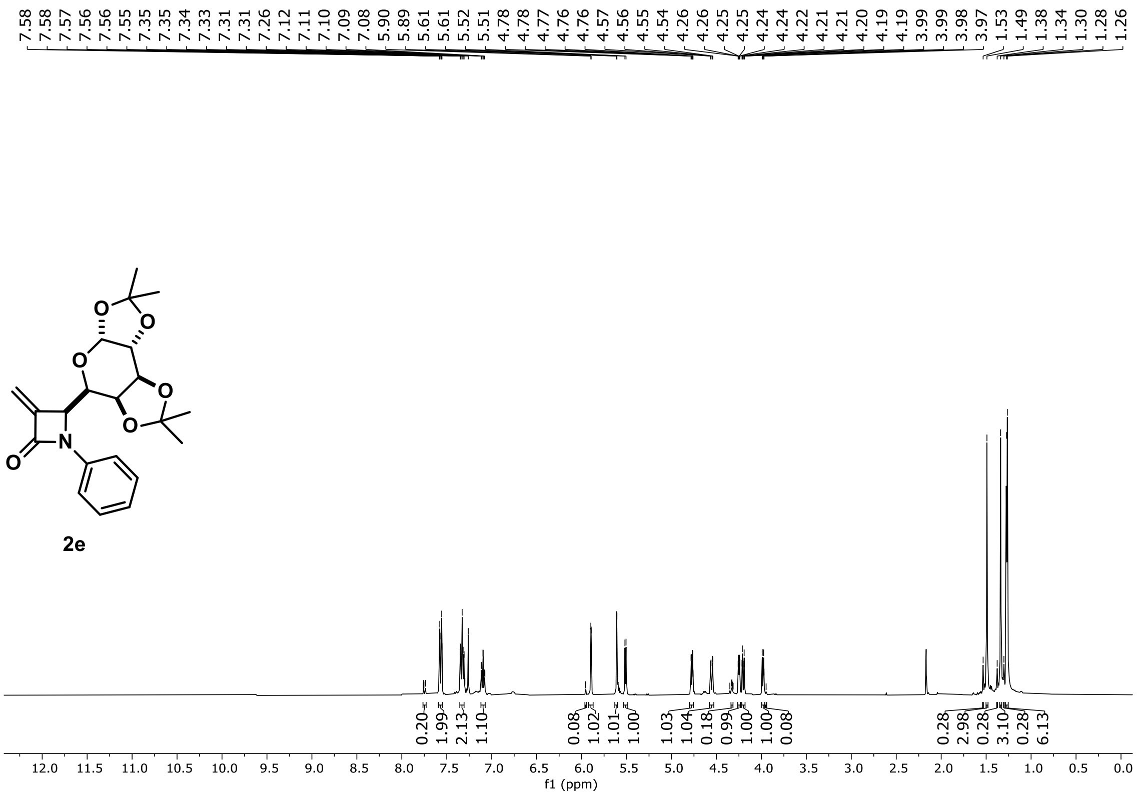

Figure S29: ^{1}H NMR spectrum of **2e** (400 MHz, $CDCl_3$)

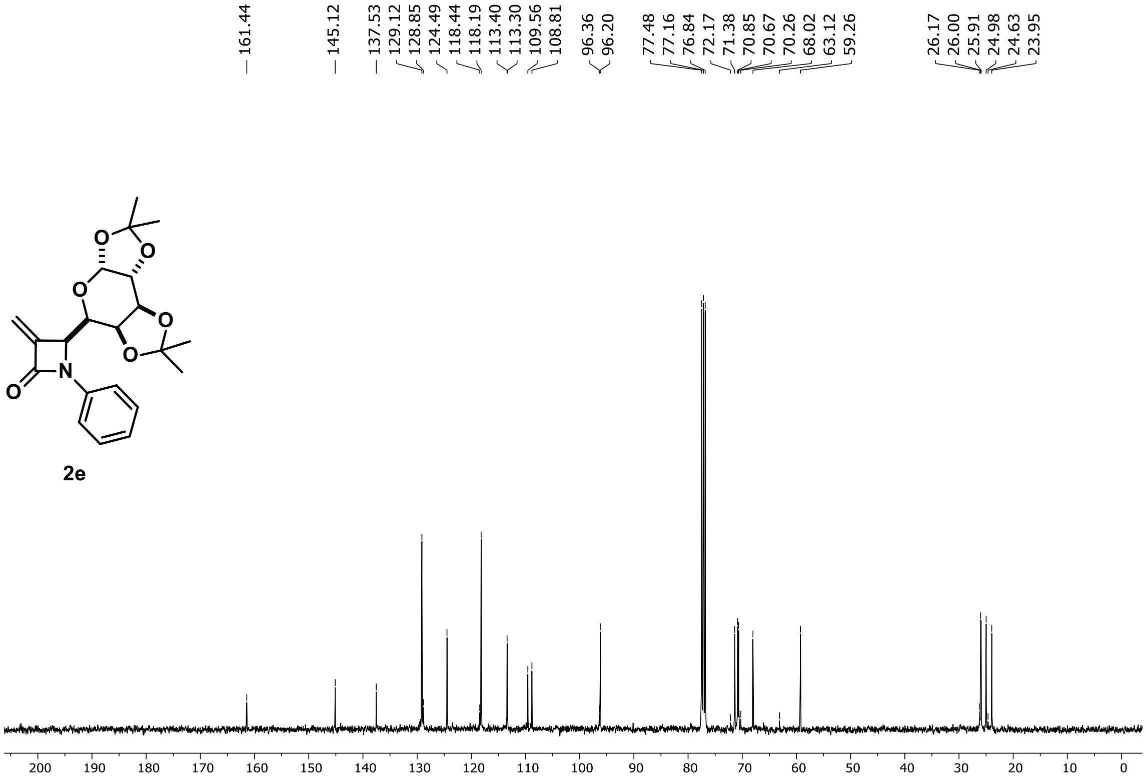

Figure S30: ^{13}C NMR spectrum of **2e** (101 MHz, $CDCl_3$)

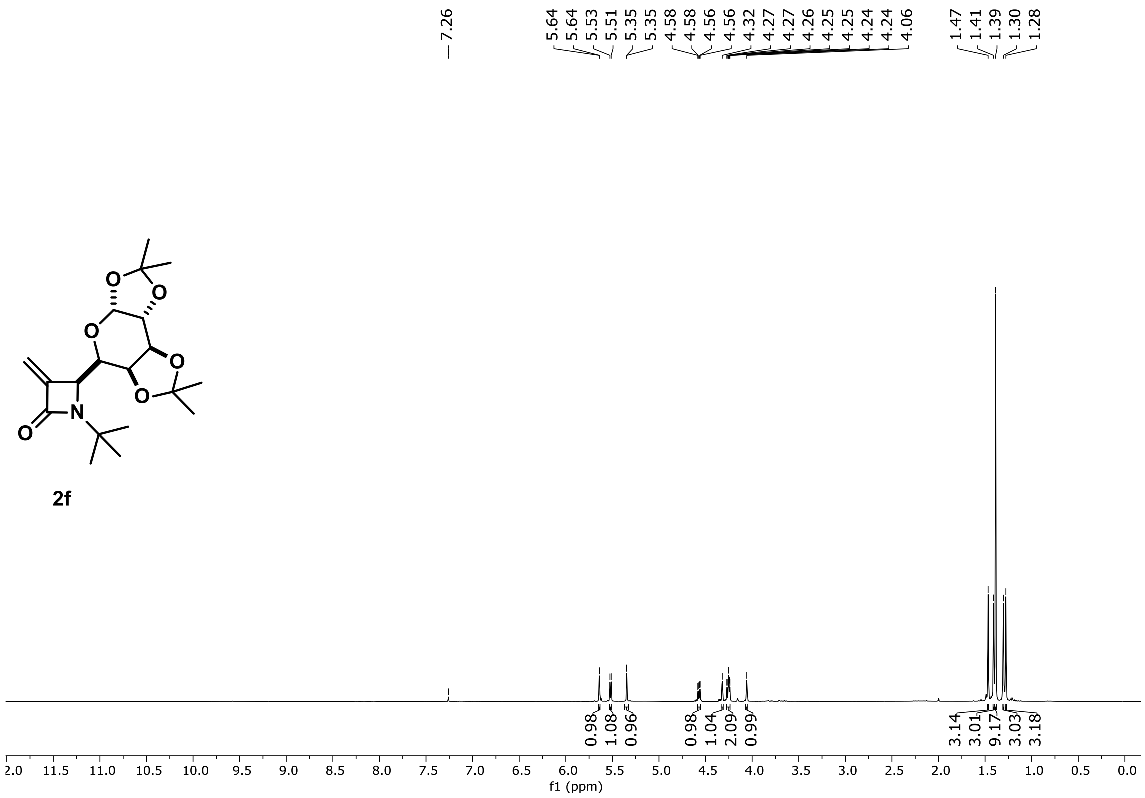

Figure S31: ^{1}H NMR spectrum of **2f** (400 MHz, $CDCl_3$)

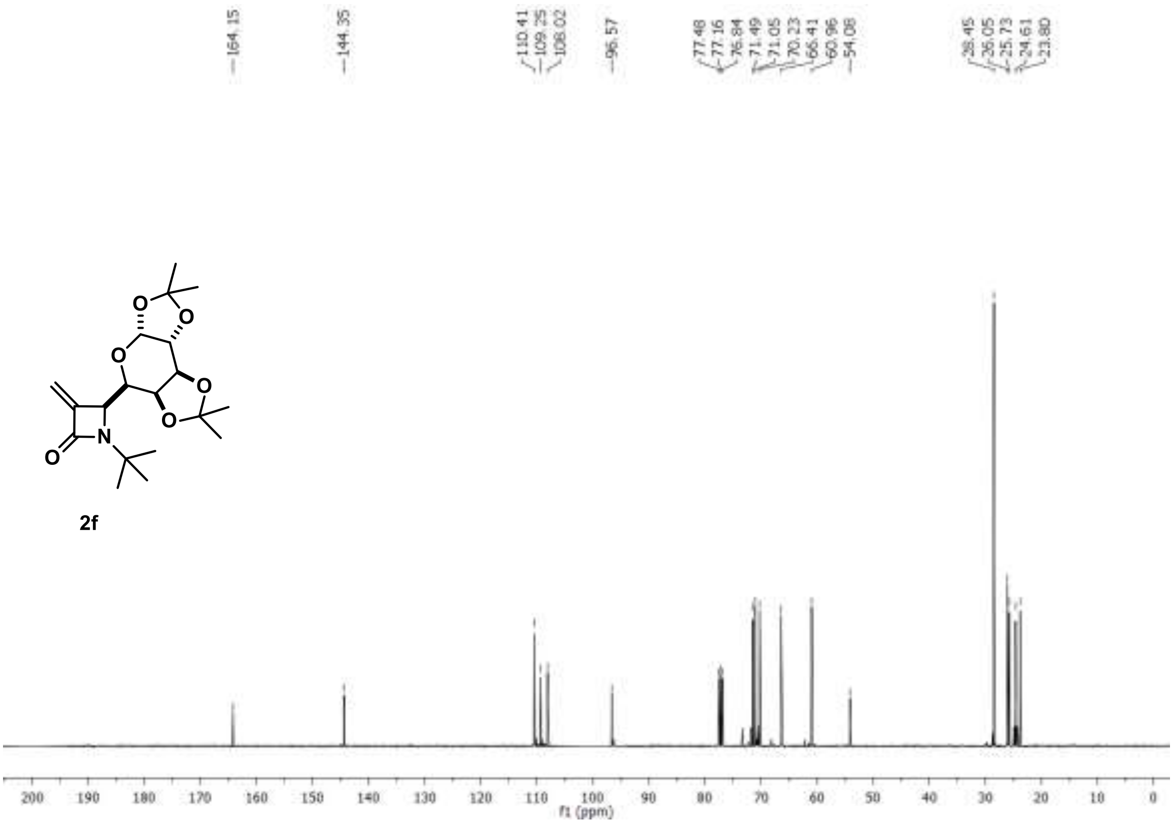

Figure S32: ^{13}C NMR spectrum of **2f** (101 MHz, $CDCl_3$)

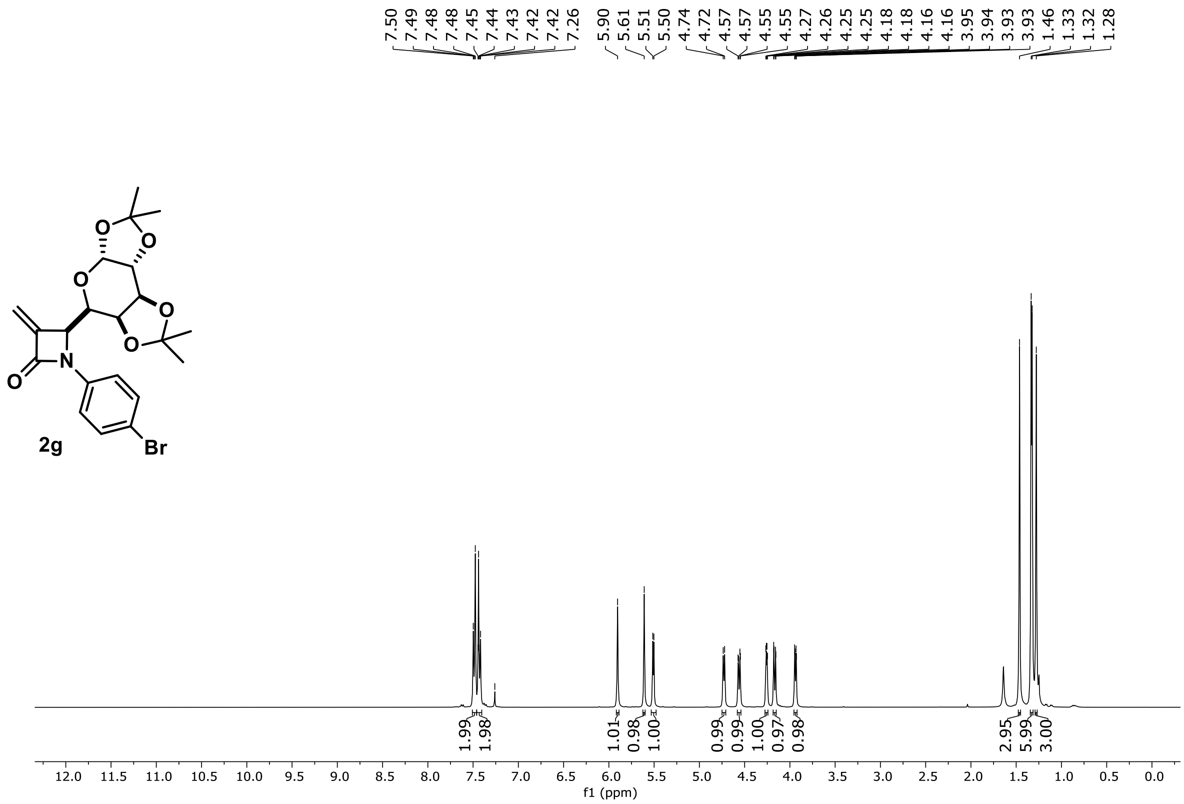

Figure S33: ^{1}H NMR spectrum of **2g** (400 MHz, $CDCl_3$)

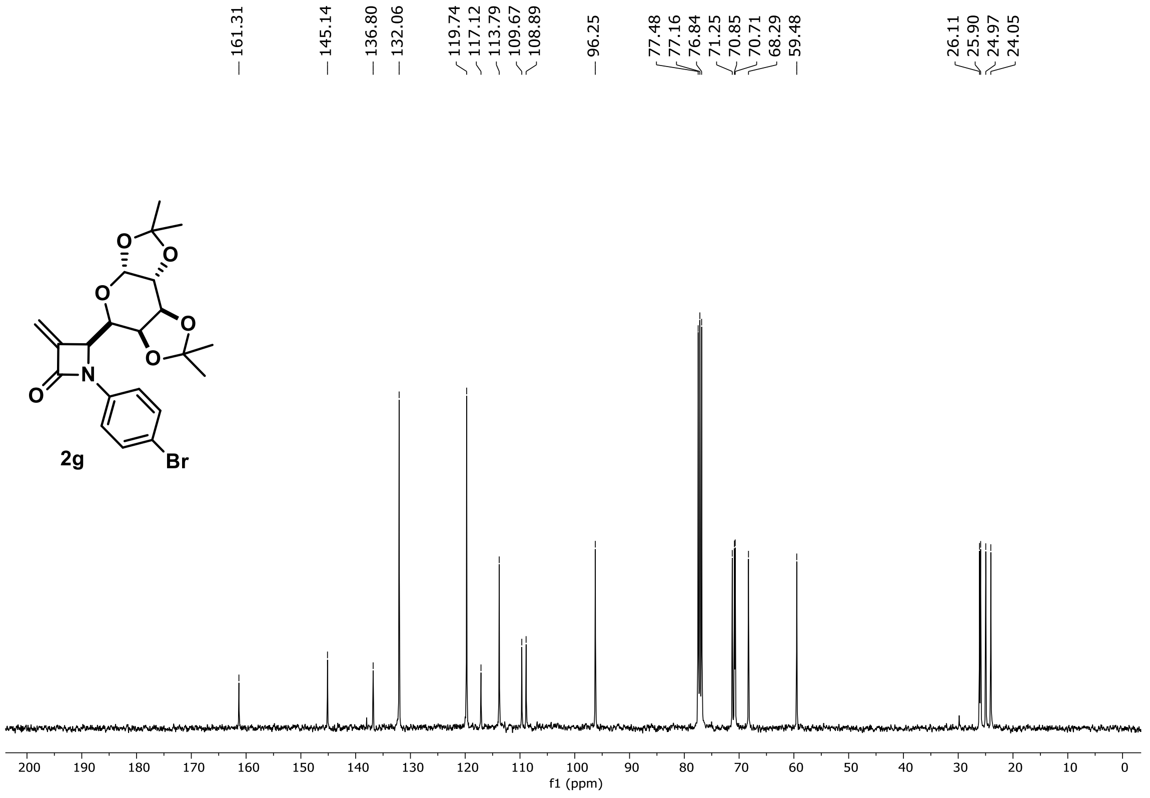

Figure S34: ^{13}C NMR spectrum of **2g** (101 MHz, $CDCl_3$)

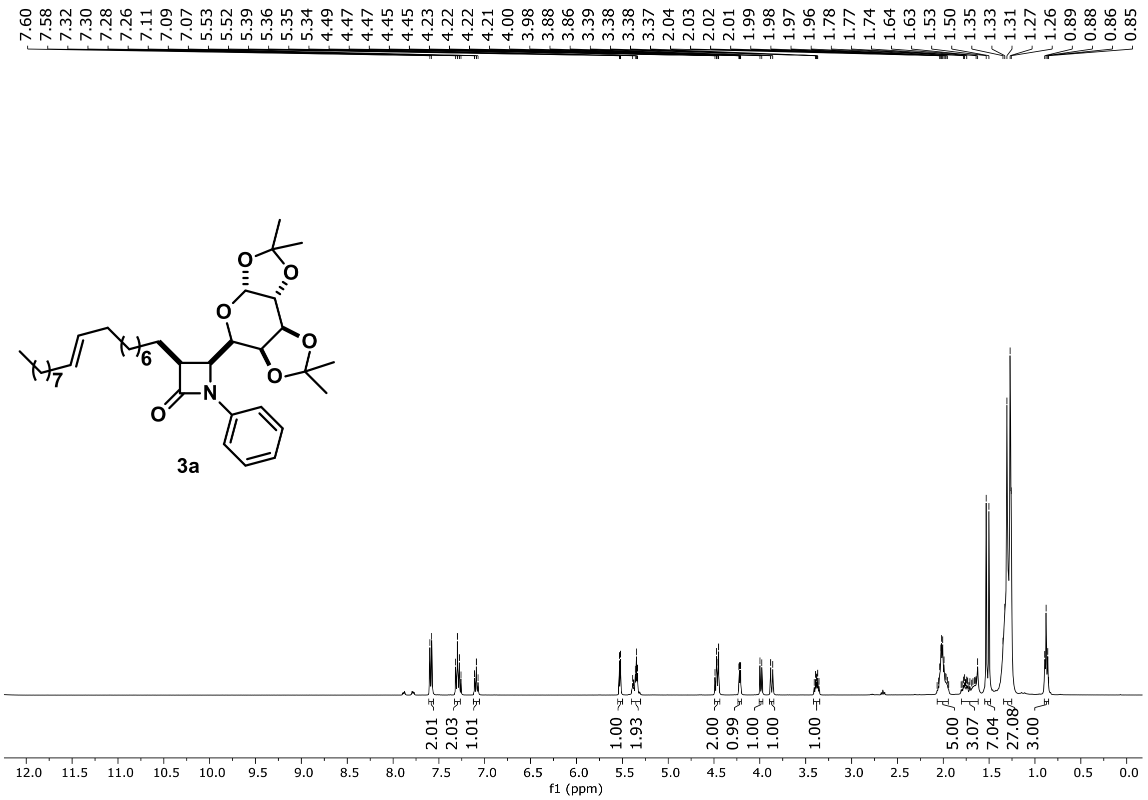

Figure S35: ^{1}H NMR spectrum of product **3a** (400 MHz, $CDCl_3$)

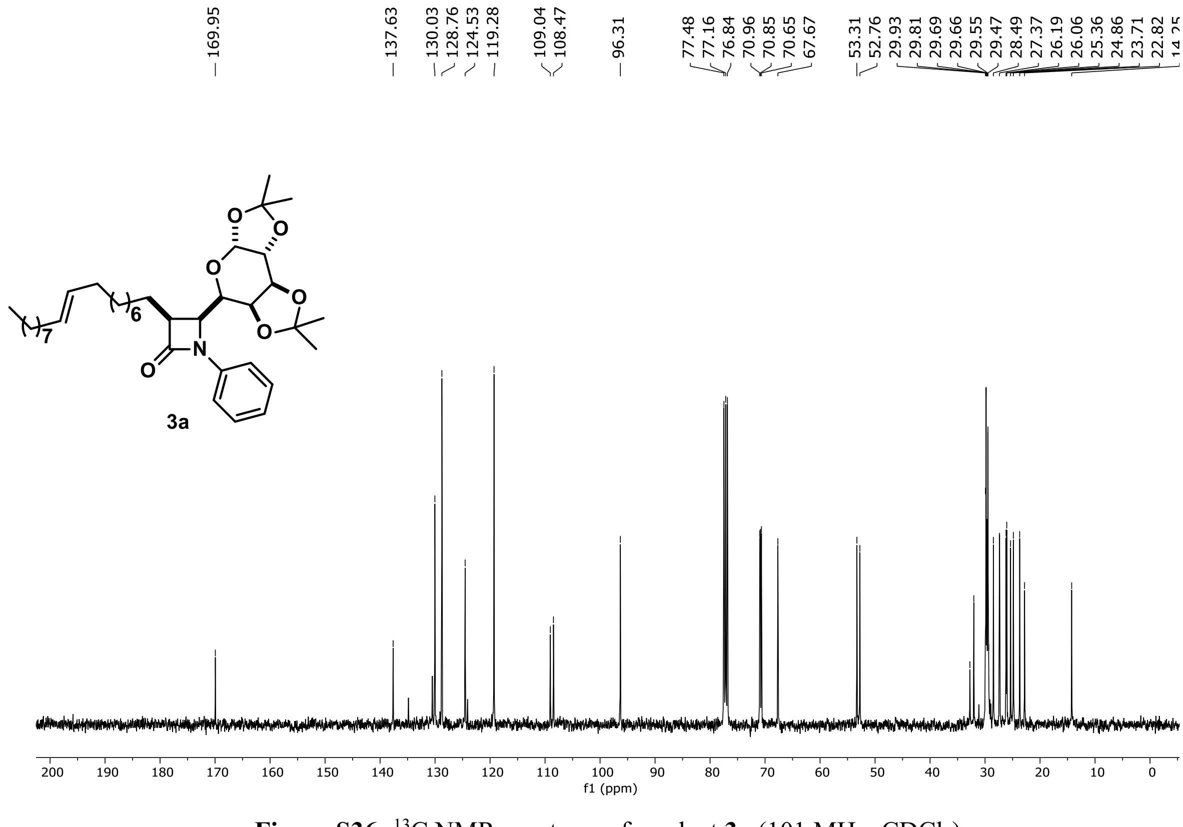

Figure S36: ^{13}C NMR spectrum of product **3a** (101 MHz, $CDCl_3$)

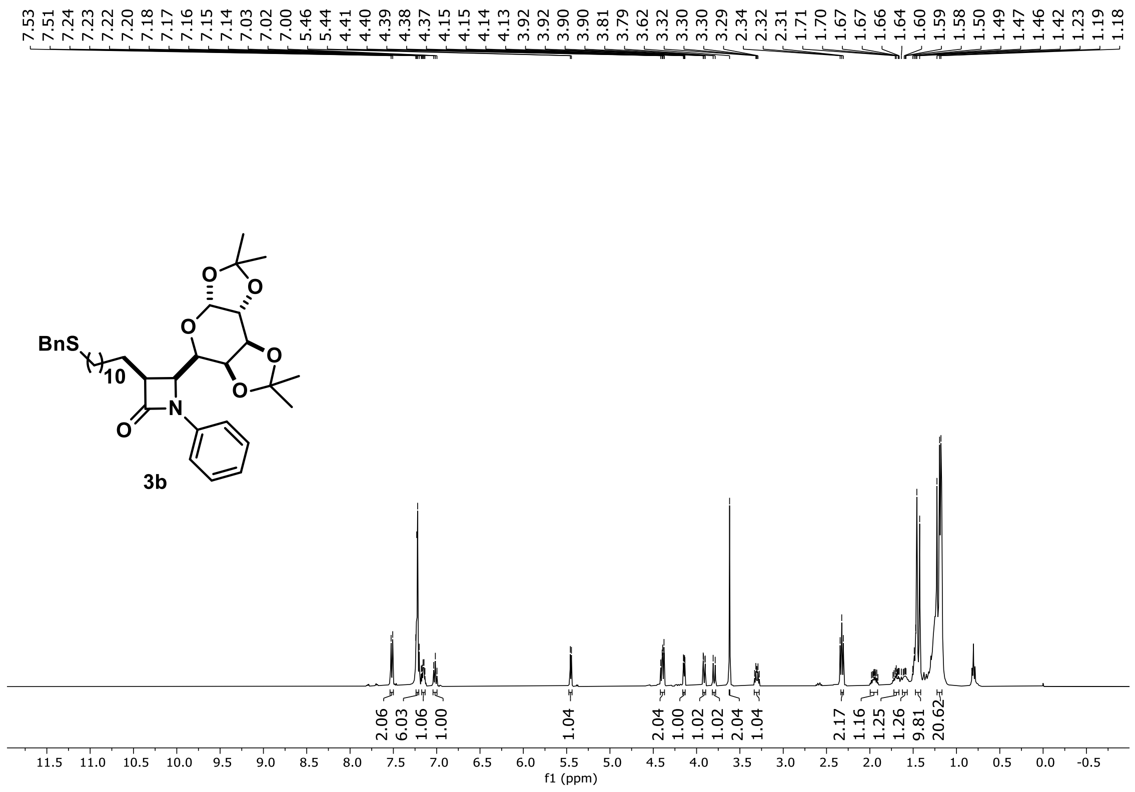

Figure S37: ^{1}H NMR spectrum of product **3b** (400 MHz, $CDCl_3$)

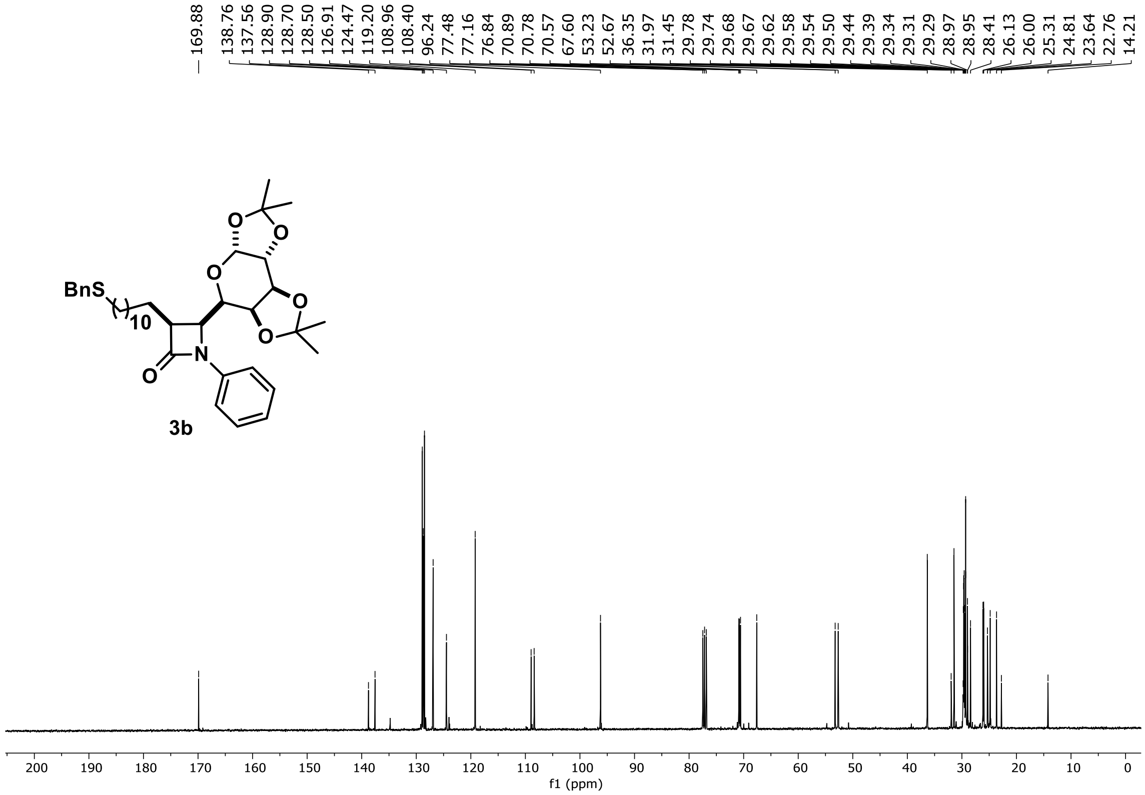

Figure S38: ^{13}C NMR spectrum of product **3b** (101 MHz, $CDCl_3$)

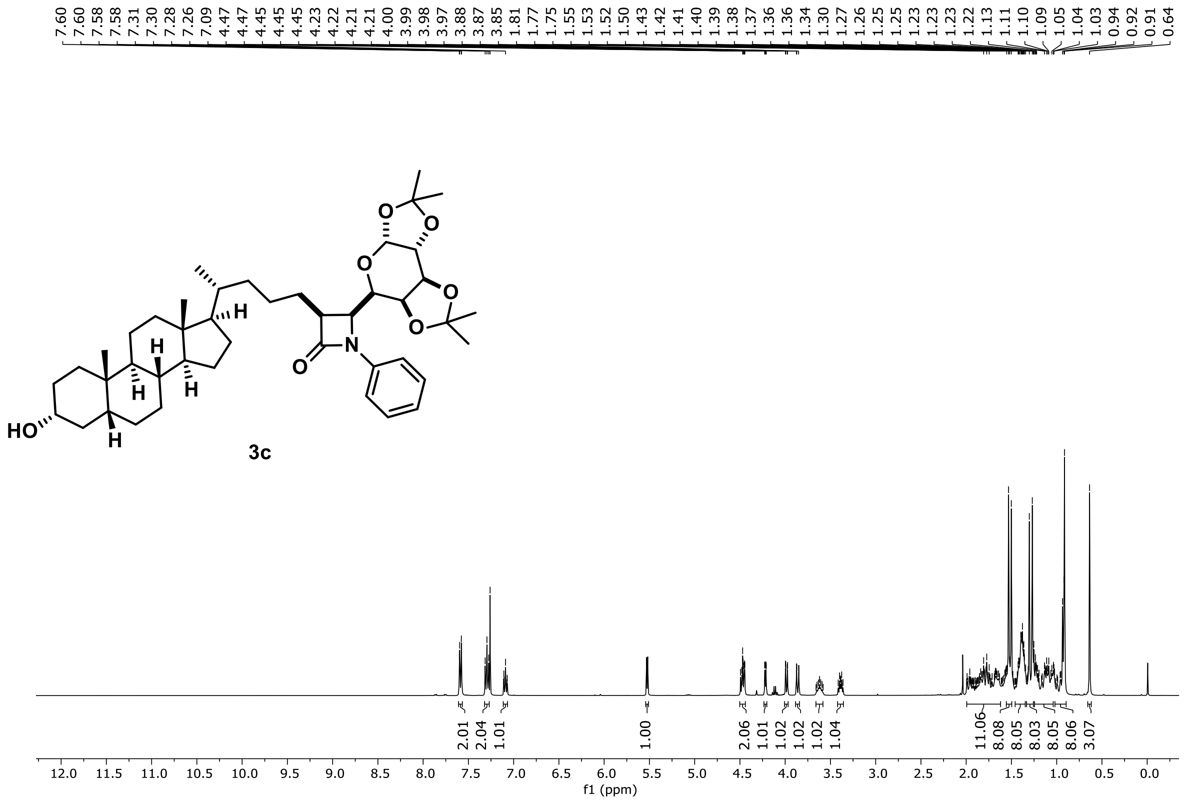

Figure S39: ^{1}H NMR spectrum of product **3c** (400 MHz, $CDCl_3$)

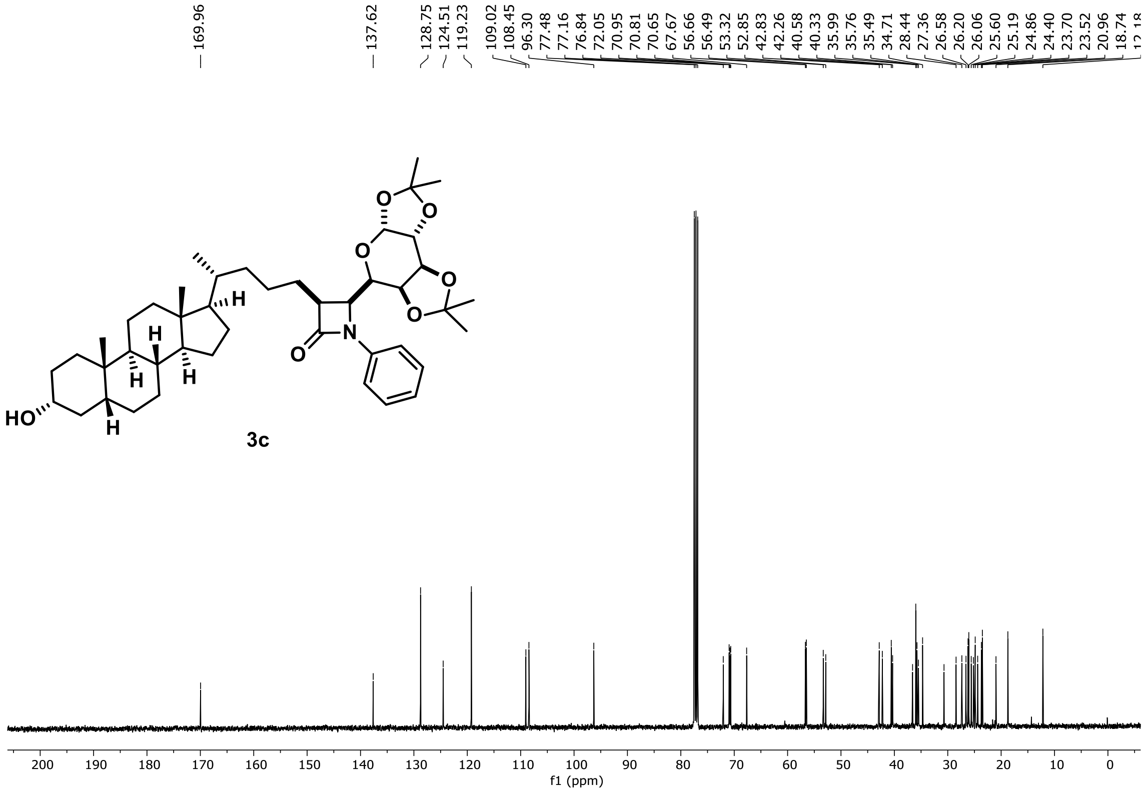

Figure S40: ^{13}C NMR spectrum of product **3c** (101 MHz, $CDCl_3$)

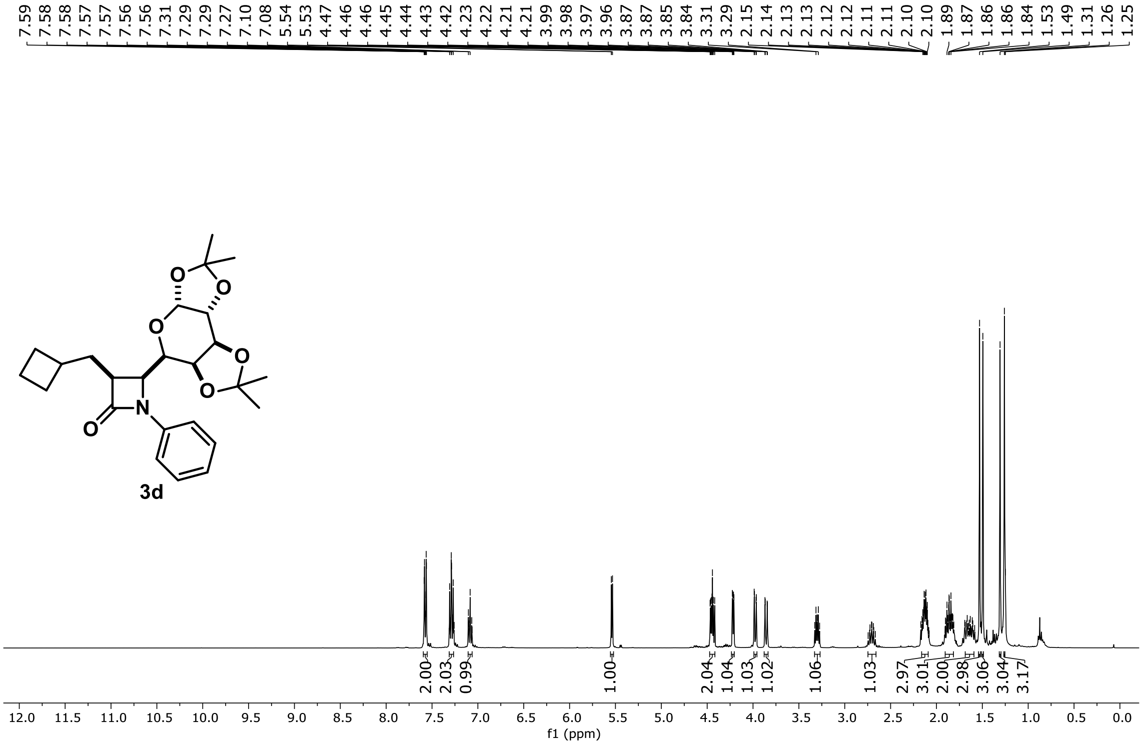

Figure S41: ^{1}H NMR spectrum of product **3d** (400 MHz, $CDCl_3$)

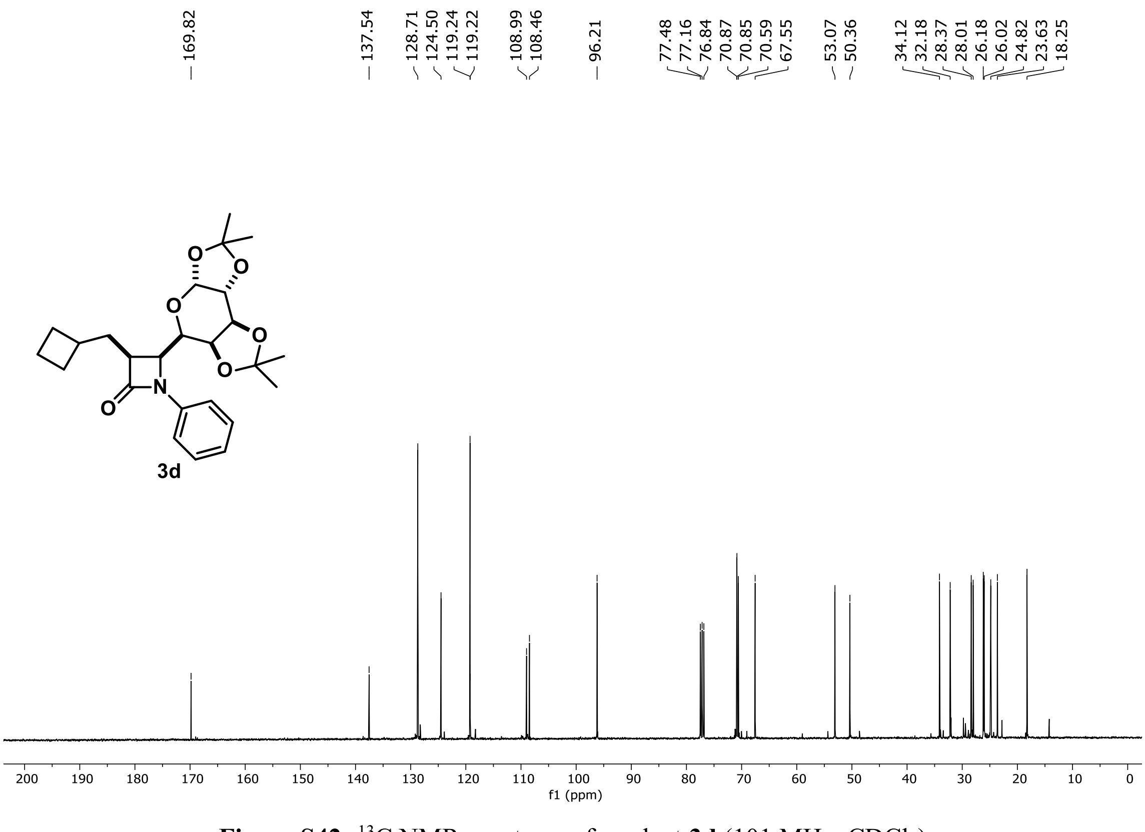

Figure S42: ^{13}C NMR spectrum of product **3d** (101 MHz, $CDCl_3$)

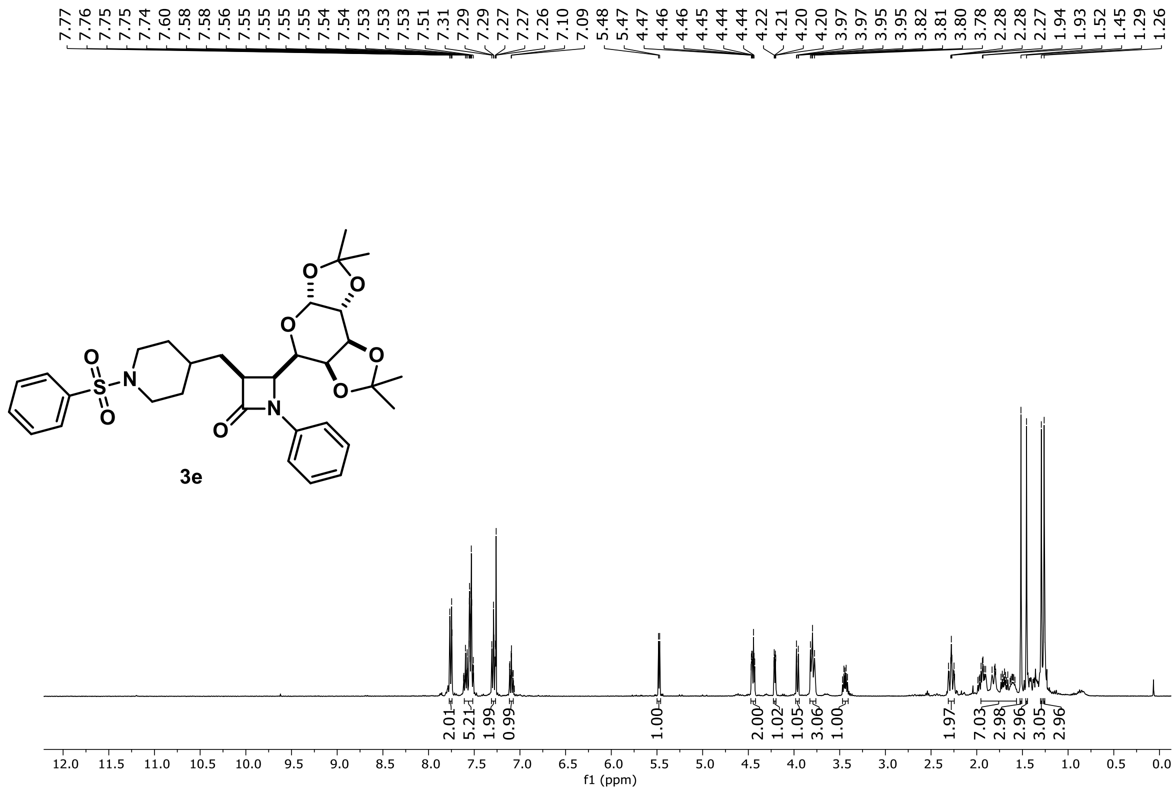

Figure S43: ^{1}H NMR spectrum of product **3e** (400 MHz, $CDCl_3$)

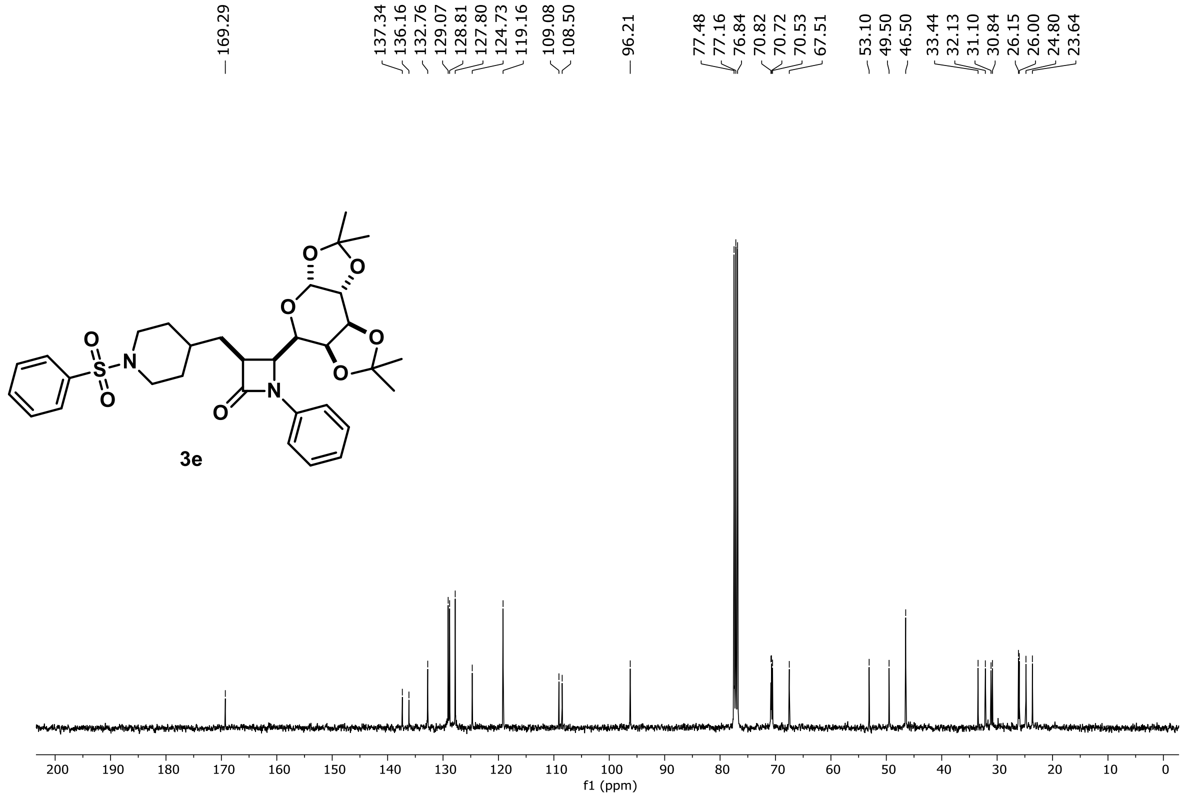

Figure S44: ^{13}C NMR spectrum of product **3e** (101 MHz, $CDCl_3$)

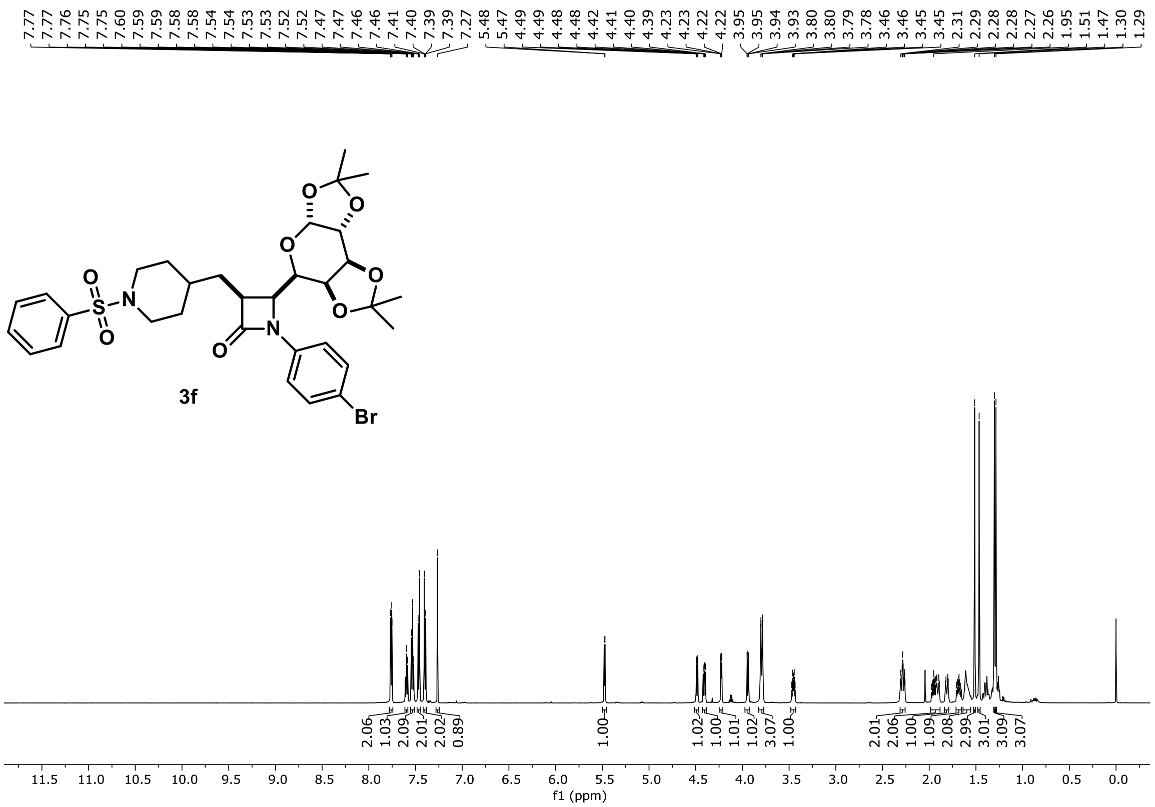

Figure S45: ^{1}H NMR spectrum of product **3f** (400 MHz, $CDCl_3$)

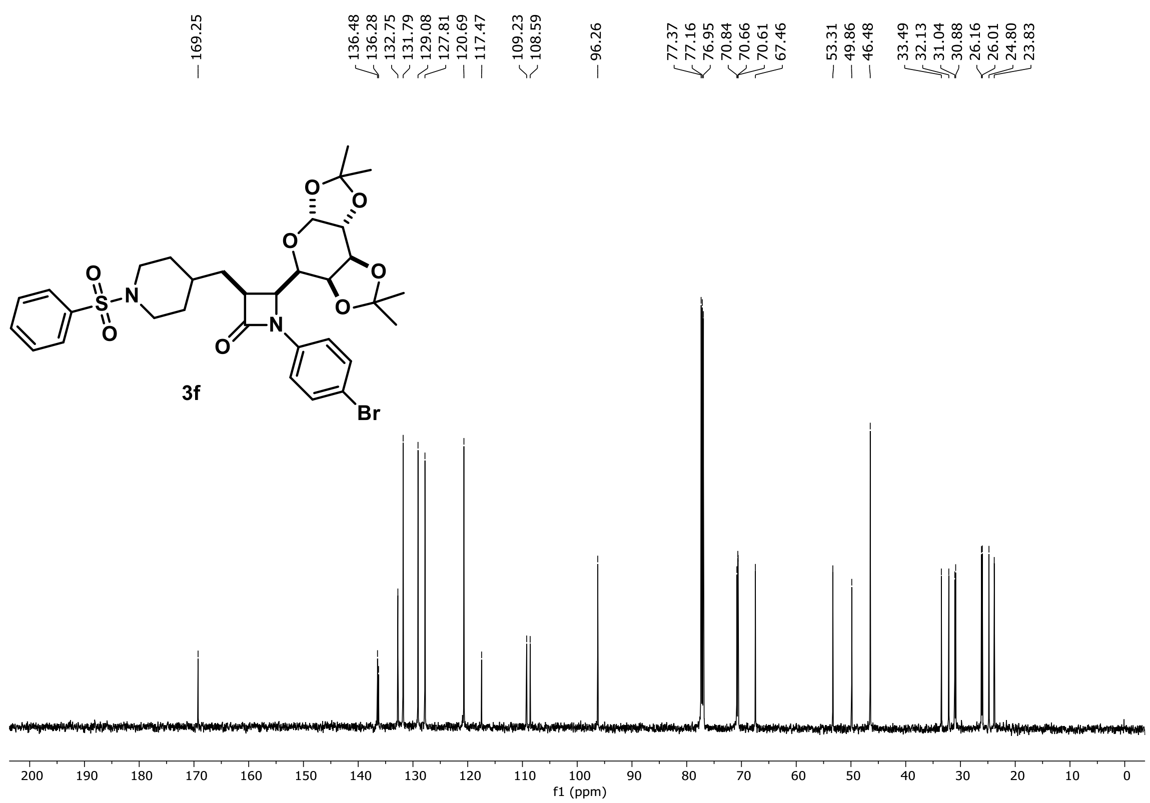

Figure S46: ^{13}C NMR spectrum of product **3f** (101 MHz, $CDCl_3$)

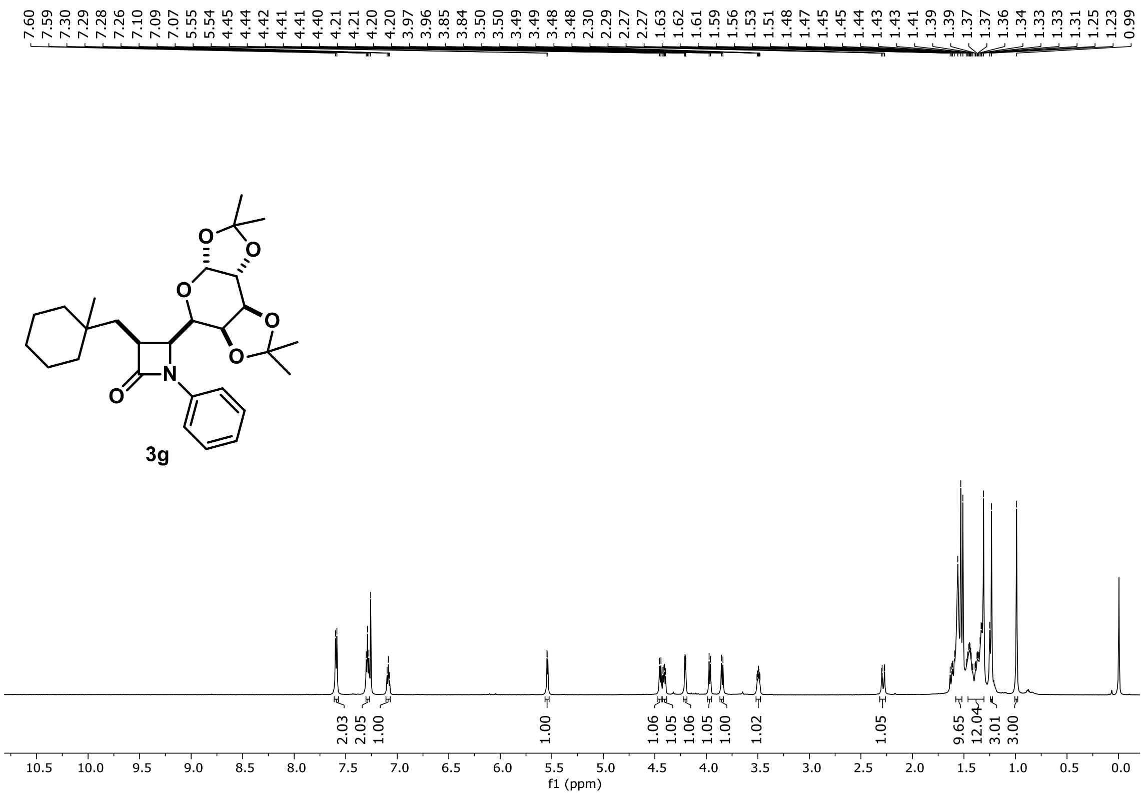

Figure S47: ^{1}H NMR spectrum of product **3g** (400 MHz, $CDCl_3$)

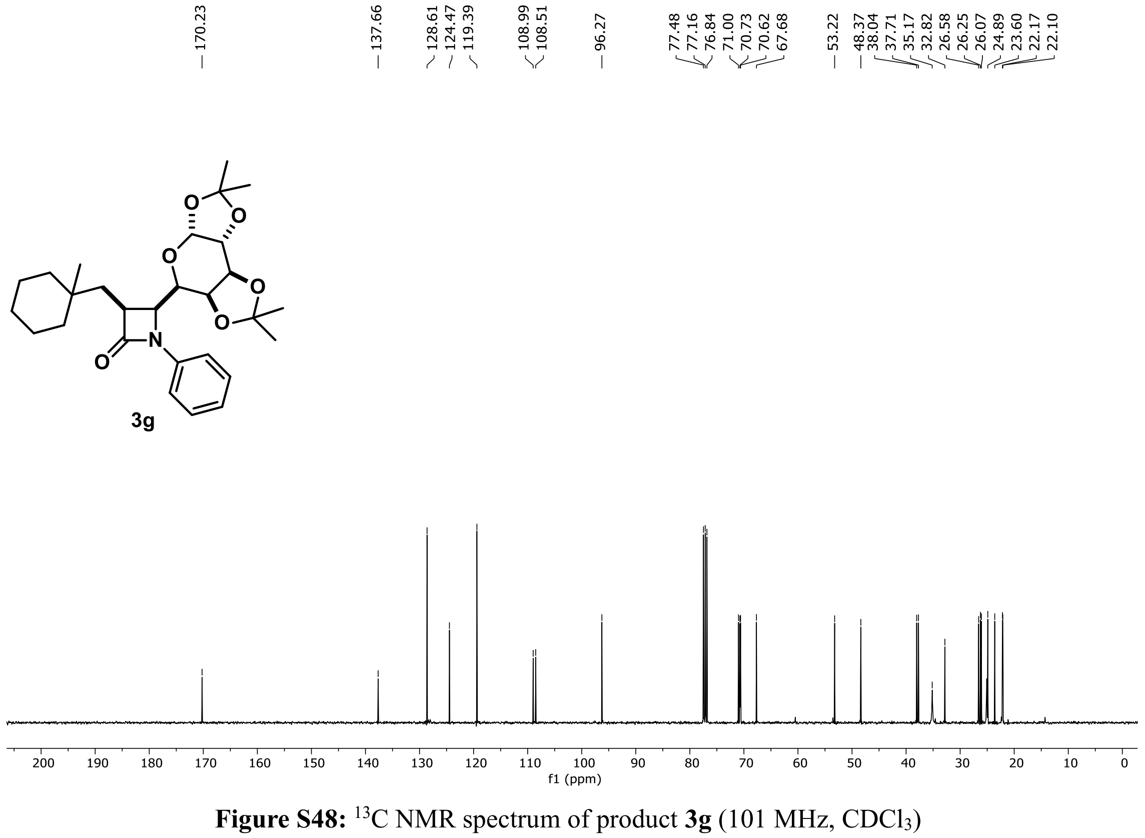

Figure S48: ^{13}C NMR spectrum of product **3g** (101 MHz, $CDCl_3$)

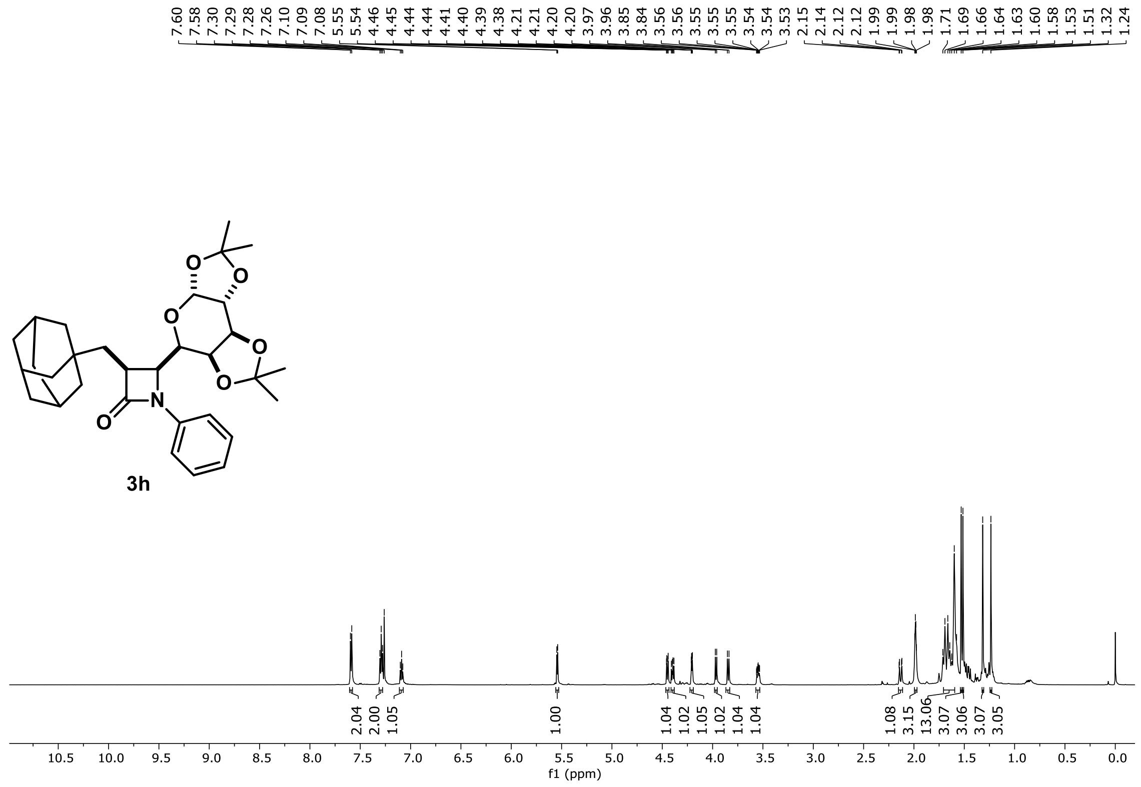

Figure S49: ^{1}H NMR spectrum of product **3h** (400 MHz, $CDCl_3$)

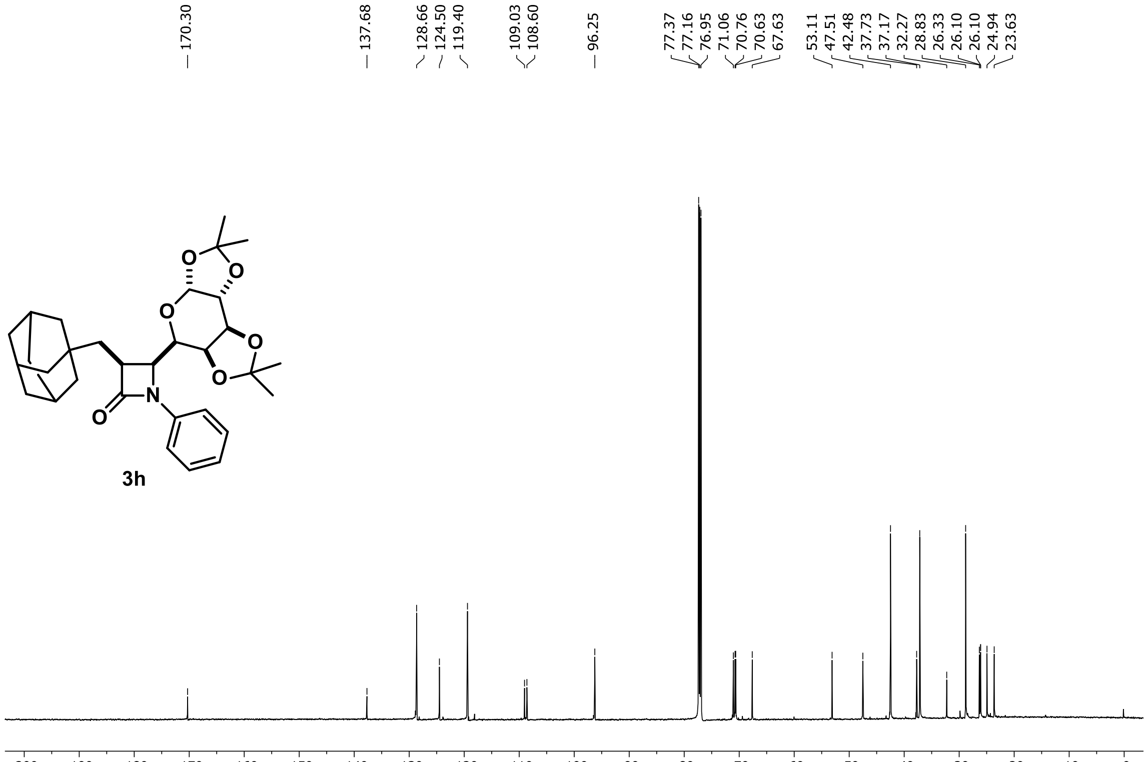

Figure S50: ^{13}C NMR spectrum of product **3h** (101 MHz, $CDCl_3$)

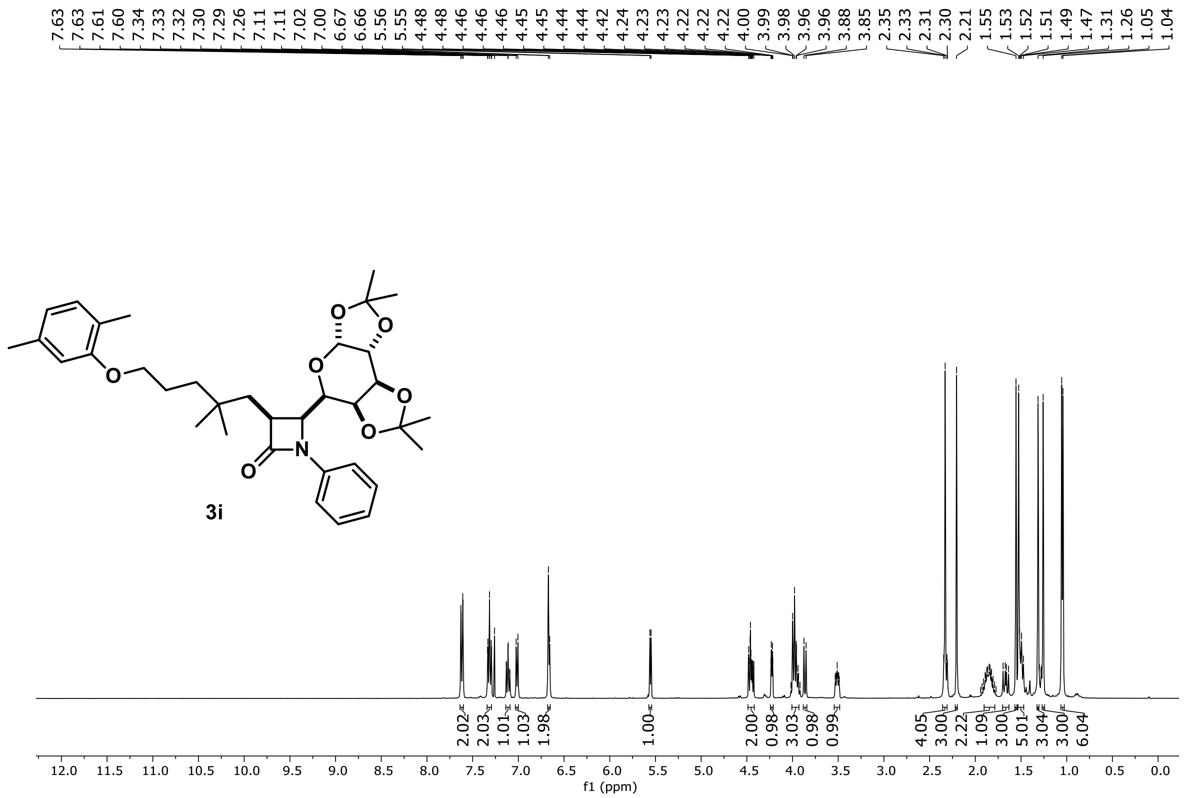

Figure S51: ^{1}H NMR spectrum of product **3i** (400 MHz, $CDCl_3$)

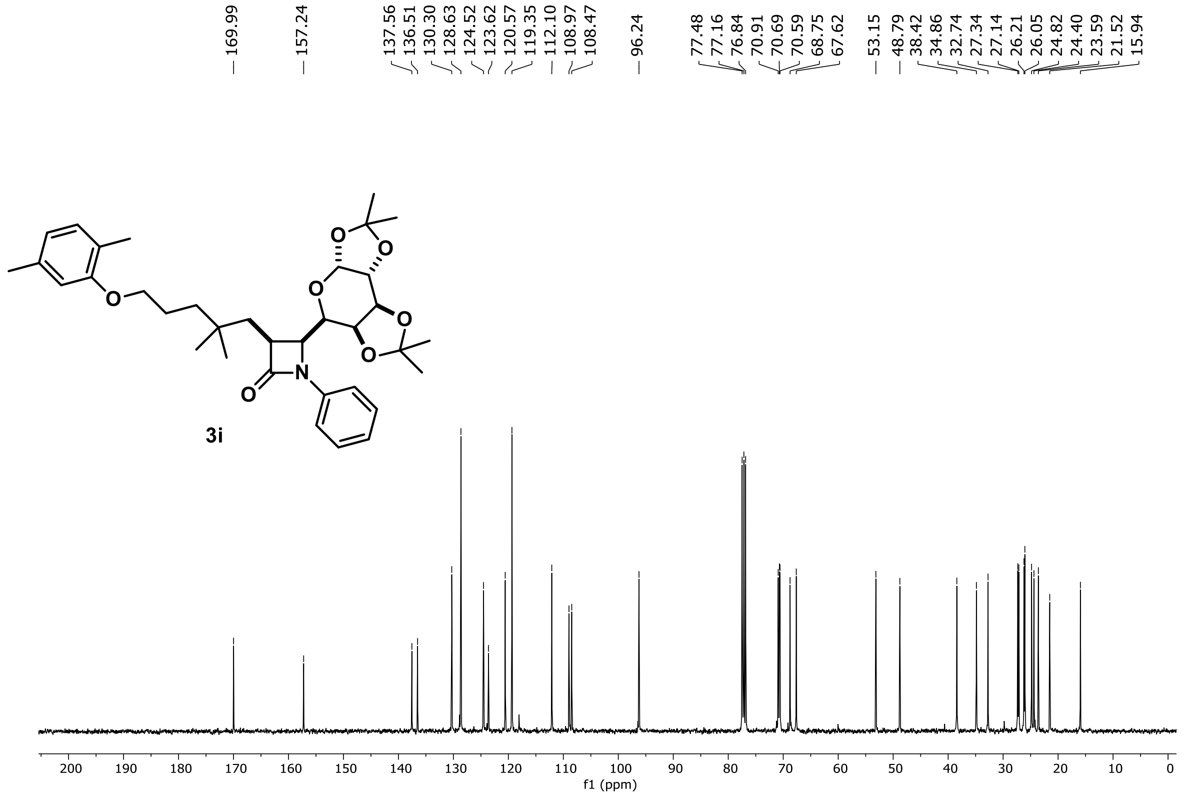

Figure S52: ^{13}C NMR spectrum of product **3i** (101 MHz, $CDCl_3$)

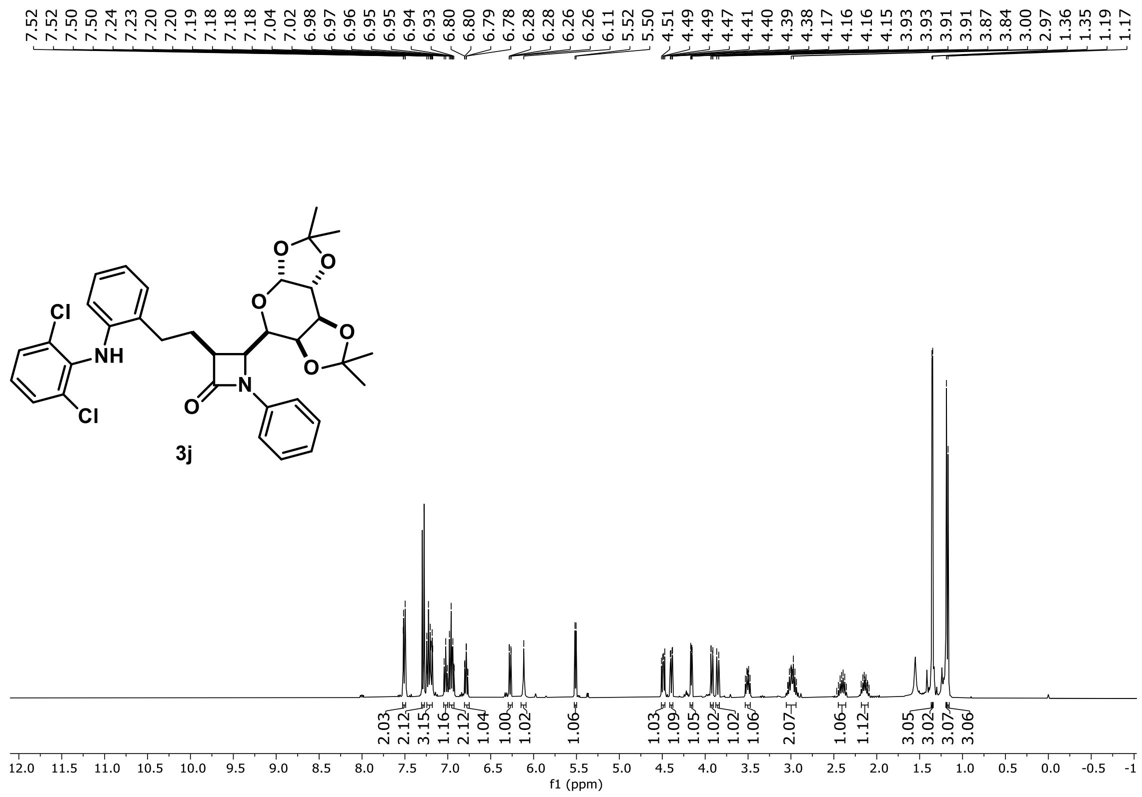

Figure S53: ^{1}H NMR spectrum of product **3j** (400 MHz, $CDCl_3$)

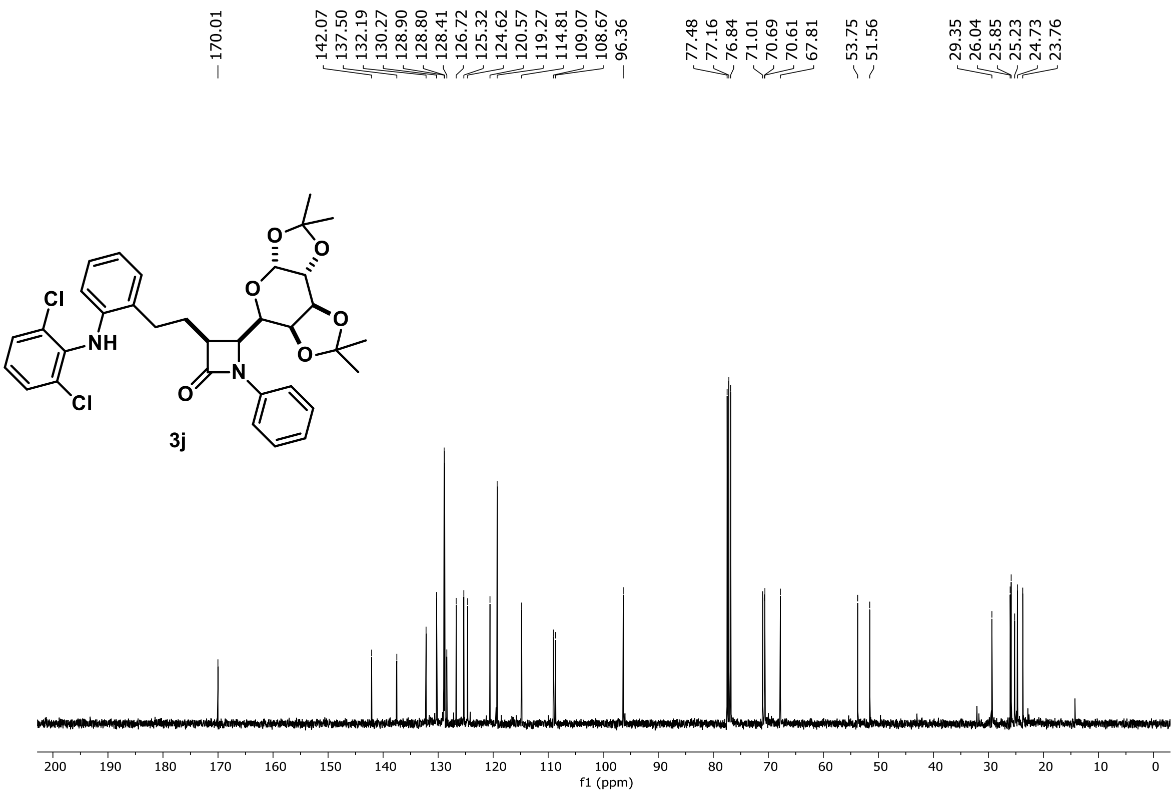

Figure S54: ^{13}C NMR spectrum of product **3j** (101 MHz, $CDCl_3$)

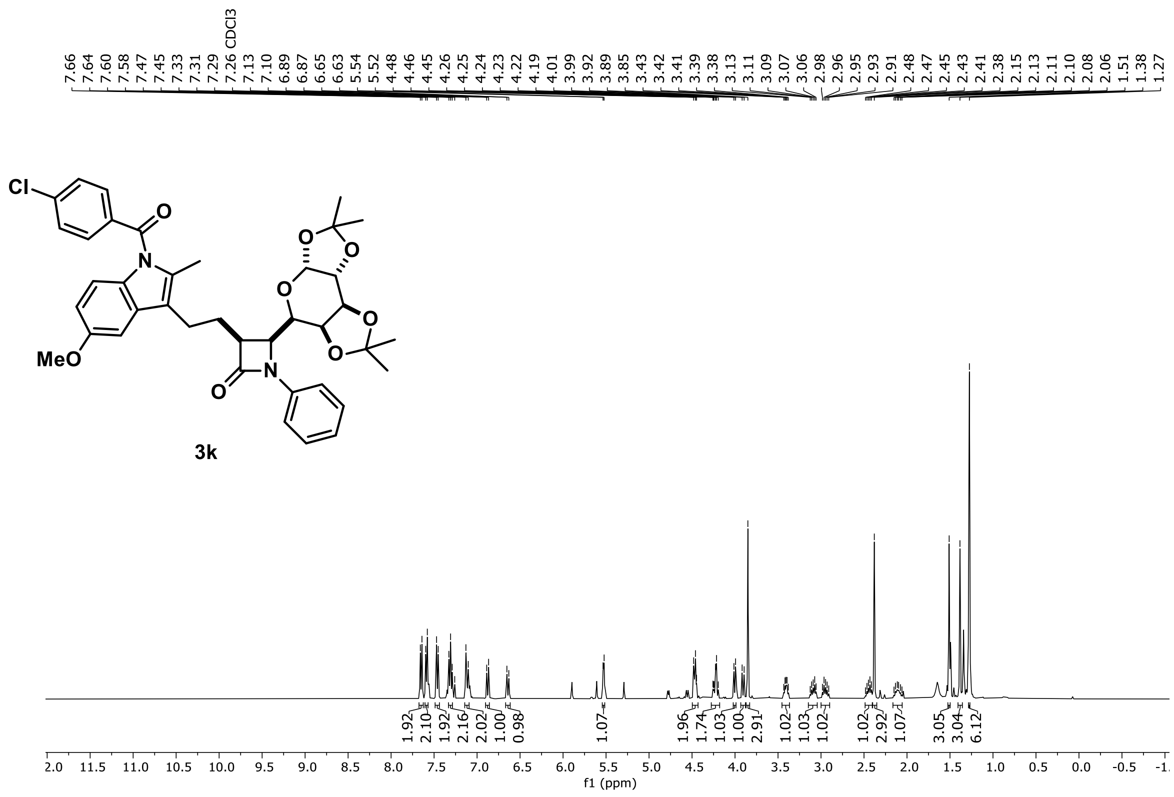

Figure S55: ^{1}H NMR spectrum of product **3k** (400 MHz, $CDCl_3$)

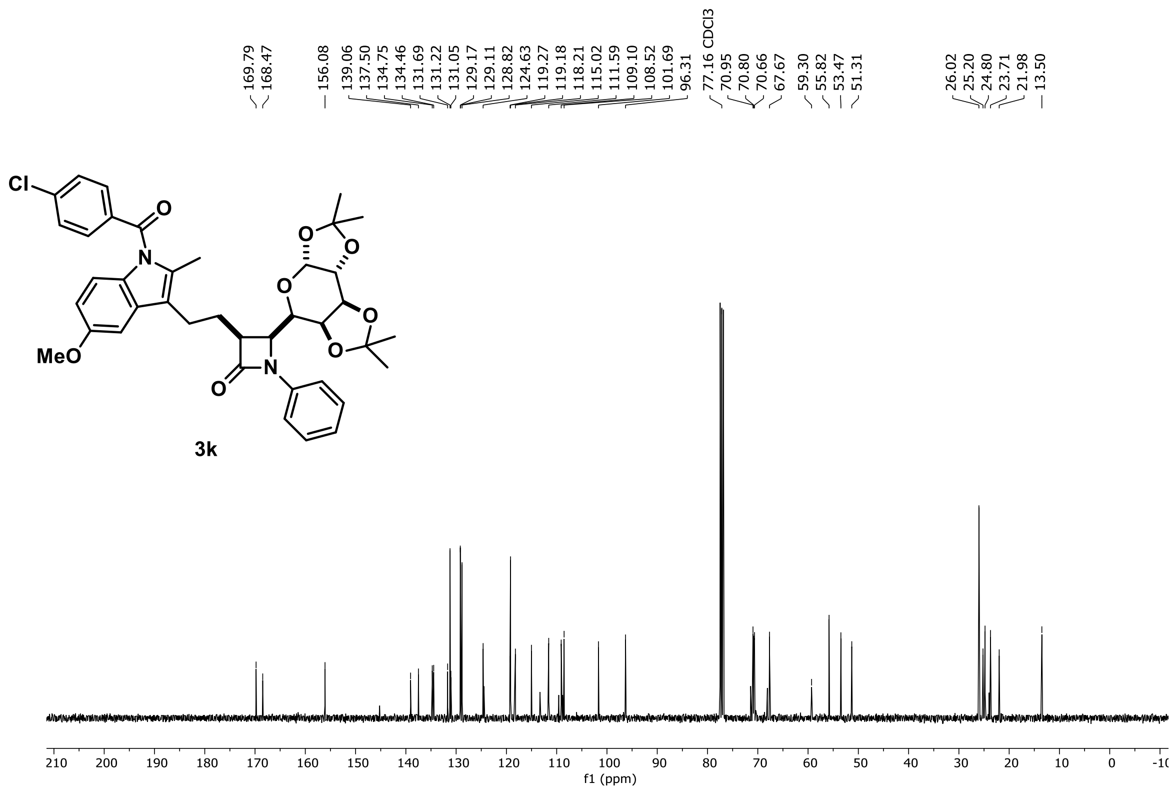

Figure S56: ^{13}C NMR spectrum of product **3k** (101 MHz, $CDCl_3$)

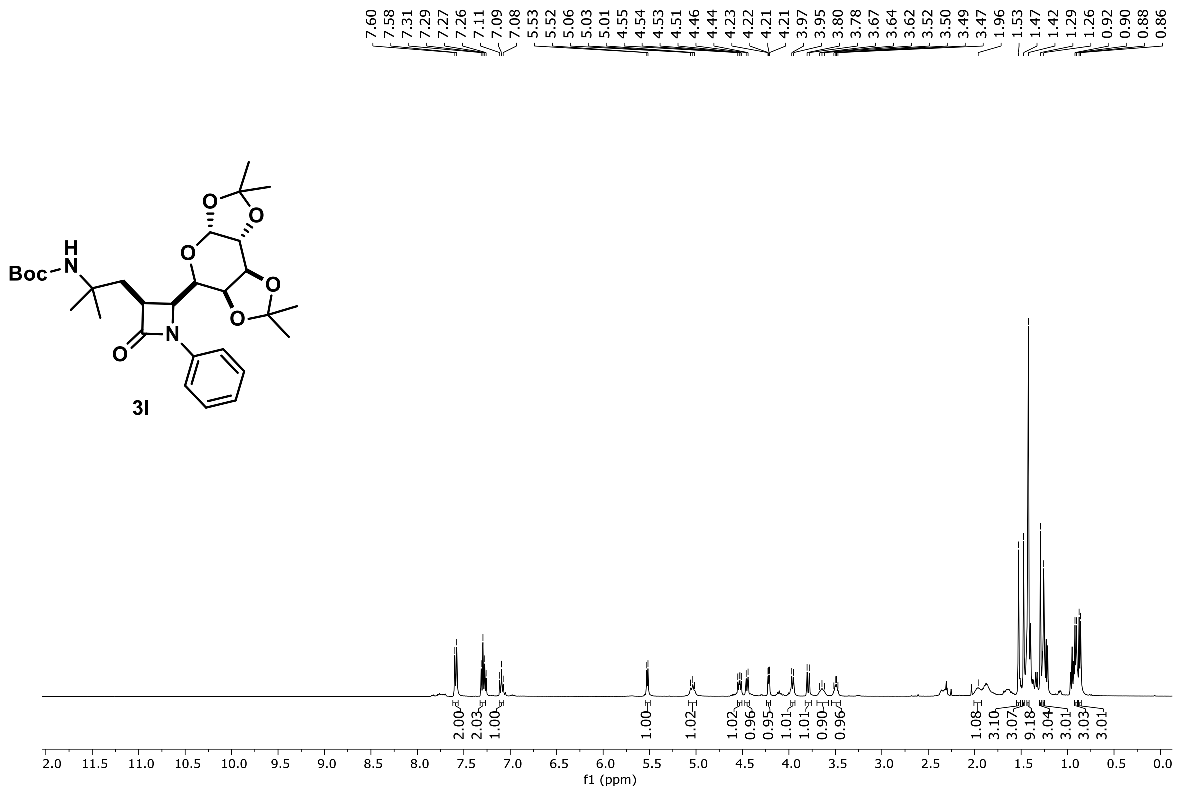

Figure S57: ^{1}H NMR spectrum of product **3l** (400 MHz, $CDCl_3$)

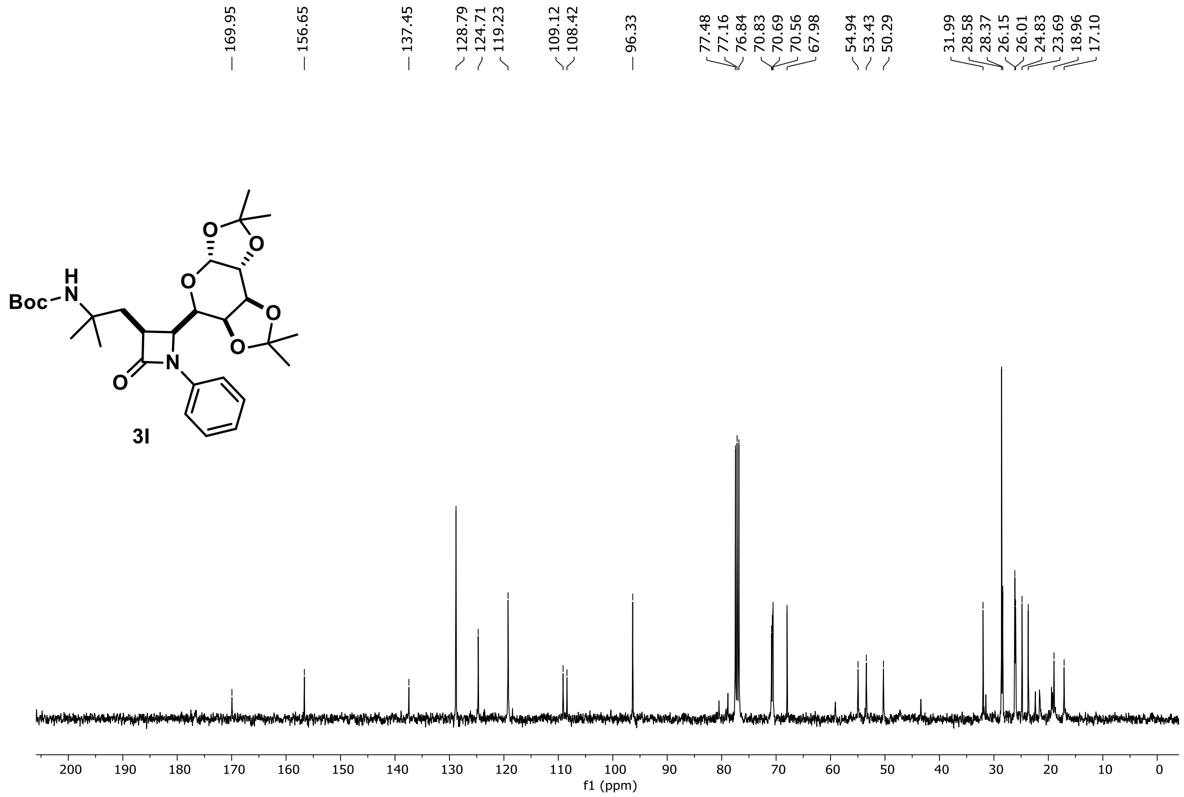

Figure S58: ^{13}C NMR spectrum of product **3l** (101 MHz, $CDCl_3$)

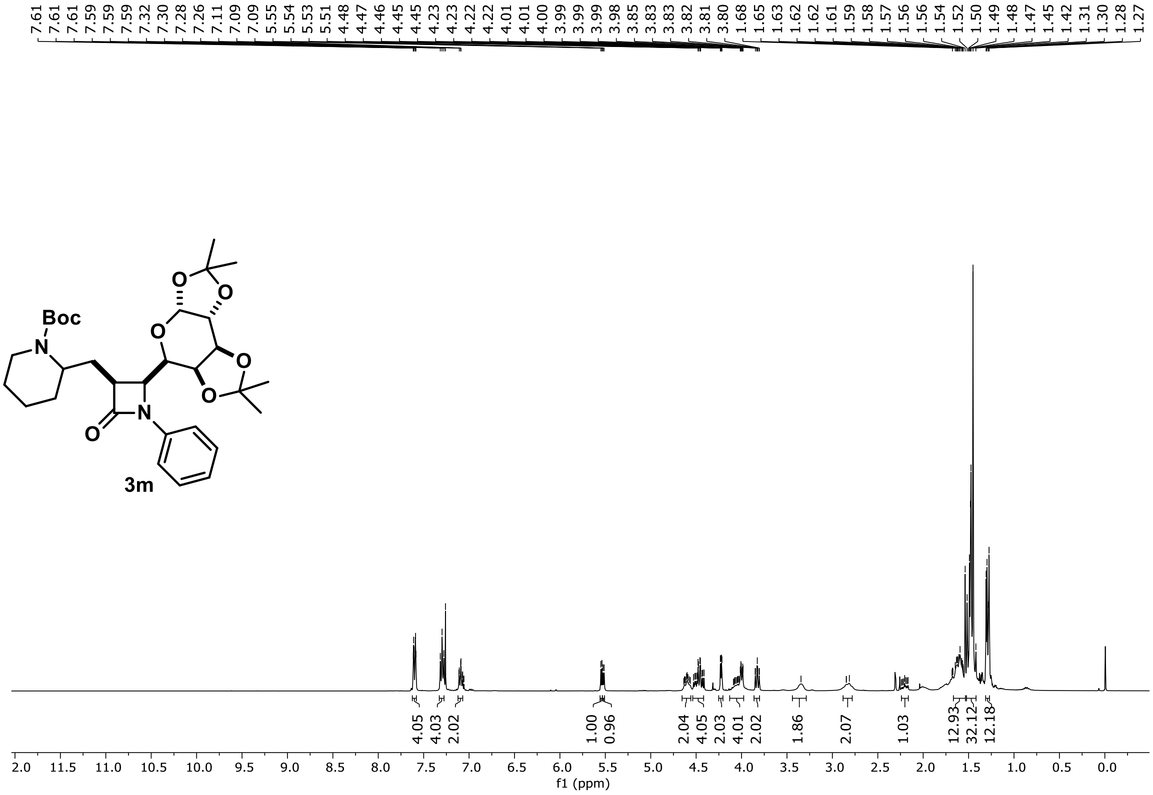

Figure S59: ^{1}H NMR spectrum of product **3m** (400 MHz, $CDCl_3$)

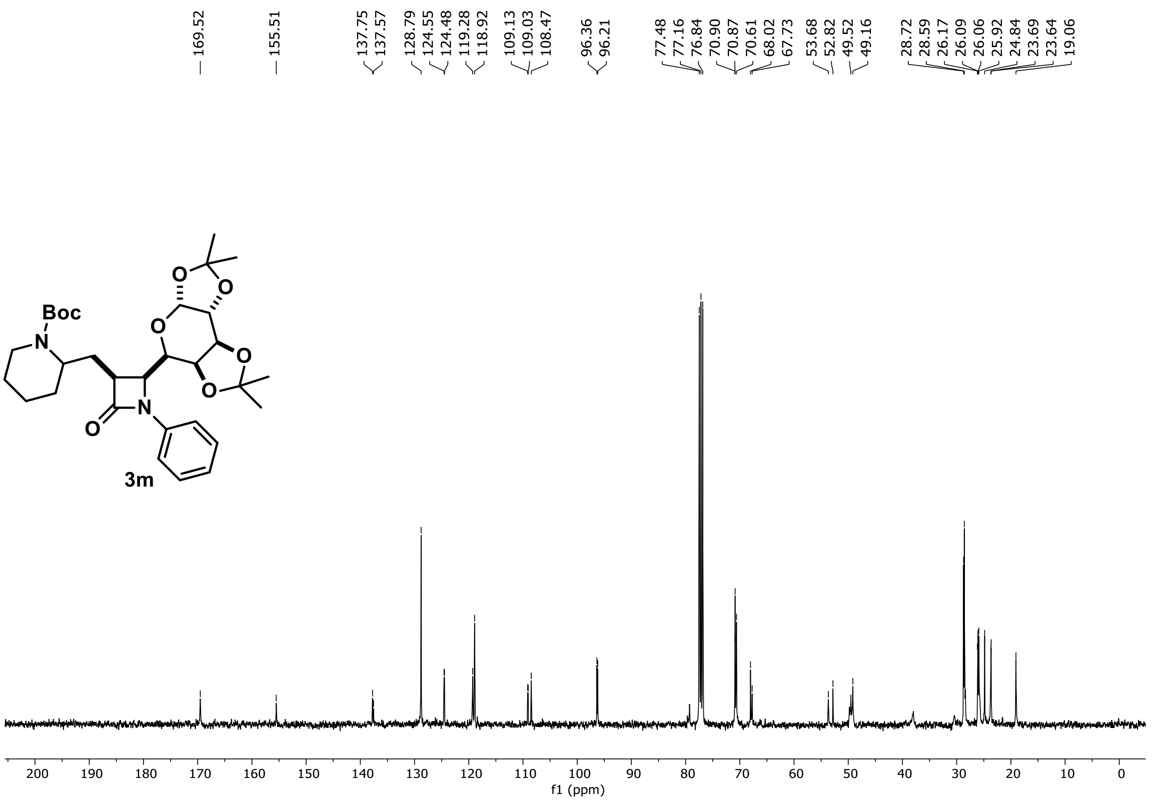

Figure S60: ^{13}C NMR spectrum of product **3m** (101 MHz, $CDCl_3$)

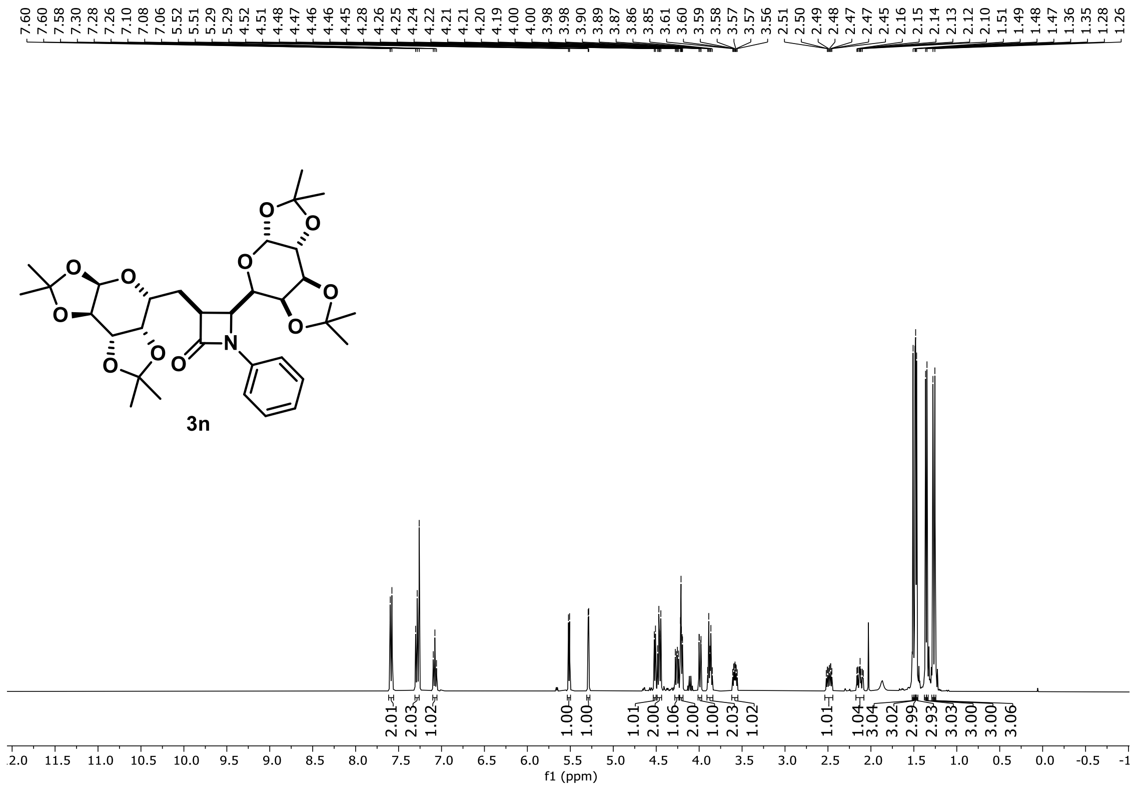

Figure S61: ^{1}H NMR spectrum of product **3n** (400 MHz, $CDCl_3$)

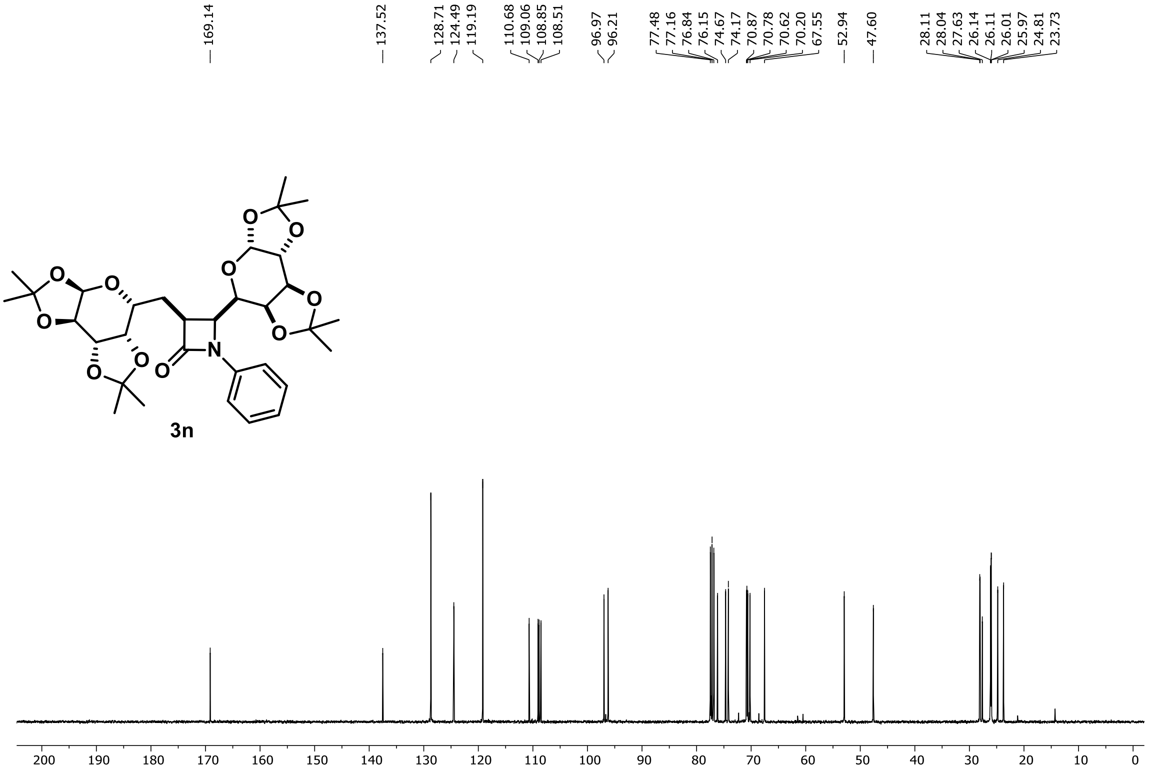

Figure S62: ^{13}C NMR spectrum of product **3n** (101 MHz, $CDCl_3$)

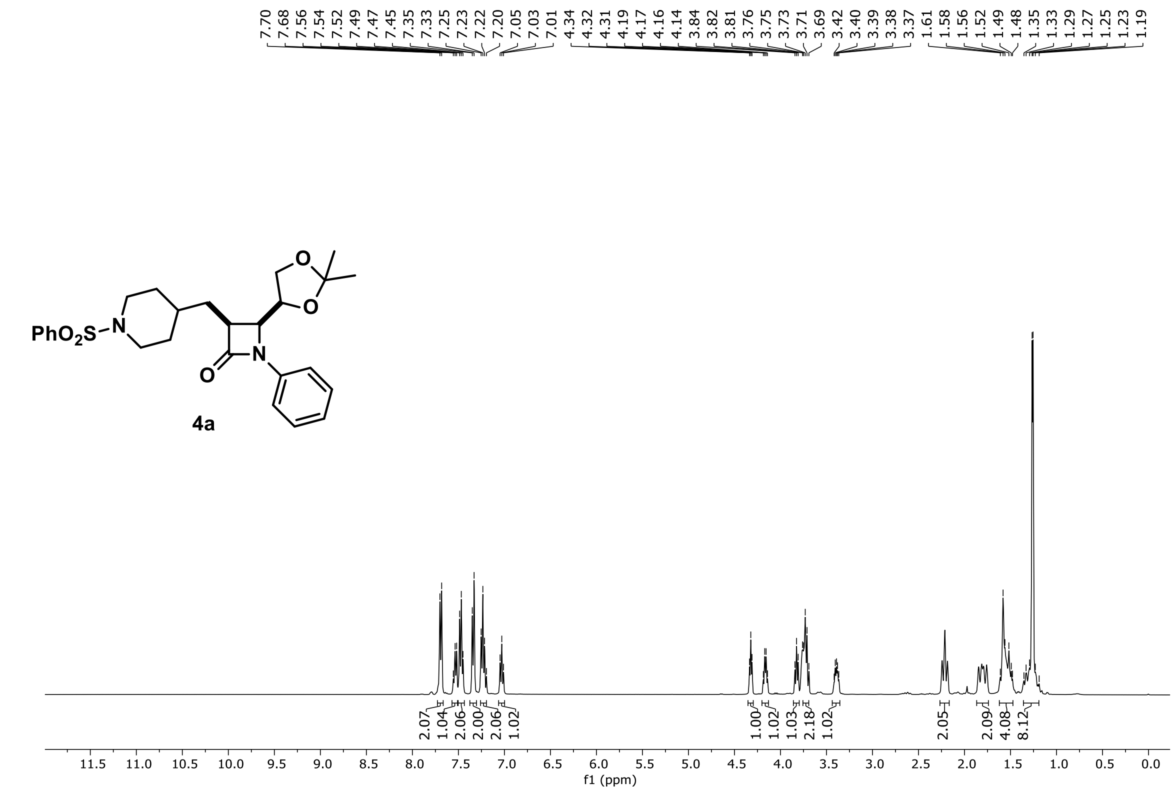

Figure S63: ^{1}H NMR spectrum of product **4a** (400 MHz, $CDCl_3$)

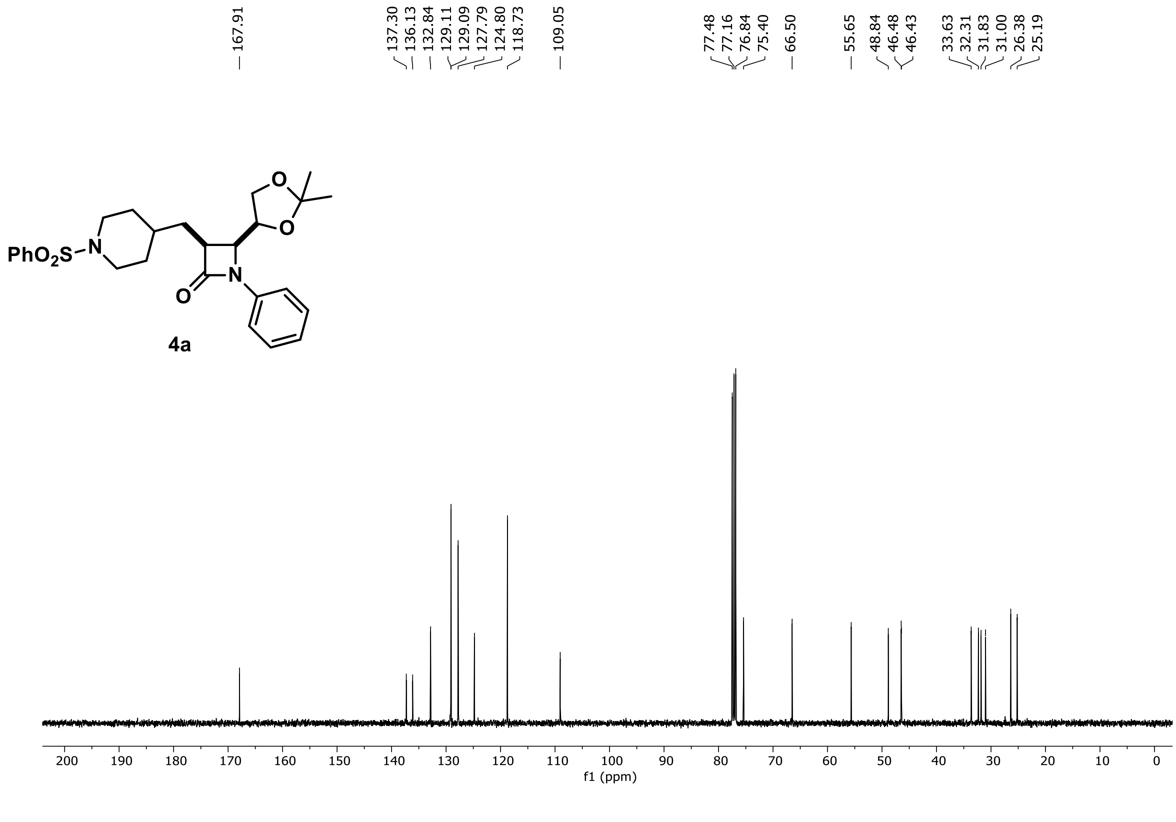

Figure S64: ^{13}C NMR spectrum of product **4a** (101 MHz, $CDCl_3$)

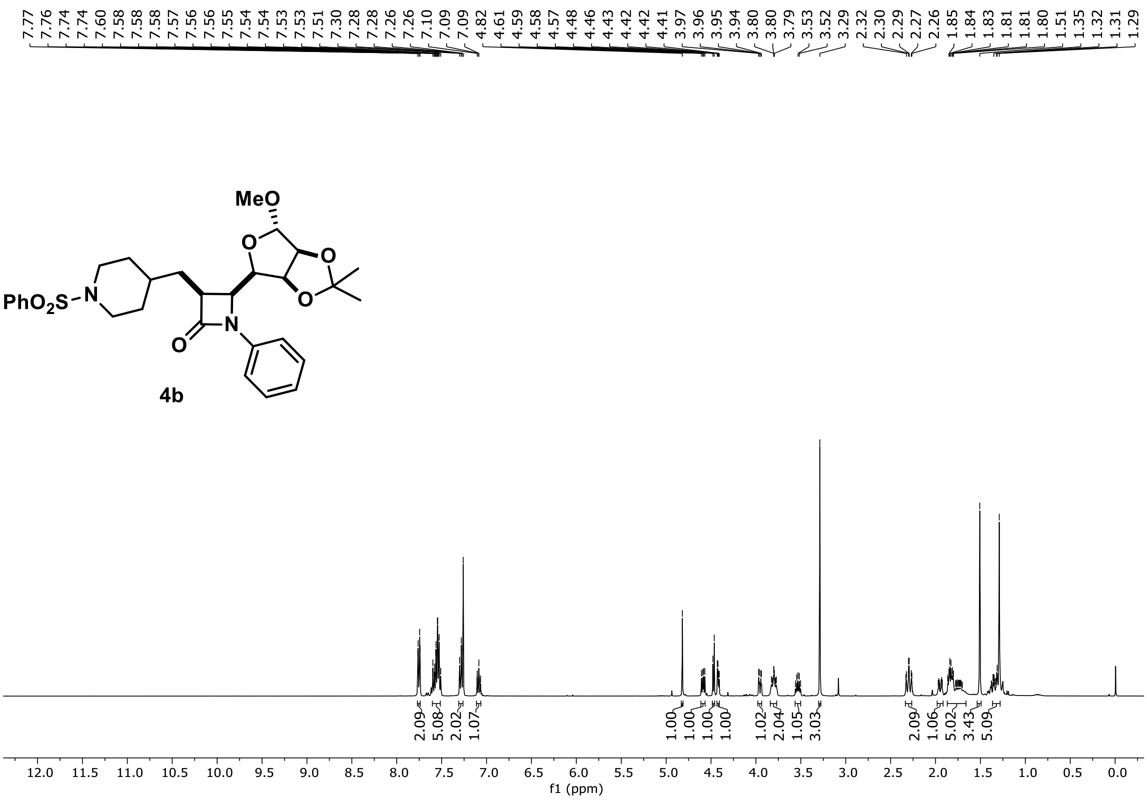

Figure S65: ^{1}H NMR spectrum of product **4b** (400 MHz, $CDCl_3$)

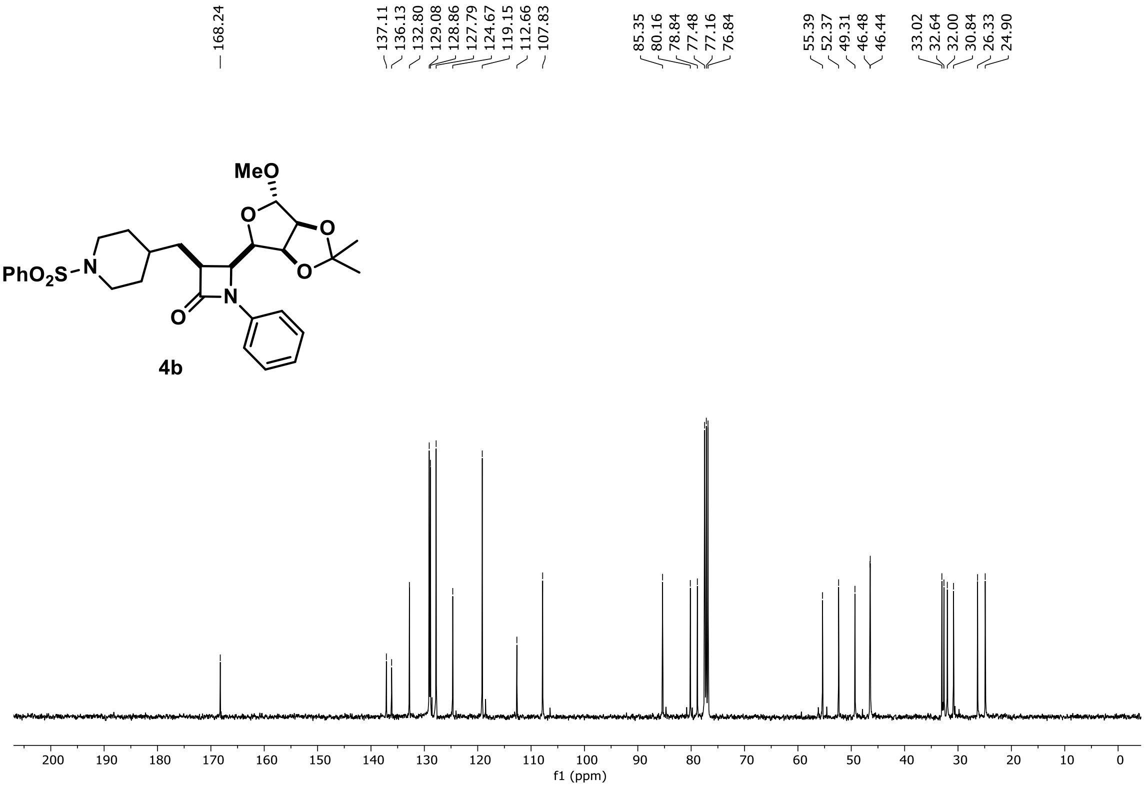

Figure S66: ^{13}C NMR spectrum of product **4b** (101 MHz, $CDCl_3$)

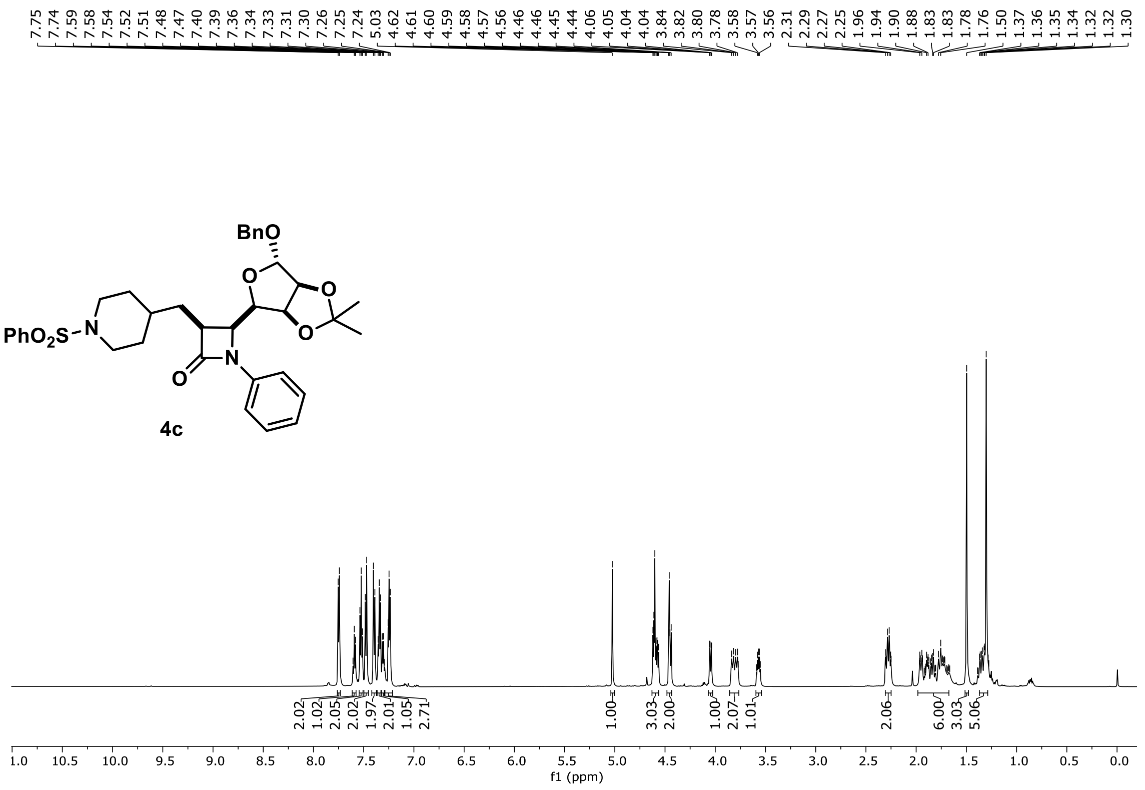

Figure S67: ^{1}H NMR spectrum of product **4c** (400 MHz, $CDCl_3$)

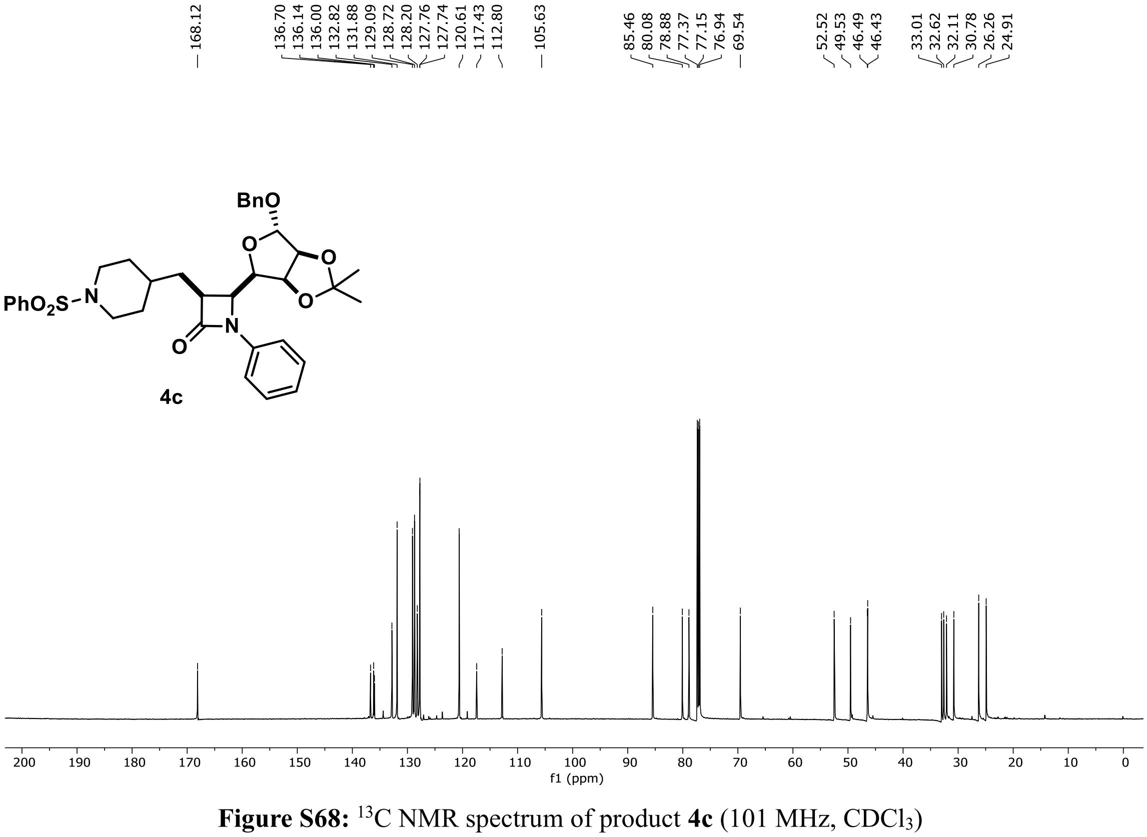

Figure S68: ^{13}C NMR spectrum of product **4c** (101 MHz, $CDCl_3$)

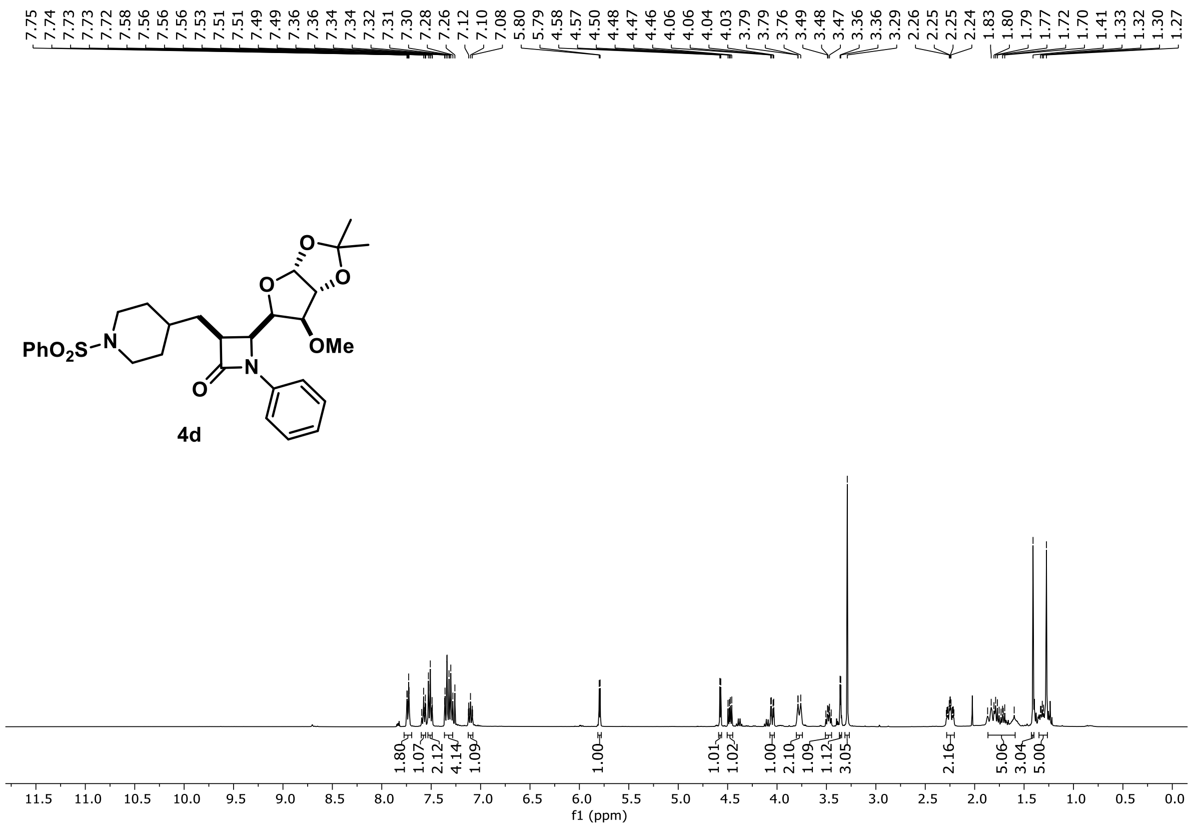

Figure S69: ^{1}H NMR spectrum of product **4d** (400 MHz, $CDCl_3$)

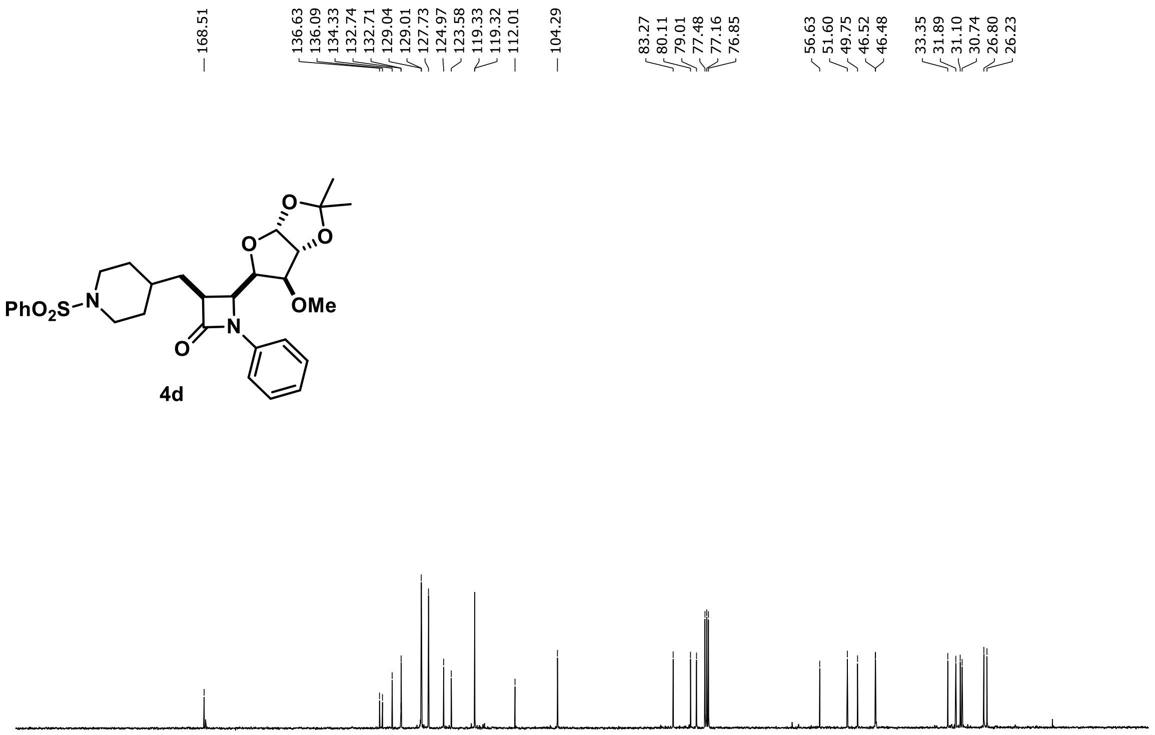

Figure S70: ^{13}C NMR spectrum of product **4d** (101 MHz, $CDCl_3$)

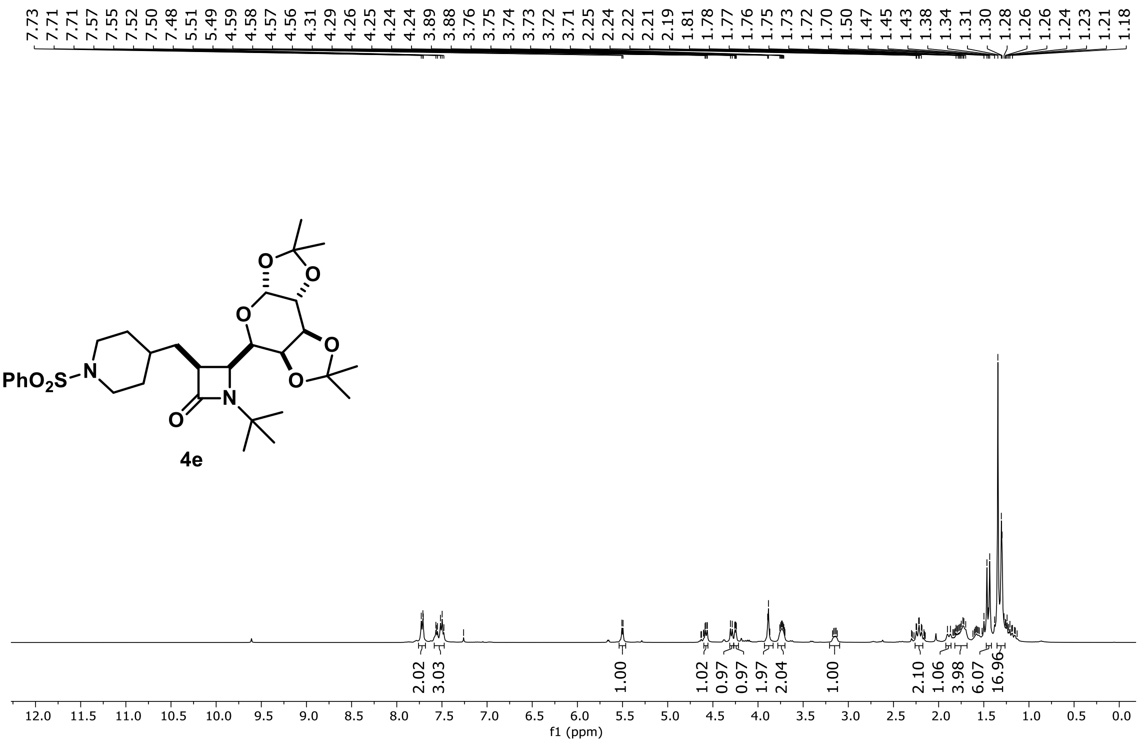

Figure S71: ^{1}H NMR spectrum of product **4e** (400 MHz, $CDCl_3$)

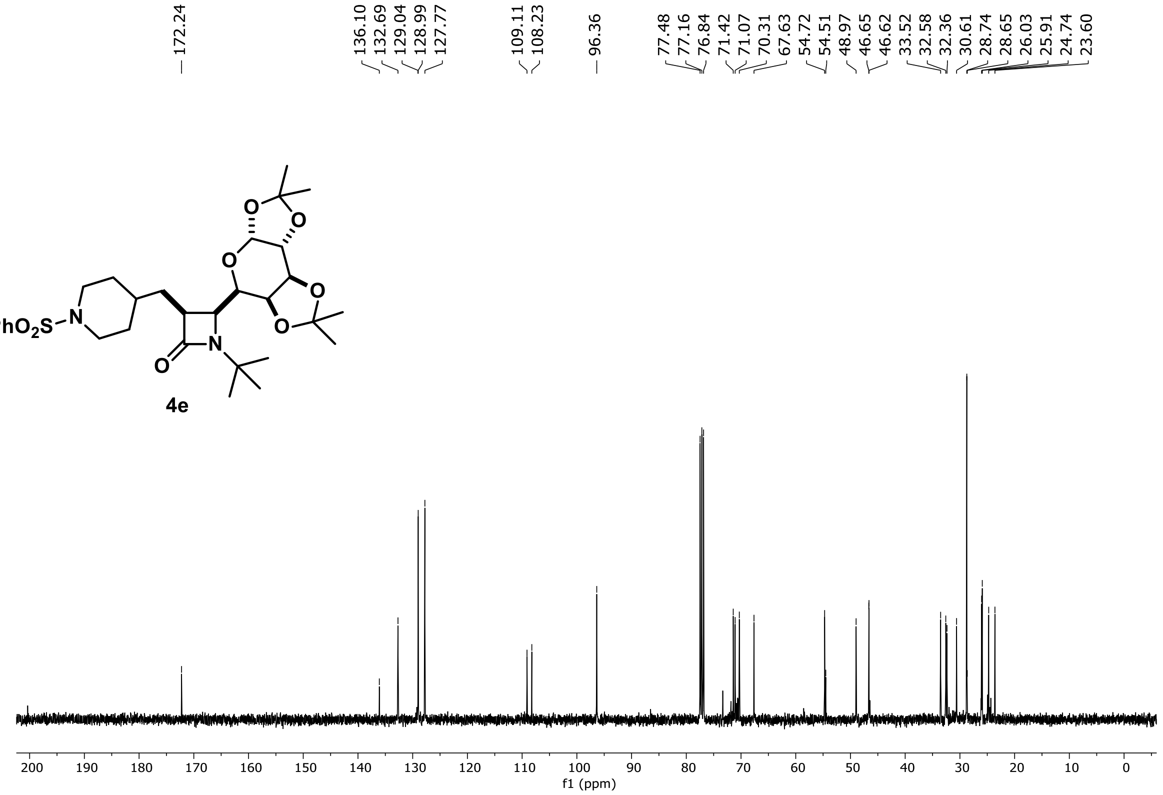

Figure S72: ^{13}C NMR spectrum of product **4e** (101 MHz, $CDCl_3$)

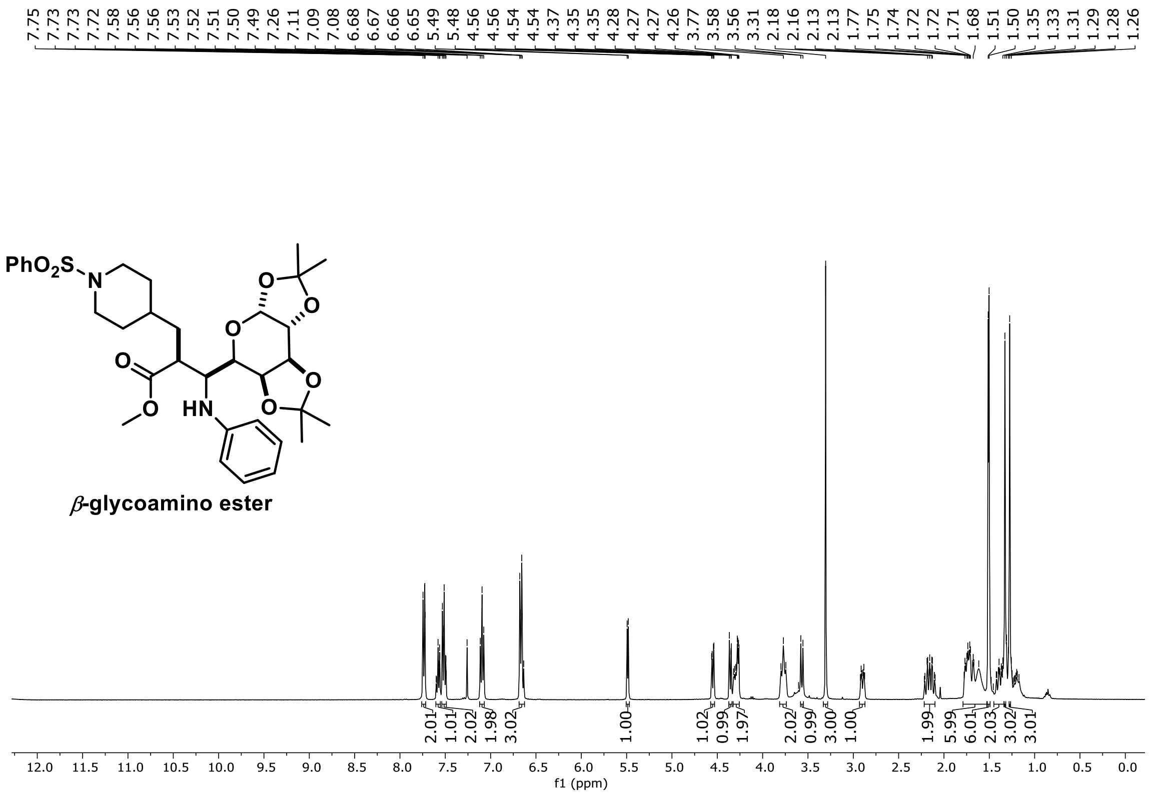

Figure S73: ^{1}H NMR spectrum of ***β*-glycoamino ester** (400 MHz, $CDCl_3$)

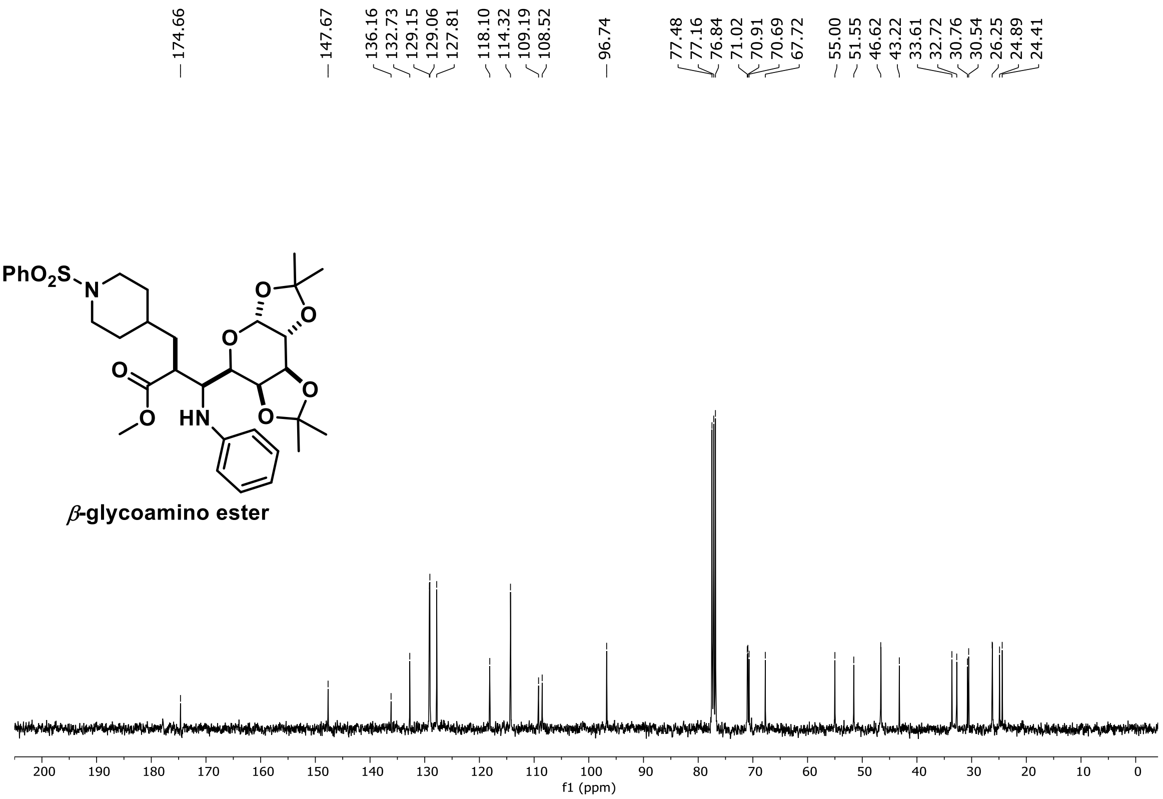

Figure S74: ^{13}C NMR spectrum of ***β*-glycoamino ester** (101 MHz, $CDCl_3$)

7. References

(1) Armarego, W. L. F. *Purification of Laboratory Chemicals*, Eighth Edition.; 2017.

(2) Schneider, L. M.; Schmiedel, V. M.; Pecchioli, T.; Lentz, D.; Merten, C.; Christmann, M. Asymmetric Synthesis of Carbocyclic Propellanes. *Org. Lett.* **2017**, *19* (9), 2310–2313.

(3) Neises, B.; Steglich, W. Simple Method for the Esterification of Carboxylic Acids. *Angew. Chem. Int. Ed.* **1978**, *17* (7), 522–524.

(4) Jia, Y.; Dong, X.; Zhou, P.; Liu, X.; Pan, L.; Xin, H.; Zhun Zhu, Y.; Wang, Y. The Synthesis and Biological Evaluation of Novel Danshensuecysteine Analog Conjugates as Cardiovascular-Protective Agents. *Eur J Med Chem* **2012**, *55*, 176–187.

(5) Shao, Y. M.; Yang, W. Bin; Peng, H. P.; Hsu, M. F.; Tsai, K. C.; Kuo, T. H.; Wang, A. H. J.; Liang, P. H.; Lin, C. H.; Yang, A. S.; Wong, C. H. Structure-Based Design and Synthesis of Highly Potent SARS-CoV 3CL Protease Inhibitors. *ChemBioChem* **2007**, *8* (14), 1654–1657.

(6) Banks, M. R.; Cadogan, J. I. G.; Gosney, I.; Gould, R. O.; Hodgson, P. K. G.; Mcdougall, D. Preparation of Enantiomerically Pure Fructose-Derived 1,3-Oxazin-2-One by INIR Methodology and Its Application as a Chiral Auxiliary in Some Model Asymmetric Reactions. *Pergamon Tetrahedron* **1998**, *54*, 9765–9784.

(7) De Luca, L.; Giacomelli, G.; Masala, S.; Porcheddu, A. Trichloroisocyanuric/TEMPO Oxidation of Alcohols under Mild Conditions: A Close Investigation. *J. Org. Chem.* **2003**, *68* (12), 4999–5001.

(8) Tortajada, A.; Duan, Y.; Sahoo, B.; Cong, F.; Toupalas, G.; Sallustrau, A.; Loreau, O.; Audisio, D.; Martin, R. Catalytic Decarboxylation/Carboxylation Platform for Accessing Isotopically Labeled Carboxylic Acids. *ACS Catal* **2019**, *9* (7), 5897–5901.

(9) Toriyama, F.; Cornella, J.; Wimmer, L.; Chen, T. G.; Dixon, D. D.; Creech, G.; Baran, P. S. Redox-Active Esters in Fe-Catalyzed C-C Coupling. *J. Am. Chem. Soc.* **2016**, *138* (35), 11132–11135.

(10) Cornella, J.; Edwards, J. T.; Qin, T.; Kawamura, S.; Wang, J.; Pan, C. M.; Gianatassio, R.; Schmidt, M.; Eastgate, M. D.; Baran, P. S. Practical Ni-Catalyzed Aryl-Alkyl Cross-Coupling of Secondary Redox-Active Esters. *J. Am. Chem. Soc.* **2016**, *138* (7), 2174–2177.

(11) Pratsch, G.; Lackner, G. L.; Overman, L. E. Constructing Quaternary Carbons from N - (Acyloxy)Phthalimide Precursors of Tertiary Radicals Using Visible-Light Photocatalysis. *J. Org. Chem.* **2015**, *80* (12), 6025–6036.

(12) Wang, D.; Zhu, N.; Chen, P.; Lin, Z.; Liu, G. Enantioselective Decarboxylative Cyanation Employing Cooperative Photoredox Catalysis and Copper Catalysis. *J. Am. Chem. Soc.* **2017**, *139* (44), 15632–15635.

(13) Zhang, Z.; Cernak, T. The Formal Cross-Coupling of Amines and Carboxylic Acids to Form Sp3–Sp3 Carbon–Carbon Bonds. *Angew. Chem. Int. Ed.* **2021**, *60* (52), 27293–27298.

(14) Correia, J. T. M.; Piva Da Silva, G.; Kisukuri, C. M.; André, E.; Pires, B.; Carneiro, P. S.; Paixão, M. W. Metal-Free Photoinduced Hydroalkylation Cascade Enabled by an Electron-Donor-Acceptor Complex. *J. Org. Chem.* **2020**, *85* (15), 9820–9834.

(15) Zhang, Y. L.; Yang, L.; Wu, J.; Zhu, C.; Wang, P. Vinyl Sulfonium Salts as the Radical Acceptor for Metal-Free Decarboxylative Alkenylation. *Org. Lett.* **2020**, *22* (19), 7768–7772.

(16) Mills, L. R.; Zhou, C.; Fung, E.; Rousseaux, S. A. L. Ni-Catalyzed β-Alkylation of Cyclopropanol-Derived Homoenolates. *Org. Lett.* **2019**, *21* (21), 8805–8809.

(17) Qi, R.; Wang, C.; Ma, Z.; Wang, H.; Chen, Q.; Liu, L.; Pan, D.; Ren, X.; Wang, R.; Xu, Z. Visible-Light-Promoted Stereoselective $C(sp^3)-H$ Glycosylation for the Synthesis of C-Glycoamino Acids and C-Glycopeptides. *Angew. Chem. Int. Ed.* **2022**, *61* (24).

(18) Patrick, T. B.; Schield, J. A.; Kirchner, D. G. Synthesis of Fluoroaromatic Amines. *J. Org. Chem.* **1974**, *39* (12), 1758–1761.

(19) Ram, R. N.; Soni, V. K. Synthesis of 3-Alkylbenzoxazolones from *N*-Alkyl-*N*-Arylhydroxylamines by Contiguous O-Trichloroacetylation, Trichloroacetoxy Ortho-Shift, and Cyclization Sequence. *J. Org. Chem.* **2013**, *78* (23), 11935–11947.

(20) Ayyangar, N. R.; Brahme, K. C.; Kalkote, U. R.; Srinivasan, K. V. Facile Transfer-Reduction of Nitronarenes to *N*-Arylhydroxylamines with Hydrazine in the Presence of Raney Nickel. *Synthesis (Stuttg)* **1984**, *1984* (11), 938–941.

(21) Hojczyk, K. N.; Feng, P.; Zhan, C.; Ngai, M. Y. Trifluoromethoxylation of Arenes: Synthesis of Ortho-Trifluoromethoxylated Aniline Derivatives by OCF3 Migration. *Angewandte Chemie International Edition* **2014**, *53* (52), 14559–14563.

(22) a) Rondestvedt, C. S.; Jr; Johnson, T. A. Explosion of an Arylhydroxylamine During Preparation of 2-Chloro-5-Methylaniline. Failure of the Palladium-Catalyzed Hydrazine Reduction. *Synthesis (Stuttg)* **1977**, 851–852. b) Gad, S. E.; Hydroxylamine. Encyclopedia of Toxicology (Second Edition), **2005**, 557-558.

(23) Stowell, J. C. Tert-Alkylnitroso Compounds. Synthesis and Dimerization Equilibria. *J. Org. Chem.* **1971**, *36* (20), 3055–3056.

(24) Carney, J. M. 2,4-Disulfo Phenyl Butyl Nitrone, Its Salts and Their Use as Pharmaceuticals, 1998.

(25) Greene, F. D.; Pazos, J. F. Diaziridinones. IV. Formation by Condensation of Alkyl Isocyanide with Nitrosoalkane. Evidence for a Carbodiimide *N*-Oxide. *J. Org. Chem.* **1969**, *34* (8), 2269–2274.

(26) Natarajan, P.; Chaudhary, R.; Venugopalan, P. Silver(I)-Promoted Ipso-Nitration of Carboxylic Acids by Nitronium Tetrafluoroborate. *Journal of Organic Chemistry* **2015**, *80* (21), 10498–10504.

(27) de Alvarenga, E. S.; Carneiro, V. M. T.; Silvério, F. O.; Saliba, W. A. A High Yield Synthesis of 1,2:5,6-Di-O-Isopropylidene-D-Mannitol. *J. Chil. Chem. Soc* **2006**, *51* (3), 986–988.

(28) a) Schmidt, A. K. C.; Stark, C. B. W. The Glycol Cleavage in Natural Product Synthesis: Reagent Classics and Recent Advances. *Synthesis (Stuttg)* **2014**, *46* (24), 3283–3308. b) Zhong, Y.-L.; Shing, T. K. M. Efficient and Facile Glycol Cleavage Oxidation Using Improved Silica Gel-Supported Sodium Metaperiodate. *J. Org. Chem.* **1997**, *62* (8), 2622–2624. c) Perlin, A. S. Glycol-Cleavage Oxidation. *Advances in Carbohydrate Chemistry and Biochemistry*. Academic Press Inc. 2006, pp 183–250. d) Bobbitt, J. M. Periodate oxidation of carbohydrates. *Adv Carbohydr Chem* **1956**, *11*, 1–41.

(29) Gautam, D.; Kumar, D. N.; Rao, B. V. Chiron Approach for the Synthesis of (1S,2R,5R,7S)-2-Hydroxy-Exo-Brevicomin. *Tetrahedron Asymmetry* **2006**, *17* (5), 819–821.

(30) a) Poopeiko, N. E.; Kvasyuk, E. I.; Mikhailopulo, I. A. Stereospecific Synthesis of β-D-Xylofuranosides of Adenine and Guanine. *Synthesis (Stuttg)* **1985**, *6* (7), 605–609. b) Martinková, M.; Mezeiová, E.; Gonda, J.; Jacková, D.; Pomikalová, K. Total Synthesis of (-)-Jaspine B and Its 4-Epi-Analogue from D-Xylose. *Tetrahedron Asymmetry* **2014**, *25* (9), 750–766.

(31) Chaudhary, S. K.; Hernandez, O. A simplified procedure for the preparation of triphenylmethylethers. *Tetrahedron Lett* **1979**, *2*, 95–98.

(32) De Luca, L.; Giacomelli, G.; Porcheddu, A. A Very Mild and Chemoselective Oxidation of Alcohols to Carbonyl Compounds. *Org. Lett.* **2001**, *3* (19), 3041–3043.

(33) Banks, M. R.; Cadogan, J. I. G.; Gosney, I.; Gould, R. O.; Hodgson, P. K. G.; Mcdougall, D. Preparation of Enantiomerically Pure Fructose-Derived 1,3-Oxazin-2-One by INIR Methodology and Its Application as a Chiral Auxiliary in Some Model Asymmetric Reactions. *Tetrahedron* **1998**, *54*, 9765–9784.

(34) Shariatipour, M.; Jadidinejad, M.; Heydari, A. A Green Synthesis of Nitrones in Glycerol. *J. Chem. Sci.* **2019**, *131*, 102.

(35) Richmond, E.; Ling, K. B.; Duguet, N.; Manton, L. B.; Çelebi-Ölçüm, N.; Lam, Y. H.; Alsancak, S.; Slawin, A. M. Z.; Houk, K. N.; Smith, A. D. An asymmetric pericyclic cascade approach to 3-alkyl-3-aryloxindoles: generality, applications and mechanistic investigations. *Org. Biomol. Chem.* **2015**, *13* (6), 1807–1817.

(36) Basak, A.; Ghosh, S. C. L-Proline-Mediated One-Pot Synthesis of 3-Exomethylene *β*-Lactams via Kinugasa Reaction. *Synlett* **2004**, *2004* (9), 1637–1639.

(37) Angelaud, R.; Zhong, Y. L.; Maligres, P.; Lee, J.; Askin, D. Synthesis of a β-Amino Acid Pharmacophore via a β-Lactam Intermediate. *Journal of Organic Chemistry* **2005**, *70* (5), 1949–1952.

(38) L. Krause, R. Herbst-Irmer, G. M. Sheldrick and D. Stalke, Comparison of silver and molybdenum microfocus X-ray sources for single-crystal structure determination, J. Appl. Crystallogr., 2015, 48, 3–10, DOI: 10.1107/ S1600576714022985.

(39) G. M. Sheldrick, Crystal structure refinement with SHELXL, Acta Crystallogr., Sect. C: Struct. Chem., 2015, 71, 3–8, DOI: 10.1107/S2053229614024218.

(40) O. V. Dolomanov, L. J. Bourhis, R. J. Gildea, J. A. K. Howard and H. Puschmann, OLEX2: a complete structure solution, refinement and analysis program, J. Appl. Crystallogr., 2009, 42, 339–341, DOI: 10.1107/S0021889808042726.

(41) G. M. Sheldrick, Crystal structure refinement with SHELXL, Acta Crystallogr., Sect. C: Struct. Chem., 2015, 71, 3–8, DOI: 10.1107/S2053229614024218.
